# Signaling-specific inhibition of the CB_1_ receptor for cannabis use disorder: phase 1 and phase 2a randomized trials

**DOI:** 10.1038/s41591-023-02381-w

**Published:** 2023-06-08

**Authors:** Margaret Haney, Monique Vallée, Sandy Fabre, Stephanie Collins Reed, Marion Zanese, Ghislaine Campistron, Caroline A. Arout, Richard W. Foltin, Ziva D. Cooper, Tonisha Kearney-Ramos, Mathilde Metna, Zuzana Justinova, Charles Schindler, Etienne Hebert-Chatelain, Luigi Bellocchio, Adeline Cathala, Andrea Bari, Roman Serrat, David B. Finlay, Filippo Caraci, Bastien Redon, Elena Martín-García, Arnau Busquets-Garcia, Isabelle Matias, Frances R. Levin, François-Xavier Felpin, Nicolas Simon, Daniela Cota, Umberto Spampinato, Rafael Maldonado, Yavin Shaham, Michelle Glass, Lars Lykke Thomsen, Helle Mengel, Giovanni Marsicano, Stéphanie Monlezun, Jean-Michel Revest, Pier Vincenzo Piazza

**Affiliations:** 1 Department of Psychiatry, Columbia University Irving Medical Center, New York State Psychiatric Institute, New York, NY USA; 2 University of Bordeaux, INSERM, Neurocentre Magendie, Bordeaux, France; 3 Aelis Farma, Bordeaux, France; 4 Behavioral Neuroscience Research Branch, National Institute on Drug Abuse Intramural Research Program, National Institutes of Health, Department of Health and Human Services, Baltimore, MD USA; 5 Department of Biology, University of Moncton, Moncton, NB Canada; 6 Department of Pharmacology and Toxicology, School of Biomedical Sciences, University of Otago, Dunedin, New Zealand; 7 Laboratory of Neuropharmacology, Department of Medicine and Life Sciences, University Pompeu Fabra, Barcelona, Spain; 8 Nantes Université, CNRS, CEISAM, UMR 6230, Nantes, France; 9 Aix Marseille Univ, APHM, INSERM, IRD, SESSTIM, Hop Sainte Marguerite, Service de Pharmacologie Clinique, Marseille, France; 10 Present Address: University of California, Los Angeles, Los Angeles, CA USA; 11 Present Address: Department of Drug and Health Sciences, University of Catania, Italy, and Oasi Research Institute-IRCCS, Unit of Translational Neuropharmacology, Troina, Italy; 12 Present Address: Basic Neuroscience Department, Université de Genève, Genève, Switzerland; 13 Present Address: Cell-Type Mechanisms in Normal and Pathological Behavior Research Group, Neuroscience Programme, IMIM Hospital del Mar Medical Research Institute, Barcelona, Spain

**Keywords:** Drug development, Receptor pharmacology, Reward

## Abstract

Cannabis use disorder (CUD) is widespread, and there is no pharmacotherapy to facilitate its treatment. AEF0117, the first of a new pharmacological class, is a signaling-specific inhibitor of the cannabinoid receptor 1 (CB_1_-SSi). AEF0117 selectively inhibits a subset of intracellular effects resulting from Δ^9^-tetrahydrocannabinol (THC) binding without modifying behavior per se. In mice and non-human primates, AEF0117 decreased cannabinoid self-administration and THC-related behavioral impairment without producing significant adverse effects. In single-ascending-dose (0.2 mg, 0.6 mg, 2 mg and 6 mg; *n* = 40) and multiple-ascending-dose (0.6 mg, 2 mg and 6 mg; *n* = 24) phase 1 trials, healthy volunteers were randomized to ascending-dose cohorts (*n* = 8 per cohort; 6:2 AEF0117 to placebo randomization). In both studies, AEF0117 was safe and well tolerated (primary outcome measurements). In a double-blind, placebo-controlled, crossover phase 2a trial, volunteers with CUD were randomized to two ascending-dose cohorts (0.06 mg, *n* = 14; 1 mg, *n* = 15). AEF0117 significantly reduced cannabis’ positive subjective effects (primary outcome measurement, assessed by visual analog scales) by 19% (0.06 mg) and 38% (1 mg) compared to placebo (*P* < 0.04). AEF0117 (1 mg) also reduced cannabis self-administration (*P* < 0.05). In volunteers with CUD, AEF0117 was well tolerated and did not precipitate cannabis withdrawal. These data suggest that AEF0117 is a safe and potentially efficacious treatment for CUD.

ClinicalTrials.gov identifiers: NCT03325595, NCT03443895 and NCT03717272.

## Abstract (web summary)

The signaling-specific inhibitor AEF0117 selectively inhibits CB_1_ activation by THC and reduces self-administration and subjective effects of cannabis in clinical trials, making it a potential treatment for cannabis use disorder.

## Main

Cannabis is the most widely used illicit drug in the world, and a meaningful subset of individuals who have used cannabis (19.5%) develop cannabis use disorder (CUD)^1^. In the United States^2^, 14.2 million individuals were diagnosed with CUD in 2020, and 14% of those receiving substance use disorder treatment reported cannabis as their primary drug of abuse^3^. Cannabis addiction, defined as a diagnosis of severe CUD^4^, is characterized by clinical impairment, such as failing to fulfill work or personal obligations, continuing to use cannabis despite it causing persistent problems and unsuccessful efforts to cut down^5^. In fact, few seeking treatment for CUD are able to achieve a substantial reduction in their cannabis use or abstain from cannabis use altogether^6^. However, despite an escalating need, there is no medication to facilitate CUD treatment^7^.

Cannabis’ effects are mainly mediated by its primary psychoactive ingredient, Δ^9^-tetrahydrocannabinol (THC), through stimulation of the type 1 cannabinoid receptor (CB_1_)^8^. CB_1_, the most expressed G-protein-coupled receptor in the brain^9^, is activated by endocannabinoids and plays a key modulatory role in processes such as pleasure, motivation, cognition and pain^10^, all of which are affected by cannabis use. We previously showed^11^ that the steroid pregnenolone is released in response to high concentrations of THC. Pregnenolone binds to a specific site on the CB_1_ and, without modifying ligand binding, inhibits a subset of intracellular responses triggered by CB_1_ activation. Specifically, pregnenolone inhibits CB_1_-mediated changes in mitogen-activated protein kinase (MAPK) phosphorylation and in mitochondrial respiration but does not modify CB_1_-mediated changes in cyclic adenosine monophosphate (cAMP), a prototypical cellular effect of CB_1_ agonists. Because of this signaling-specific action, pregnenolone inhibits many of THC’s effects without producing behavioral effects per se^11,12^.

These findings identify a potential therapeutic tool with a mechanism of action (MOA) that is superior to available pharmacological inhibitors of the CB_1_: orthosteric antagonists/inverse agonists^9^. By blocking CB_1_ agonist binding, CB_1_ antagonists inhibit all receptor activity, thereby impairing endocannabinoid function and producing serious adverse effects^13^. CB_1_ antagonists also precipitate withdrawal in THC-dependent animals^14^ and would be predicted to do so in patients with CUD, which precludes this as an approach to treat cannabis addiction.

However, pregnenolone is not a viable option as a pharmacotherapy because it is not a druggable compound^15^: it has a short half-life and low oral bioavailability, and it is rapidly converted into other active steroids that could produce adverse effects. For these reasons, we developed a new pharmacological class called ‘signaling-specific inhibitors of the CB_1_’ (CB_1_-SSi). These new molecular entities recapitulate the effects of pregnenolone but are not converted into other steroids and have highly favorable pharmacological and pharmaceutical characteristics.

Here we describe the development of AEF0117, the first CB_1_-SSi, from chemical design up to a proof-of-concept phase 2a study (NCT03717272) in research volunteers with CUD.

## Results

### Development and selection of AEF0117 as the first CB_1_-SSi

**Extended Data Fig. 1:**
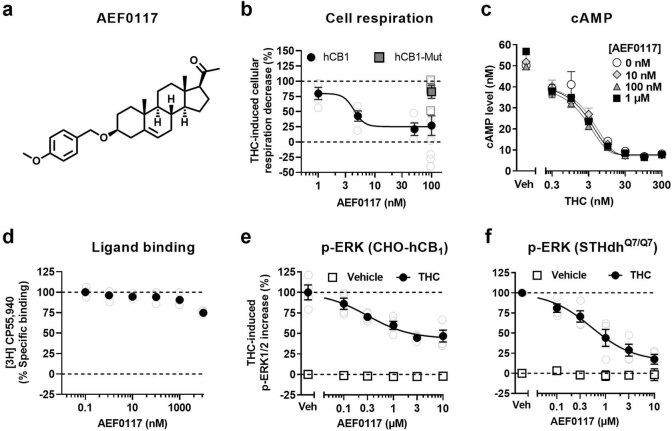
Structure and *in vitro* effects of AEF0117. (**a**) Chemical structure of AEF0117. (**b**) AEF0117 reversed THC-induced decreases in cellular respiration (1 μM) in HEK293 cells transfected with the human CB_1_ (hCB_1_). When cells are transfected with a mutant CB_1_ (hCB_1_-Mut), which invalidates the putative pregnenolone binding site, AEF0117 does not reverse THC’s effects (n = 3 and 4 for 2 and 1 independent experiments for THC, Vehicle, AEF0117 100 nM; n = 4 for all other conditions). (**c**) AEF0117 did not modify THC-induced inhibition of cAMP levels (n = 3 per dose). (**d**) AEF0117 did not modify binding of the CB_1_ agonist, CP55,940 (n = 3 per dose). (**e**) AEF0117 reduced the increase in p-ERK1/2 induced by THC (30 nM) in CHO cells stably transfected with the hCB_1_ (CHO-hCB_1_; n = 4 per dose). (**f**) AEF0117 reduced the increase in p-ERK1/2 induced by THC (10 µM) in STHdh^Q7/Q7^ cells expressing the mouse CB_1_ (4 independent experiments, n = 3 each). Data are represented as mean ± s.e.m.

To develop CB_1_-SSi, we hypothesized that it should be possible to obtain non-metabolizable pregnenolone derivatives by modifying pregnenolone at the carbons of the steroid ring (C3 and/or C17), which are targeted by endogenous enzymes to convert pregnenolone into other steroids^15^. We then built a library of these compounds and incubated each of them in cultures of Chinese hamster ovary (CHO) cell lines, which can metabolize pregnenolone into downstream steroids. Analysis of the cell culture medium with mass spectroscopy (MS) revealed several C3, C17-pregnenolone derivatives that were not metabolized into other steroids. These compounds were then screened in vitro for binding selectivity, toxicity in primary cultures of neurons and hepatocytes and genotoxicity (histone H2AX phosphorylation). Compounds that were selective and non-toxic were then used in micro-formulation experiments to identify a marketable formulation. We next conducted pharmacokinetic (PK) studies in mice measuring both plasma and brain concentrations after oral administration of these formulations and identified a subgroup of compounds that were selective and non-toxic in vitro and that had good oral bioavailability, PK characteristics and brain access. We then tested whether these compounds inhibited THC’s effects in vitro and in vivo. This last series of studies identified AEF0117—that is, 3β-(4-methoxybenzyloxy)pregn-5-en-20-one (Extended Data Fig. 1a)—as the best drug candidate for further development.

### Pharmacokinetics and toxicity of AEF0117 in animals

#### Pharmacokinetics of AEF0117

**Extended Data Fig. 2:**
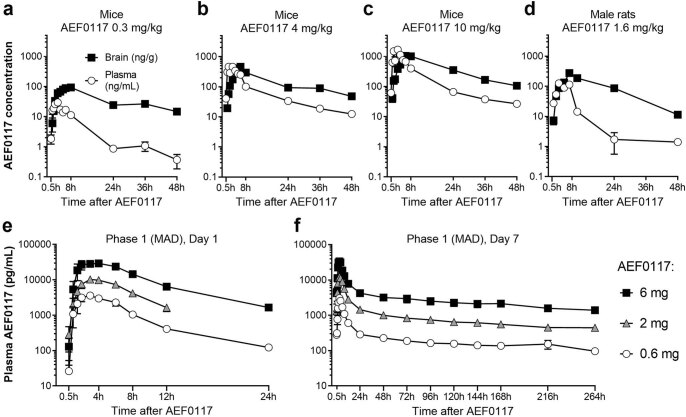
Pharmacokinetics of AEF0117 and THC in mice, rats, healthy volunteers, and volunteers with cannabis use disorder (CUD). Plasma and brain concentrations after a single *per os* administration of AEF0117 in male and female mice (n = 3 per sex and per time point, data for males and females were averaged) after different doses of AEF0117 (**a**) 0.3 mg/kg; (**b**) 4 mg/kg and (**c**) 10 mg/kg and (**d**) in male rats after 1.6 mg/kg of AEF0117 (n = 3 per time point). Plasma concentration of AEF0117 from the multiple ascending dose (MAD) study in healthy volunteers after: (**e**) the first dose (due to severe weather conditions, there was no sample available in the cohort of AEF0117 2 mg at 24 h); and (**f**) the seventh and last dose (n = 5 for 0.6 mg; n = 6 for the other dose cohorts). Data are represented as mean ± s.e.m.

AEF0117 is highly hydrophobic (logP 5.79 as predicted by ChemAxon Marvin Suite) and can be formulated in lipidic solvents. Corn oil provided the best absorption and PK characteristics after oral administration, with a T_max_ of 3 h in both mice and rats and a brain/plasma concentration area under the curve (AUC) ratio >4. These PK characteristics did not differ between males and females and were similar in mice, rats and dogs (Extended Data Fig. 2, Supplementary Table 1 and Supplementary Information), with good oral bioavailability (68% in dogs). In all species studied, the increase in maximum concentration (C_max_) and AUC was closely proportional to dose. In addition, plasma concentrations of AEF0117 were similar across species when applying allometric scaling based on body surface ratio, with the main difference being a longer terminal half-life in dogs (35.9 h) than in the other species.

#### Toxicity of AEF0117

AEF0117 did not show any adverse effects in safety pharmacology Good Laboratory Practice (GLP) tests: (1) tail current of hERG (human ether-a-go-go-related gene) in transfected HEK293 cells; (2) behavior (Irwin test) and body temperature in rats; (3) respiration in conscious rats; or (4) blood pressure, heart rate, electrocardiogram (ECG) and body temperature in conscious dogs. Similarly, AEF0117 did not show any genotoxic or mutagenic activity in GLP in vitro and in vivo studies.

In repeated (91-d) oral toxicity GLP studies in rats and dogs, AEF0117 had a no observed adverse effect level (NOAEL) > 65 mg kg^−1^ d^−1^ (Supplementary Information). Considering that the most observed 50% inhibitory dose (ID_50_) for inhibiting the effects of THC in mice and non-human primates is 5 µg kg^−1^ (Supplementary Table 2), AEF0117 has a therapeutic index (TI) > 13,000.

**Extended Data Fig. 3:**
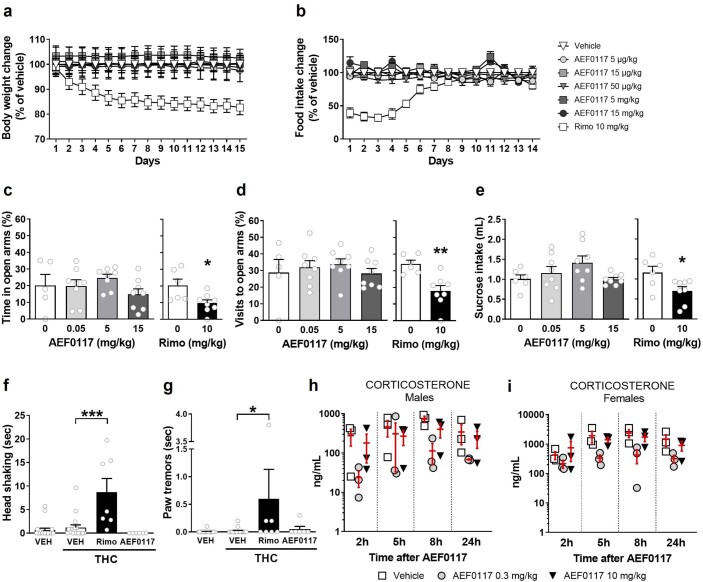
Effects of AEF0117 and of the CB_1_ orthosteric antagonist rimonabant in mice. Compared to Vehicle-treated animals, Rimonabant (Rimo 10 mg/kg; ip) but not AEF0117 (5, 15, 50 µg/kg and 5 and 15 mg/kg *per os*) decreased: (**a**) body weight in diet-induced obesity (DIO) mice, Days 5 to 15: P < 0.05 (from Day 5 to 15: P = 0.026, 0.0133, 0.0162, 0.008, 0.0076, 0.0053, 0.0054, 0.0044, 0.0027, 0.0032, 0.0018, respectively), Rimo vs Vehicle (Dunnett test) and (**b**) food intake in DIO mice, Day 1 to 5: P < 0.0001, Rimo vs Vehicle (Tukey test). N = 18 for vehicle; n = 10 for AEF0117 5 µg/kg, n = 7 for AEF0117 50 µg/kg, n = 8 for all other conditions. The repeated administration (28 days, once a day) of rimonabant (10 mg/kg) but not AEF0117 (0, 0.05, 5, 15 mg/kg, *per os*) increased anxiety- and depression-related behavior as shown by: the decrease in the percentage of (**c**) time spent in, and (**d**) visits to, the open arms of the Elevated Plus Maze (EPM) (for AEF0117: n = 5 for Vehicle, n = 8 for all other conditions; for Rimo: n = 6 for Vehicle, n = 8 for 10 mg/kg), *P = 0.0209, **P = 0.0026, Rimo 10 vs 0 mg/kg (unpaired *t*-test) and (**e**) the decrease in sucrose intake (n = 6 for Vehicle, n = 8 for all other conditions), *P = 0.0279, Rimo 10 vs 0 mg/kg (unpaired t-test). Rimonabant (10 mg/kg) but not AEF0117 (0.15 mg/kg) precipitated withdrawal in THC-dependent animals as shown by an increase in the duration of (**f**) head shaking and (**g**) paw tremors [n = 19 for Vehicles (VEH) groups, n = 7 for Rimo and n = 6 for AEF0117 groups], *P = 0.0351, ***P < 0.0001, Rimo vs Vehicle + THC (Dunnett test). AEF0117 (0.3 or 10 mg/kg, *per os*) did not significantly increased plasma concentrations of corticosterone (n = 3 per sex) in (**h**) male mice or (**i**) female mice. Data in **a** through **g** are represented as mean + s.e.m. Horizontal and vertical red lines in **h** and **i** represent mean ± s.e.m. respectively.

In mice, even high doses of AEF0117 did not produce any of the behavioral or neurohormonal effects (Extended Data Fig. 3) associated with CB_1_ antagonists that likely contribute to their poor tolerability: (1) reduced food intake; (2) increased anxiety-like and depression-like behaviors; (3) precipitated cannabinoid withdrawal; and (4) increased glucocorticoid secretion. These data, in combination with findings from the Irwin test, in which AEF0117 (0 mg kg^−1^, 2 mg kg^−1^, 9 mg kg^−1^ and 36 mg kg^−1^) had no effect on spontaneous behavior in the home cage (Supplementary Tables 3–8), show that AEF0117 has no identifiable effects on behavior per se in rodents.

#### Effects of AEF0117 on endocannabinoids and pregnenolone’s downstream steroids

**Extended Data Fig. 4:**
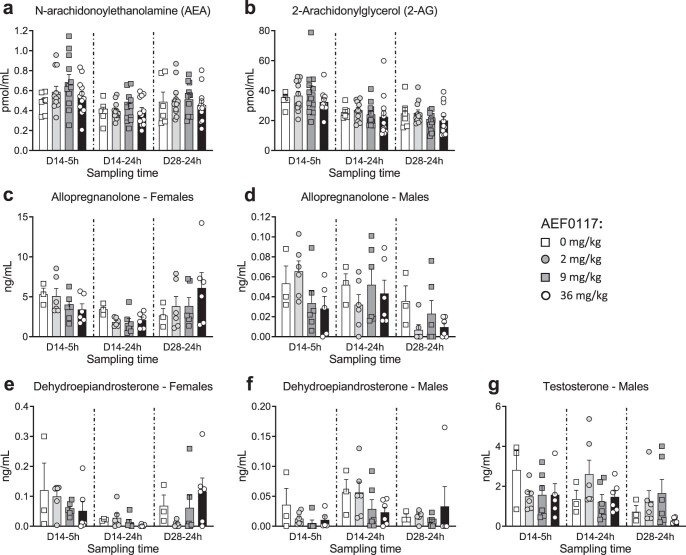
Effects of AEF0117 on endocannabinoids (AEA and 2-AG) and pregnenolone’s downstream steroids (allopregnanolone, DHEA and testosterone) in male and female rats. (**a**) N-arachidonoylethanolamine (Anandamide, AEA) and (**b**) 2-Arachidonylglycerol (2-AG) data were averaged for males and females. (**c**) Allopregnanolone in females. (**d**) Allopregnanolone in males. (**e**) Dehydroepiandrosterone (DHEA) in females. (**f**) DHEA in males. (**g**) Testosterone in males. For all parameters, the timepoints were: 14 days of treatment at 5 h (D14-5h) and 24 h (D14-24h) after dosing with AEF0117, and 24 h after the last administration on day 28 (D28-24h). For (**a**) and (**b**) n = 6 per time point for vehicle (AEF0117 0 mg/kg), n = 12 per time point for all the other doses of AEF0117. For (**c-g**), n = 3 per time point for vehicle (AEF0117 0 mg/kg), n = 6 per time point for the other doses of AEF0117 (2, 9, 36, mg/kg) except n = 5 for AEF0117 36 mg/kg at D14-5h time point in (**d, f, g**) and n = 5 for AEF0117 36 mg/kg at D28-24h in (**g**). Data are represented as mean + s.e.m.

**Extended Data Fig. 5:**
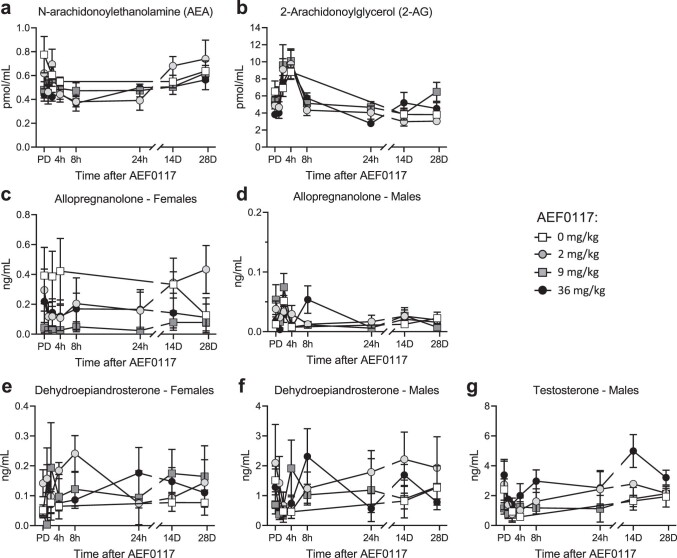
Effects of AEF0117 on endocannabinoids (AEA and 2-AG) and pregnenolone’s downstream steroids (allopregnanolone, DHEA and testosterone) in male and female dogs. (**a**) N-arachidonoylethanolamine (Anandamide, AEA) and (**b**) 2-Arachidonylglycerol (2-AG) data were averaged in males and females. (**c**) Allopregnanolone in females. (**d**) Allopregnanolone in males. (**e**) Dehydroepiandrosterone (DHEA) in females. (**f**) DHEA in males. (**g**) Testosterone in males. PD= Pre-dose levels. n = 3 per sex per time point. Data are represented as mean ± s.e.m.

AEF0117 did not increase plasma endocannabinoid (AEA and 2-AG) levels in rats (Extended Data Fig. 4a,b) or dogs (Extended Data Fig. 5a,b). AEF0117 seemed metabolically stable and was not converted into pregnenolone’s downstream steroids, testosterone, dheadehydroepiandrosterone and allopregnanolone in either rats (Extended Data Fig. 4c–g) or dogs (Extended Data Fig. 5c–g).

### Preclinical proof of concept of AEF0117

#### AEF0117 acted as a CB_1_-SSi in vitro

When tested in a Eurofins high-throughput screen for binding activity at 85 receptors, including the major steroid receptors, AEF0117 (10 µM) did not modify binding to any receptor. In this respect, AEF0117 was more selective than pregnenolone (10 µM), which displaced (>80%) binding to the glucocorticoid, androgen and progesterone receptors and, to a lesser extent (>40%), binding to the peripheral benzodiazepine receptor (Supplementary Table 9).

Like pregnenolone, AEF0117 potently (IC_50_ 3 nM) inhibited the decrease in cellular respiration induced by THC (1 µM) in HEK293 cells transfected with the human CB_1_ (hCB_1_) (Extended Data Fig. 1b) without modifying THC’s effects on cAMP (Extended Data Fig. 1c). AEF0117 bound to the same region of the CB_1_ as pregnenolone, as shown by the loss of effect on cellular respiration (Extended Data Fig. 1b) when HEK293 cells were transfected with a single point mutant of hCB_1_ (p.E1.49G) shown^11^ to invalidate the pregnenolone binding site. The putative binding site of AEF0117 is not near the orthosteric binding site^11^, consistent with the observation that AEF0117 does not modify the equilibrium binding of the CB_1_ agonist [^3^H]CP55,940 in cell membranes from HEK293-hCB_1_ cells (Extended Data Fig. 1d). The inhibitory effects of AEF0117 on THC-induced phosphorylation of MAPK (p-ERK1/2), another prototypical effect of pregnenolone, was validated using two cell lines: CHO-hCB_1_ cells, in which hCB_1_ has been stably transfected (Extended Data Fig. 1e), and STHdh^Q7/Q7^ cells (Extended Data Fig. 1f), which express endogenous mouse CB_1_. AEF0117 inhibited THC-induced p-ERK1/2 in both cell lines with similar potency (IC_50_ 300 nM), but the extent of inhibition was more pronounced in STHdh^Q7/Q7^ cells (Extended Data Fig. 1f).

#### AEF0117 inhibited the effects of THC related to cannabis addiction in vivo

The effects of AEF0117 were studied in several animal species using experimental models relevant to cannabis addiction and to cannabis-related behavioral disruption. For all experiments, we administered AEF0117 orally 3 h before THC administration, corresponding to the plasma T_max_ of AEF0117 (Supplementary Table 1). In some experiments, full dose–response functions were obtained, which identified the effective dose of AEF0117 to use in other experiments. AEF0117 potently inhibited the effects of THC, with an ID_100_ that ranged between 15 µg kg^−1^ (most frequently observed ID_100_) and 1.5 µg kg^−1^ (in fewer cases) depending on the behavior studied (Supplementary Table 2).

**Fig. 1:**
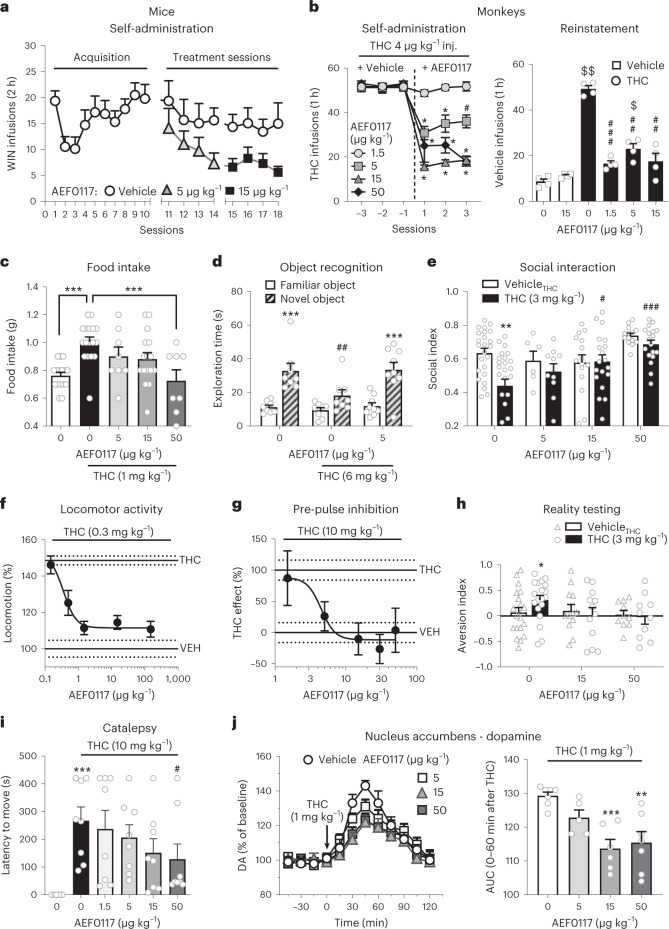
AEF0117 decreased the behavioral and physiological effects of cannabinoids. **a**, In mice, after the acquisition phase (left panel, *n* = 26), AEF0117 (right panel, *n* = 13) decreased the number of infusions of the CB_1_ agonist WIN55,212-2 compared to vehicle-treated mice (*n* = 13); *P* = 0.021: ANOVA treatment effects for AEF0117 (15 µg kg^−1^). **b**, In monkeys (left panel, *n* = 4), AEF0117 dose-dependently decreased the number of THC infusions (4 µg kg^−1^ per infusion). *P < 0.001, ^#^*P* = 0.002 compared to vehicle (three sessions average), Tukey test. In monkeys (right panel, *n* = 4), after extinction of THC-reinforced responding, AEF0117 decreased reinstatement of drug seeking induced by a non-contingent THC injection (40 µg kg^−1^, i.v.). ^$^*P* = 0.0346, ^$$^P = 0.0015 compared to non-contingent saline; ^##^*P* = 0.0023, *P* = 0.0018 in AEF0117 5 µg kg^−1^ and 15 µg kg^−1^, respectively, ^###^*P* = 0.0002 compared to THC + vehicle (AEF0117 0 µg kg^−1^), Dunnett test, within-subjects design. AEF0117 inhibited the following effects of THC in mice. **c**, Increase in food intake, ^###^*P* = 0.0003, ^***^P = 0.0007 (Dunnett test, *n* = 16 for vehicle and AEF0117 15 µg kg^−1^, *n* = 17 for THC, *n* = 8 for the other conditions). **d**, Impairment of long-term memory, ***P < 0.001: familiar versus novel object (*P* = 0.0002 for vehicle + AEF0117 0 µg kg^−1^ and *P* < 0.0001 for THC + AEF0117 5 µg kg^−1^); ^*##*^P < 0.01: novel object after THC + AEF0117 0 µg kg^−1^ versus novel object in the other two conditions (*P* = 0.0082 versus vehicle + AEF0117 0 µg kg^−1^ and *P* = 0.0041 versus THC + AEF0117 5 µg kg^−1^), Sidak test, *n* = 8 for vehicle and THC, *n* = 9 for AEF0117. **e**, Decrease in social interaction, ^**^P = 0.0001: THC versus vehicle; ^#^*P* = 0.0068, ^###^*P* < 0.0001: AEF0117 + THC versus THC (Dunnett test, *n* = 24, 6, 15 and 14 for AEF0117 0 μg kg^−1^, 5 μg kg^−1^, 15 μg kg^−1^ and 50 μg kg^−1^ + vehicle; *n* = 27, 10, 19 and 18 for AEF0117 0 μg kg^−1^, 5 μg kg^−1^, 15 μg kg^−1^ and 50 μg kg^−1^ + THC, respectively). **f**, Increase in locomotor activity, *P* < 0.0001: treatment effect ANOVA (*n* = 18 for vehicle and THC, *n* = 19 for AEF0117 1.5 μg kg^−1^, *n* = 10 for the other conditions). **g**, Impairment of sensory gating, *P* = 0.001: treatment effect ANOVA (*n* = 18 for vehicle and THC, *n* = 9 for AEF0117 50 μg kg^−1^, *n* = 10 for the other conditions). **h**, Impairment in reality testing, ^*^P = 0.0377: THC versus vehicle (unpaired *t*-test, one-tailed, *n* = 20 for AEF0117 0 μg kg^−1^ vehicle, *n* = 19 for AEF0117 0 μg kg^−1^ THC, *n* = 12 and 10 for AEF0117 15 μg kg^−1^ and 50 μg kg^−1^ per treatment dose). **i**, Catalepsy, ^***^P = 0.0002: THC versus vehicle; ^#^*P* = 0.037: AEF0117 + THC versus THC (Mann–Whitney test, *n* = 8 per condition). **j**, In rats, left: percentage increase in extracellular DA concentration from baseline over time; right: AUC of extracellular DA concentrations, ^**^P = 0.0017, ^***^P = 0.0008: AEF0117 + THC versus THC (Dunnett test, *n* = 7 for vehicle and *n* = 5, 6 and 7 for AEF0117 5 μg kg^−1^, 15 μg kg^−1^ and 50 μg kg^−1^, respectively). Data are represented as mean ± s.e.m. inj., injection.

AEF0117 (15 µg kg^−1^) significantly reduced intravenous (i.v.) self-administration of the CB_1_ agonist WIN55,212-2 in CD-1 Swiss male mice (Fig. 1a) and of THC in male squirrel monkeys (Fig. 1b, left panel). Rodents do not self-administer i.v. THC but do self-administer more efficacious CB_1_ agonists, such as WIN55,212-2. During i.v. drug self-administration, a model of drug reinforcement used to assess potential pharmacotherapies for substance use disorders^16^, laboratory animals learn to provide an operant response (nose poking for mice, lever pressing for monkeys) to obtain a drug infusion. In addition to decreasing self-administration, AEF0117 (1.5 µg kg^−1^) also significantly reduced (Fig. 1b, right panel) the reinstatement of THC seeking in monkeys after THC-reinforced responding was extinguished. The reinstatement of THC seeking after an injection of THC is an animal model of drug relapse^17^.

**Fig. 2:**
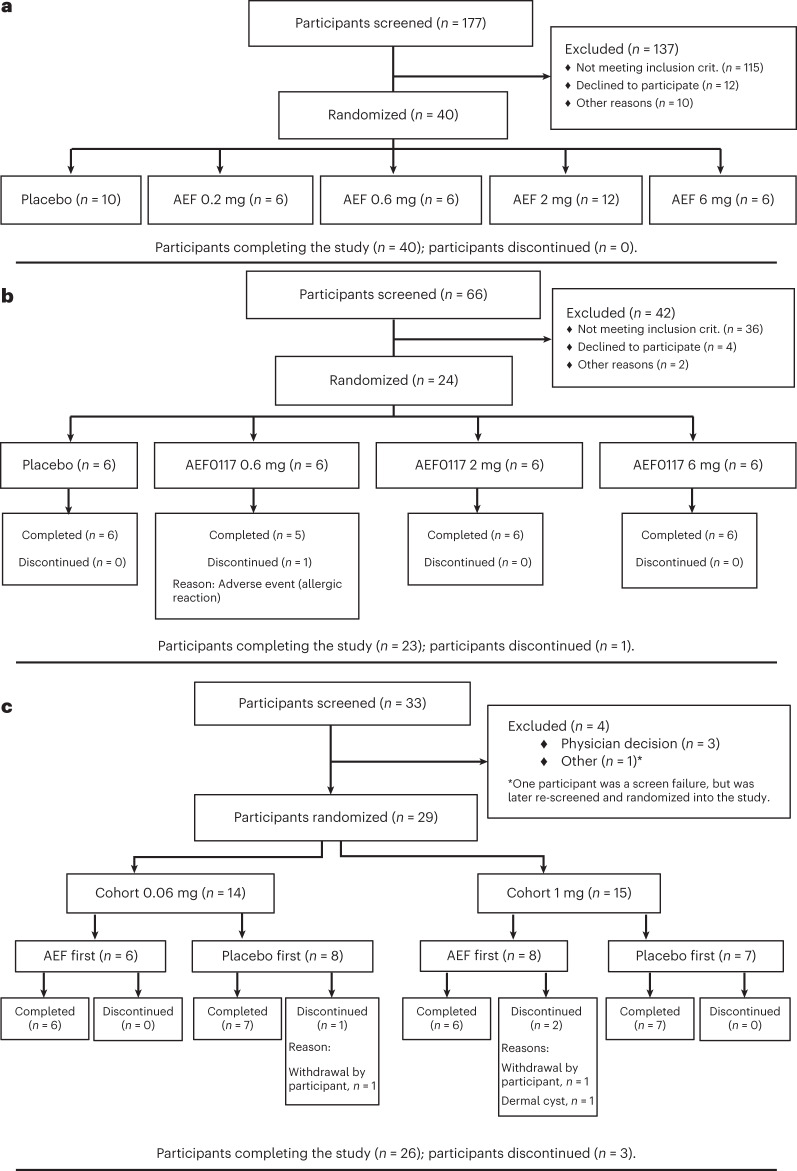
Distribution of participants. Participant flow for the SAD (**a**), MAD (**b**) and phase 2a (**c**) studies. AEF, AEF0117; crit., criteria.

**Fig. 3:**
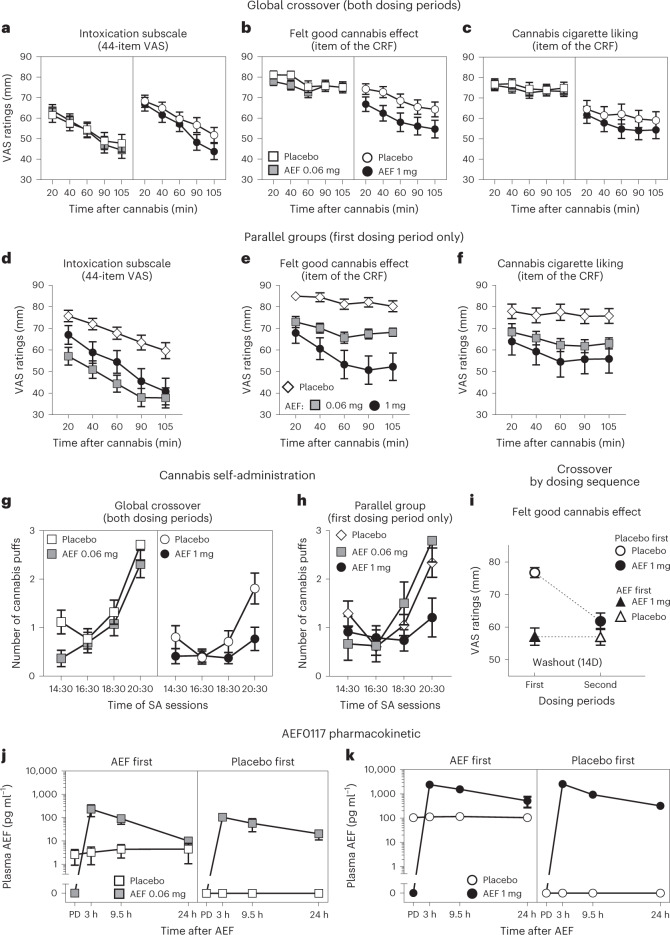
AEF0117 decreased positive subjective ratings and cannabis self-administration in research volunteers with CUD in the phase 2a study. AEF0117 (1 mg) significantly decreased positive subjective ratings of cannabis measured by VAS (0–100 mm). **a**,**d**, Intoxication subscale comprising the arithmetic mean of the ratings of the ‘I feel a Good Effect’ and ‘I feel High’ items. Rating of the ‘Felt Good Cannabis Effect’ (**b**,**e**) and of the ‘Cannabis Cigarette Liking’ (**c**,**f**) items. **a**–**c**, MMRM global crossover analysis (*n* = 13 per dose) shows a significant effect of treatment for (Treatment × Time, *P* = 0.0036 and Dose × Treatment × Time, *P* = 0.0017) (**a**) and (Treatment × Dose × Time, *P* = 0.0034) (**b**). Significant Treatment × Sequence interactions were found for all the outcomes, *P* = 0.0182 (**a**), *P* < 0.0001 (**b**) and *P* = 0.0318 (**c**). **d**–**f**, When the pre-planned MMRM parallel group analysis (first dosing period only, placebo *n* = 14, AEF0117 *n* = 6 per dose) was performed to eliminate the sequence effect, a significant decrease was observed for all the outcomes (Treatment × Time, *P* = 0.0381 (**d**), *P* = 0.0032 (**e**), *P* = 0.0126 (**f**); Treatment × Dose × Time, *P* = 0.0368 (**e**)). **g**,**h**, AEF0117 1 mg significantly decreased cannabis self-administration as measured by the number of cannabis puffs purchased by the participants. **g**, The MMRM global crossover analysis (*n* = 13 per dose) showed a significant Treatment effect (*P* = 0.0254), a Treatment × Dose × Session interaction (*P* = 0.0009) and a Treatment × Sequence interaction (*P* = 0.0085). **h**, The pre-planned MMRM parallel group analysis showed a significant decrease in self-administration (Dose × Treatment × Session, *P* = 0.0344). **i**, Exploratory analysis as a function of the dosing sequence (*n* = 6 for AEF0117 1 mg first or *n* = 7 for placebo first) for ‘Felt Good Cannabis Effect’. The ratings after AEF0117 were similar in the two sequences, whereas responding under placebo was lower if participants had previously received AEF0117, indicating a long-lasting effect of AEF0117. **j**,**k**, When AEF0117 was administered in the first study period (AEF0117 first, *n* = 6 for the 0.06-mg cohort; *n* = 7 and 8 for the 1-mg cohort for placebo and AEF0117, respectively), detectable levels of AEF0117 were observed in the second dosing period when participants received placebo ≥14 d after AEF0117 administration. This was not the case when AEF0117 was administered in the second dosing period (placebo first, *n* = 8 and 7 per dose for the 0.06-mg and 1-mg cohorts, respectively). Data are represented as mean ± s.e.m. over the different days of testing for each timepoint (**a**–**h**,**j**,**k**) or as overall rating over the 5 d of testing and the five timepoints (**i**). AEF, AEF0117; D, days; SA, self-administration; PD, pre-dose.

Several measures of behavioral disruption produced by THC were studied in mice (Supplementary Table 2). AEF0117 inhibited THC’s effects on food intake (Fig. 1c), long-term object recognition memory (Fig. 1d) and social interaction (Fig. 1e). AEF0117 also inhibited THC’s effects on a range of behaviors hypothesized to model psychotic symptomatology^18^, such as (1) increased psychomotor stimulation (Fig. 1f); (2) impairment in sensory motor gating, as measured by pre-pulse inhibition (PPI), an outcome also altered in schizophrenia (Fig. 1g); (3) impairment in a test evaluating the perception of external stimuli (reality testing) (Fig. 1h); and (4) catalepsy (Fig. 1i), a potential model of catatonia observed in psychosis and after the use of certain synthetic cannabinoids^19^.

AEF0117 (15 µg kg^−1^) also inhibited THC-induced increases in nucleus accumbens (Nac) extracellular dopamine (DA) in freely moving rats (Fig. 1j), one of the cellular changes mediating the rewarding effects of cannabinoids^20,21^.

### Phase 1 studies in healthy volunteers

#### Safety data

AEF0117 administration was safe and well tolerated in two double-blind, placebo-controlled studies in healthy volunteers: (1) a single-ascending dose (SAD; NCT03325595) study testing 0.2 mg, 0.6 mg, 2 mg and 6 mg of AEF0117 (*n* = 40; Fig. 2a and Table 1) and (2) a multiple-ascending dose (MAD; NCT03443895) study testing 0.6 mg, 2 mg and 6 mg, once a day for 7 d (*n* = 24; Fig. 2b and Table 1). In both studies, most volunteers were male (90–91%), Black (67–85%) and non-Hispanic (83–90%). Mean age (36.8–38.1 years) and body mass index (BMI) (25.2–25.7 kg m^−2^) were also similar in both studies. No major differences were observed between treatment groups except for sex given that, due to their limited enrollment, females were not represented at each dose.

**Table 1:** Baseline demographic characteristics and cannabis use history for participants in the SAD, MAD and phase 2a studies

		SAD study						MAD study					Phase 2a study		
AEF0117 dose (*n*)		Placebo (10)	0.2 mg (6)	0.6 mg (6)	2 mg (12)	6 mg (6)	Total (40)	Placebo (6)	0.6 mg (6)	2 mg (6)	6 mg (6)	Total (24)	0.06 mg (14)	1 mg (15)	Total (29)
**Demographic data**															
Sex															
Male	*n* (%)	9 (90.0)	6 (100)	6 (100)	9 (75.0)	6 (100)	36 (90.0)	6 (100)	4 (66.7)	6 (100)	6 (100)	22 (91.7)	14 (100)	14 (93.3)	28 (96.6)
Female	*n* (%)	1 (10.0)	0	0	3 (25.0)	0	4 (10.0)	0	2 (33.3)	0	0	2 (8.3)		1 (6.7)	1 (3.4)
Race															
Black	*n* (%)	9 (90.0)	4 (66.7)	5 (83.3)	10 (83.3)	6 (100)	34 (85.0)	6 (100)	2 (33.3)	4 (66.7)	4 (66.7)	16 (66.7)	7 (50.0)	10 (66.7)	17 (58.6)
White	*n* (%)	1 (10.0)	2 (33.3)	1 (16.7)	1 (8.3)	0	5 (12.5)	0	2 (33.3)	2 (33.3)	2 (33.3)	6 (25.0)	2 (14.3)	2 (13.3)	4 (13.8)
Mixed race	*n* (%)	0	0	0	1 (8.3)	0	1 (2.5)	0	2 (33.3)	0	0	2 (8.3)	5 (35.7)	3 (20.0)	8 (27.6)
Ethnicity															
Hispanic	*n* (%)	0	1 (16.7)	1 (16.7)	1 (8.3)	1 (16.7)	4 (10.0)	0	2 (33.3)	1 (16.7)	1 (16.7)	4 (16.7)	6 (42.8)	6 (40.0)	12 (41.4)
Not Hispanic	*n* (%)	10 (100)	5 (83.3)	5 (83.3)	11 (91.7)	5 (83.3)	36 (90.0)	6 (100)	4 (66.7)	5 (83.3)	5 (83.3)	20 (83.3)	8 (57.1)	9 (60.0)	17 (58.6)
Age (years)															
	Mean (s.d.)	35.2 (9.6)	35.3 (8.8)	40.8 (10.6)	35.5 (10.3)	39.3 (10.0)	36.8 (9.6)	37.3 (12.3)	36.5 (9.4)	43.3 (11.0)	35.2 (8.0)	38.1 (10.1)	32.6 (6.1)	32.3 (6.6)	32.5 (6.3)
	Min, Max	22, 53	22, 46	23, 55	22, 53	30, 54	22, 55	20, 53	26, 52	32, 55	25, 48	20, 55	24, 42	21, 44	21, 44
BMI (kg m^−^^2^)															
	Mean (s.d.)	25.8 (2.6)	24.0 (2.7)	26.3 (3.0)	25.5 (2.2)	23.7 (3.0)	25.2 (2.6)	24.7 (2.4)	27.6 (1.4)	26.1 (2.8)	24.4 (3.1)	25.7 (2.7)	24.7 (4.1)	23.9 (3.0)	24.3 (3.5)
	Min, Max	22.0, 29.6	21.1, 27.7	21.6, 29.2	22.7, 29.5	19.3, 29.6	19.3, 29.6	22.4, 28.5	24.7, 28.6	20.9, 29.5	20.6, 28.1	20.6, 29.5	21.6, 32.0	18.7, 29.6	18.7, 32.0
**Cannabis use history**															
CUD severity															
Mild	*n* (%)	–	–	–	–	–	–	–	–	–	–	–	5 (35.7)	5 (33.3)	10 (34.5)
Moderate	*n* (%)	–	–	–	–	–	–	–	–	–	–	–	5 (35.7)	8 (53.3)	13 (44.8)
Severe	*n* (%)	–	–	–	–	–	–	–	–	–	–	–	4 (28.6)	2 (13.3)	6 (20.7)
Current mean number of grams of cannabis per day															
	Mean (s.d.)	–	–	–	–	–	–	–	–	–	–	–	2.4 (1.3)	3.3 (1.9)	2.9 (1.7)
	Min, Max	–	–	–	–	–	–	–	–	–	–	–	1.0, 4.7	1.5, 8.0	1.0, 8.0
Current mean number of days of cannabis use per week															
	Mean (s.d.)	–	–	–	–	–	–	–	–	–	–	–	6.8 (0.5)	6.9 (0.3)	6.9 (0.4)
	Min, Max	–	–	–	–	–	–	–	–	–	–	–	5, 7	6, 7	5, 7

No treatment-related serious adverse events (SAEs) and a limited number of treatment-emergent adverse events (TEAEs), showing no dose dependency, were reported (Table 2). There were no clinically relevant drug-related adverse events (AEs), except for one moderate episode of pruritus and cutaneous rash after the first administration of 0.6 mg of AEF0117 in the MAD study (treatment for this volunteer was discontinued). No potentially clinically significant abnormalities (PCSAs) in vital signs, ECG or safety laboratory parameters were observed either (Table 2), except for one asymptomatic increase in creatine phosphokinase (CPK) and myoglobin observed 6 d after dosing with 2 mg of AEF0117 in the SAD study. Because of this, the 2-mg cohort was repeated. No increase in CPK was observed in the second 2-mg cohort or in any other research volunteer.

**Table 2:** Safety data of participants in the SAD, MAD and Phase 2a studies

		SAD study						MAD study					Phase 2a study				
AEF0117 dose (*n*)		Placebo (10)	0.2 mg (6)	0.6 mg (6)	2 mg (12)	6 mg (6)	Total (40)	Placebo (6)	0.6 mg (6)	2 mg (6)	6 mg (6)	Total (24)	0.06 mg (14)	Placebo (14)	1 mg (15)	Placebo (15)	Total (29)
**TEAEs**																	
Number of TEAEs	E	1	4	0	2	0	7	1	7	3	6	17	9	18	24	18	69
Number of participants with at least one TEAE	*n* (%)	1 (10.0)	2 (33.3)	0	1 (8.3)	0	4 (10.0)	1 (16.7)	3 (50.0)	2 (33.3)	2 (33.3)	8 (33.3)	6 (42.9)	8 (57.1)	8 (53.3)	8 (53.3)	21 (72.4)
Related	*n* (%)	1 (10.0)	1 (16.7)	0	1 (16.7)	0	3 (7.5)	1 (16.7)	3 (50.0)	1 (16.7)	2 (33.3)	7 (29.2)	6 (42.9)	6 (42.9)	8 (53.3)	8 (53.3)	19 (65.5)
Not related	*n* (%)	0	1 (16.7)	0	0	0	1 (2.5)	0	1 (16.7)	1 (16.7)	2 (33.3)	4 (16.7)	2 (14.3)	4 (28.6)	1 (6.7)	0	6 (20.7)
Mild	*n* (%)	1 (10.0)	2 (33.3)	0	1 (8.3)	0	4 (10.0)	1 (16.7)	2 (33.3)	2 (33.3)	2 (33.3)	7 (29.2)	6 (42.9)	8 (57.1)	8 (53.3)	7 (46.7)	20 (69.0)
Moderate	*n* (%)	0	1 (16.7)	0	0	0	1 (2.5)	0	1 (16.7)	0	1 (16.7)	2 (8.3)	0	1 (7.1)	1 (6.7)	1 (6.7)	3 (10.3)
Severe	*n* (%)	0	0	0	0	0	0	0	0	0	0	0	0	0	1 (6.7)	0	1 (3.4)
Number of participants with an SAE	*n* (%)	0	0	0	0	0	0	0	0	0	0	0	0	0	0	0	0
Number of participants with a TEAE leading to discontinuation	*n* (%)	0	0	0	0	0	0	0	1 (16.7)	0	0	1 (4.2)	0	0	1 (6.7)	0	1 (3.4)
**TEAEs by system organ class**																	
Nervous system disorders	*n* (%)	0	1 (16.7)	0	0	0	1 (2.5)	1 (16.7)	2 (33.3)	0	2 (33.3)	5 (20.8)	3 (21.4)	2 (14.3)	3 (20.0)	2 (13.3)	9 (31.0)
Investigations	*n* (%)	0	0	0	1 (8.3)	0	1 (2.5)	0	0	1 (16.7)	0	1 (4.2)	3 (21.4)	3 (21.4)	2 (13.3)	2 (13.3)	8 (27.6)
Gastrointestinal disorders	*n* (%)	1 (10.0)	2 (33.3)	0	0	0	3 (7.5)	0	0	1 (16.7)	0	1 (4.2)	0	3 (21.4)	4 (26.7)	3 (20.0)	7 (24.1)
Skin and subcutaneous tissue disorders	*n* (%)	–	–	–	–	–	–	0	1 (16.7)	0	1 (16.7)	2 (8.3)	0	0	1 (6.7)	0	1 (3.4)
General disorders and administration site conditions	*n* (%)	–	–	–	–	–	–	0	1 (16.7)	0	1 (16.7)	2 (8.3)	–	–	–	–	–
Metabolism and nutrition disorders	*n* (%)	–	–	–	–	–	–	0	1 (16.7)	0	0	1 (4.2)	–	–	–	–	–
Musculoskeletal and connective tissue disorders	*n* (%)	–	–	–	–	–	–	–	–	–	–	–	0	2 (14.3)	2 (13.3)	1 (6.7)	4 (13.8)
Injury, poisoning and procedural complications	*n* (%)	–	–	–	–	–	–	–	–	–	–	–	0	1 (7.1)	2 (13.3)	0	3 (10.3)
General disorders and administration site conditions	*n* (%)	–	–	–	–	–	–	–	–	–	–	–	1 (7.1)	0	0	1 (6.7)	2 (6.9)
Psychiatric disorders	*n* (%)	–	–	–	–	–	–	–	–	–	–	–	0	0	2 (13.3)	0	2 (6.9)
Renal and urinary disorders	*n* (%)	–	–	–	–	–	–	–	–	–	–	–	0	1 (7.1)	0	1 (6.7)	2 (6.9)
Respiratory, thoracic and mediastinal disorders	*n* (%)	–	–	–	–	–	–	–	–	–	–	–	1 (7.1)	0	0	1 (6.7)	2 (6.9)
Cardiac disorders	*n* (%)	–	–	–	–	–	–	–	–	–	–	–	1 (7.1)	0	0	0	1 (3.4)
Ear and labyrinth disorders	*n* (%)	–	–	–	–	–	–	–	–	–	–	–	0	0	0	1 (6.7)	1 (3.4)
Infections and infestations	*n* (%)	–	–	–	–	–	–	–	–	–	–	–	0	1 (7.1)	0	0	1 (3.4)
Eye disorder	*n* (%)	–	–	–	–	–	–	0	0	0	1 (16.7)	1 (4.2)	–	–	–	–	–

In addition, AEF0117 did not significantly alter mood ratings or behavioral measures relative to placebo. No trends were observed in psychometric tests (Bond & Lader Visual Analog Scales (VAS), Profile of Mood States 65 and Columbia-Suicide Severity Rating Scale; Supplementary Tables 10–15) that gauge moods, including depression, anxiety and suicidality. AEF0117 also did not differ from placebo (Supplementary Tables 16 and 17) on a range of subjective effects measured using the Addiction Research Center Inventory (ARCI 49).

#### PK characteristics

The PK characteristics of AEF0117 in healthy volunteers (Supplementary Table 18) were consistent with those observed in animals. The T_max_ was approximately 3 h, and C_max_ and AUC showed dose proportionality. In addition, plasma concentrations of AEF0117 in humans were in the range of those observed in animals when allometric scaling based on body surface ratio was applied. The major difference between species was a considerably longer terminal half-life in humans (152–258 h in the MAD study) than in the other species, including dogs.

#### Effects on endocannabinoids and pregnenolone’s downstream steroids

**Extended Data Fig. 6:**
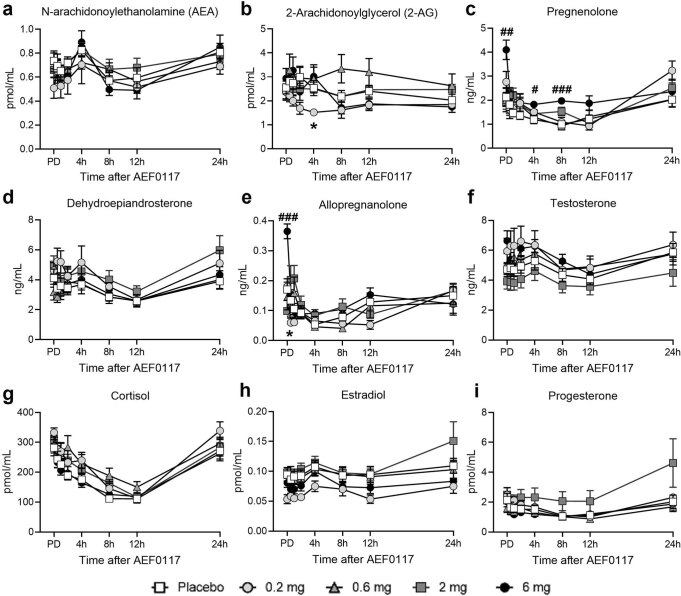
Effects of single doses of AEF0117 on endocannabinoids (AEA and 2-AG), pregnenolone, and pregnenolone’s downstream steroids in male and female healthy volunteers. (**a**) N-arachidonoylethanolamine (Anandamide, AEA). (**b**) 2- Arachidonoylglycerol (2-AG). (**c**) Pregnenolone. (**d**) Dehydroepiandrosterone (DHEA). (**e**) Allopregnanolone. (**f**) Testosterone (only males). (**g**) Cortisol. (**h**) Estradiol. (**i**) Progesterone. Dunnett’s multiple comparisons test after ANOVA were performed. There were statistically significantly higher levels of pregnenolone (**c**, # P = 0.0306; ## P = 0.0051; ### P < 0.0001) in the AEF0117 6 mg group compared to placebo, but this difference was already observed at pre-dose, suggesting an effect independent of AEF0117 dosing. Higher pre-dose levels in the 6 mg group compared to placebo were also observed for allopregnanolone (**e**, ### P = 0.0006). In the 0.2 mg group compared to placebo there were lower concentrations of 2-AG 4 h after dosing (**b**, * P = 0.0223) and lower concentrations of allopregnanolone at 0.5 h after dosing (**e**, * P = 0.0467). Data are represented as mean ± s.e.m. Single ascending dose (SAD) and day 1 results of the multiple ascending dose (MAD) studies were averaged, n = 16 for Placebo per time point n = 6 per 0.2 mg/kg per time point, n = 12 [except n = 10 in (**f**)] per 0.6 mg/kg per time point, n = 18 per 2 mg/kg per time point and n = 12 per 6 mg/kg per time point. PD = Pre-dose levels.

A single administration of AEF0117 did not increase plasma endocannabinoid (AEA and 2-AG) levels in the healthy volunteers in the SAD and MAD studies (Extended Data Fig. 6a,b). The only significant effect of treatment was a decrease in 2-AG 4 h after administration of AEF0117 (0.2 mg) relative to placebo.

As in the preclinical studies, AEF0117 was not converted into pregnenolone’s downstream steroids: testosterone, dehydroepiandrosterone, allopregnanolone, cortisol, estradiol and progesterone (Extended Data Fig. 6d–i). The only significant effect of treatment relative to placebo was a decrease in allopregnanolone (Extended Data Fig. 6e) at a single timepoint (0.5 h) after AEF0117 (0.2 mg) administration. Pregnenolone levels (Extended Data Fig. 6c) were significantly higher after 6-mg AEF0117 compared to placebo at 4 h and 8 h after dosing. However, the 6-mg AEF0117 group had significantly higher pre-dose levels of pregnenolone, suggesting that these differences were not caused by AEF0117 administration but, rather, reflected random variation in, for example, baseline levels of stress or in intradiurnal fluctuations in pregnenolone levels for this group of participants.

### Phase 2a study in research volunteers with CUD

In this phase 2a study (NCT03717272) conducted according to Good Clinical Practice (GCP), the effects of AEF0117 were studied in a randomized, double-blind, placebo-controlled, crossover, multiple-dose-escalation study in non-treatment-seeking male and female cannabis-smoking (≥6 d per week; ≥1 g of cannabis per day) research volunteers with CUD (Fig. 2c and Table 1). Twenty-nine volunteers, all male except for one, were recruited in two cohorts. Participants were 50–67% Black, 20–36% Mixed Race and 13–14% White; 40–60% were Hispanic. Age ranged from 21 years to 44 years (mean 32 years), and BMI ranged from 18.7 kg m^−2^ to 32.0 kg m^−2^ (mean 24.3 kg m^−2^). On average, participants smoked 2.9 g of cannabis per day, 6.9 d per week. Severity of CUD was diagnosed as mild (34.5%), moderate (44.8%) and severe (20.7%) across participants.

Two doses of AEF0117 (0.06 mg d^−1^ and 1 mg d^−1^) were tested in escalating order in two cohorts (*n* = 13 each; Fig. 2 and Table 1). Each cohort was divided into two groups (of 6–8 participants) receiving AEF0117 and placebo in randomized order with a minimum 14-d washout period in between the two treatments. The doses of AEF0117 selected were based on a population PK model developed using data from the SAD and MAD studies (Methods). The objective was to obtain, in 90% of participants, plasma concentrations of AEF0117 corresponding to exposures observed in animals at the two dose ranges (1.5 µg kg^–1^ and 15 µg kg^–1^) shown to modify distinct behavioral effects of THC (Supplementary Table 2).

The main objectives of the study were to evaluate the effects of AEF0117 on the perceived ‘good effect’ of cannabis as a measure of abuse liability and on self-administration. An additional objective was to determine if AEF0117 reversed cannabis’ effects on cognitive performance, pain threshold and heart rate. However, cannabis did not produce significant cognitive or analgesic effects relative to baseline in the present study design, so it was not possible to determine whether AEF0117 reversed these effects. In addition, because of institutional regulations regarding data privacy, we were not able to collect heart rate data as originally planned.

Participants, in groups of 3−4, completed two 5-d inpatient periods (first period ‘A’ and second period ‘B’) separated by a ≥ 14-d outpatient washout. During the two testing periods, participants received AEF0117 or matching placebo in counterbalanced order (two dosing sequences: AEF0117 first or placebo first). Participants took capsules at 9:00 each day and then smoked a controlled amount of cannabis (approximately 67 mg of THC smoked over 6 min) 3.5 h later (12:30). Ratings of the subjective effects of cannabis were done five times after cannabis administration (20 min, 40 min, 60 min, 90 min and 120 min after cannabis) using a VAS (0–100 mm). From day 2 to day 5, beginning 5.5 h after AEF0117 administration, participants had four opportunities (at 14:30, 16:30, 18:30 and 20:30) to self-administer cannabis by purchasing individual cannabis puffs using a portion of their study stipend (maximum of six puffs per timepoint, $2 per puff). Participants were told that the cannabis strength could vary from day to day and between participants but that the cannabis they each received at 12:30 that day was the strength available for self-administration that day.

The primary endpoint measuring the good subjective effect of cannabis related to addiction was an ‘Intoxication’ subscale based on a cluster analysis of the 44-item VAS^22^, comprising the arithmetic mean of two items: ‘I feel a Good Effect’ and ‘I feel High’^22^. The positive subjective effects of cannabis were further assessed by individual items on the Cannabis Rating Form (CRF)^23^, where participants rated the cannabis that they had most recently smoked in terms of ‘Cannabis Cigarette Liking’ and ‘Felt Good Cannabis Effect’ (key secondary endpoints) using a VAS. Cannabis self-administration was evaluated by measuring the number of puffs purchased at each timepoint (key secondary endpoint).

The statistical analysis plan (SAP) specified a crossover mixed model repeated measures (MMRM) analysis, which took into account multiple comparisons, using the restricted maximum likelihood estimator (REML) as estimation method and structured covariance matrix with compound symmetry and included all data from those completing (*n* = 13 per dose) both treatment periods (first period ‘A’ and second period ‘B’). However, a significant interaction between the dosing sequence (AEF0117 first or placebo first) and treatment was observed for all primary and key secondary endpoints, indicating that the order of placebo and AEF0117 administration impacted outcome. In this situation, the SAP specified that a parallel group MMRM analysis was to be done with data from the first dosing period only (period A), comparing participants dosed with AEF0117 (0.06 mg or 1 mg, *n* = 6 per dose) or placebo (*n* = 14).

The global crossover MMRM analyses showed that AEF0117 (1 mg) significantly reduced ratings on the ‘Intoxication’ subscale of the 44-item VAS (Fig. 3a–c and Supplementary Table 19) (peak effect, *P* < 0.006; over time, *P* < 0.005) as well as the ‘Felt Good Cannabis Effect’ item on the CRF (over time, *P* < 0.005). A significant interaction between sequence and treatment over time was observed for all outcomes (‘Intoxication’ subscale, *P* < 0.02; ‘Felt Good Cannabis Effect’, *P* < 0.001 and ‘Cannabis Cigarette Liking’, *P* < 0.05). Consequently, the pre-specified parallel group analysis was performed over time for the first dosing period only (period A; Fig. 3d–f and Supplementary Table 20) and showed a robust attenuation of these effects by AEF0117, with even the lower dose (0.06 mg) producing a significant reduction in cannabis ratings (Treatment × Time: Intoxication, *P* < 0.05; CRF items ‘Felt Good Cannabis Effect’, *P* < 0.005 and ‘Cannabis Cigarette Liking’, *P* < 0.02). The 1-mg dose produced a significantly greater effect than the 0.06-mg dose for the ‘Felt Good Cannabis Effect’ item (Dose × Treatment × Time interaction, *P* < 0.05).

In the global crossover analysis, AEF0117 (1 mg; Fig. 3g) also reduced cannabis self-administration (Treatment effect, *P* < 0.03, and Treatment × Dose × Session interaction, *P* < 0.01). A significant interaction between treatment and sequence was again observed (*P* < 0.01), and the pre-specified parallel group analysis (Fig. 3h) for the first dosing period only (period A) confirmed that the 1-mg dose had a larger effect on self-administration than the 0.06-mg dose (Dose × Treatment × Session, *P* < 0.05). Self-administration data were also analyzed by comparing the number of individuals choosing to self-administer cannabis as a function of AEF0117 dose. Although there was a trend for a lower proportion of participants to self-administer cannabis when receiving AEF0117, no significant effect of Treatment or Treatment × Dose interaction (McNemar’s test) was observed.

AEF0117 seemed to reduce the subjective effects of cannabis at lower doses (0.06 mg) than cannabis self-administration, for which 1 mg was needed (Fig. 3). This observation is consistent with the dose range observed in animals in which 15 µg kg^−1^ of AEF0117 (corresponding to 1 mg in humans) was needed to reduce self-administration, whereas 1.5 µg kg^−1^ (corresponding to 0.06 mg in humans) was sufficient to inhibit other THC-induced behaviors (Supplementary Table 2).

To characterize and illustrate the sequence effect, an exploratory post hoc analysis was performed on the two sequences separately. This analysis, portrayed in Fig. 3i for ‘Felt Good Cannabis Effect’ at the most effective dose (1 mg), shows that, when placebo was administered in the first dosing period, AEF0117 decreased ratings of cannabis in the second dosing period. However, when AEF0117 was administered in the first dosing period, there was no significant difference from placebo in the second dosing period. This suggests that AEF0117 maintains its effects even after ≥14 d of washout. These lasting effects likely reflect the long elimination half-life of AEF0117. When AEF0117 was administered during the first period, detectable plasma concentrations of AEF0117 were observed after ≥14 d of washout (Fig. 3j,k). To further investigate the PK characteristics of AEF0117, we did population PK modeling and Monte Carlo simulations, which showed that (1) the trough concentration of AEF0117 reaches steady state after 4 weeks and (2) after 3 months of AEF0117 (1 mg) administration, more than 2 months are needed for drug concentrations to go below detection limits (0.01 ng ml^−1^).

In research volunteers with CUD (*n* = 29), AEF0117 (0.06 mg kg^−1^ and 1 mg kg^−1^) was also safe and well tolerated with no treatment-related SAEs. Among the limited number of TEAEs, the incidence, severity and relatedness to treatment were similar whether AEF0117 or placebo was administered, except for one severe, unrelated AE (dermal cyst) observed in the 1-mg cohort (Table 2). One participant, with a history of auditory and visual hallucinations (not disclosed during screening), experienced two episodes of mild auditory hallucinations during days 1 and 2 of AEF0117 (1 mg) administration. The participant continued the study without any further episodes of hallucination.

**Fig. 4:**
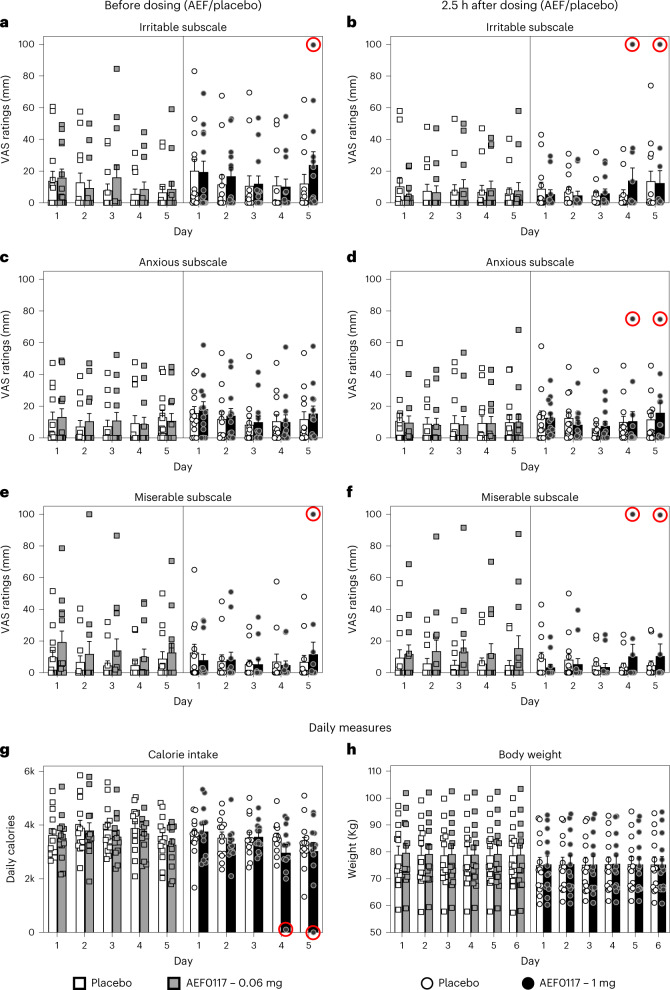
Effects of AEF0117 on mood, food intake and body weight in the phase 2a study. Subjective ratings before dosing with AEF0117 at 9:00 (**a,c,e**) and then 2.5 h after AEF0117 administration, before cannabis smoking (**b,d,f**) for the subscale of the 44-item VAS used to measure precipitated cannabis withdrawal Irritable (**a**,**b**), Anxious (**c**,**d**) and Miserable (**e**,**f**). In the MMRM analysis performed for all three subscales, a small but significant effect was found for the ‘Irritable’ subscale (Treatment × Day × Time interaction, *P* = 0.0373). No significant changes were found for the other two endpoints used to measure precipitated withdrawal: daily caloric intake (**g**) and daily body weight (**h**). For body weight, day 6 is the day of discharge 24 h after the last administration of AEF0117 at day 5 (*n* = 13 per dose cohort). Red circles indicate data from the same participant (included in the mean value calculations and statistical MMRM analysis). Data are represented as mean + s.e.m.

**Extended Data Fig. 7:**
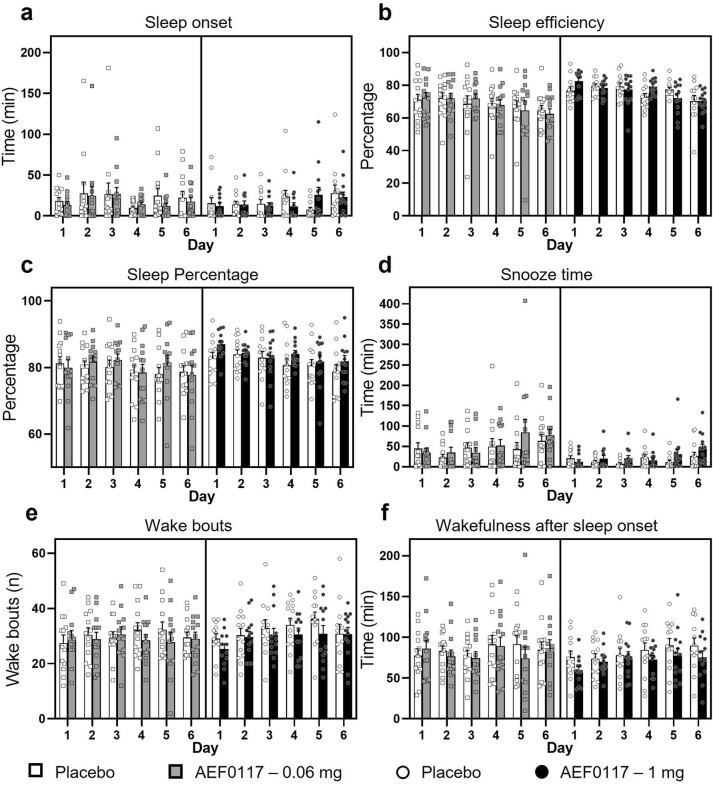
Objective sleep measures with the Actiwatch®. (**a**) Sleep onset. (**b**) Sleep efficiency. (**c**) Sleep Percentage. (**d**) Snooze time. (**e**) Wake bouts. (**f**) Wakefulness after sleep onset. The performed MMRM analysis for all parameters showed a statistically significant overall Treatment effect (no significant interaction with Dose or Days) for Snooze time (Treatment, P = 0.0071) and number of Wake bouts (Treatment, P = 0.0237). Relative to placebo, AEF0117 increased Snooze time and decreased the number of Wake bouts. Day 1 is the morning before the first AEF0117 dose and Day 6 is 24 h after the last AEF0117 dose on Day 5. n = 13 per dose cohort. Data are represented as mean + s.e.m.

**Extended Data Fig. 8:**
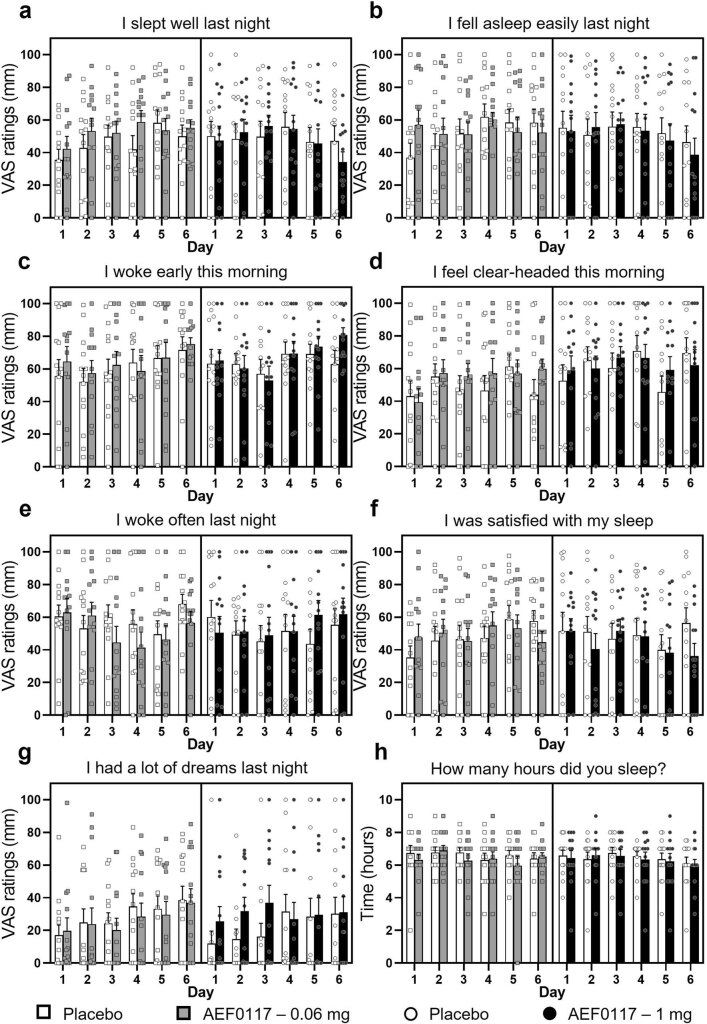
Subjective sleep ratings measured by items of the sleep questionnaire using Visual Analog Scales (VAS) in the Phase 2a study. (**a)** I slept well last night. (**b**) I fell asleep easily last night. (**c**) I woke up early this morning. (**d**) I feel clear-headed this morning. (**e**) I woke often last night. (**f**) I was satisfied with my sleep last night. (**g**) I had a lot of dreams last night. (**h**) How many hours did you sleep last night? The only statistically significant effect was a Dose*Treatment interaction for the ‘I woke often last night’ item. The two doses of AEF0117 had opposite effects on this parameter: 0.06 mg decreasing and 1 mg increasing ratings of waking often; neither AEF0117 dose differed significantly from placebo for this rating. Day 1 is the morning before the first AEF0117 dose and Day 6 is 24 h after the last dosing on Day 5. n = 13 per dose cohort. Data are represented as mean + s.e.m.

We also evaluated whether AEF0117 precipitated symptoms of cannabis withdrawal using daily assessments of food intake, body weight, sleep and mood, measured using the ‘Miserable’, ‘Anxious’ and ‘Irritable’ subscales of the 44-item VAS^22^. Mood was assessed before and 2.5 h after AEF0117 administration, before cannabis administration. Overall, there was little to suggest that AEF0117 precipitated cannabis withdrawal (Fig. 4). AEF0117 did not produce anorexia (Fig. 4g,h) or sleep disruption (Extended Data Figs. 7 and 8) relative to placebo. There was a small but significant effect of treatment (Treatment × Day × Time, *P* < 0.05) for the ‘Irritable’ subscale. As can be seen in Fig. 4a,b, this effect occurred on the last 2 d of treatment and seemed to reflect data from one participant (Fig. 4; red circles) receiving 1 mg of AEF0117. For this individual, ratings were low on the first several days of AEF0117 administration but increased on days 4 and 5 for all mood subscales. This pattern parallels the timecourse of spontaneous withdrawal, where mood symptoms peak after several days of abstinence^24^. Precipitated withdrawal, in contrast, is characterized by abrupt and robust changes in mood within hours of an antagonist administration—for example, naloxone administration to opioid-dependent individuals^25^. To evaluate the contribution of this individual to the significant effect observed, we did an exploratory analysis excluding his data, and there was no longer a significant treatment effect. We hypothesize that this individual was particularly sensitive to AEF0117ʼs inhibition of cannabis effects, and, thereby, he exhibited symptoms of spontaneous cannabis withdrawal despite receiving active cannabis each day.

**Extended Data Fig. 9:**
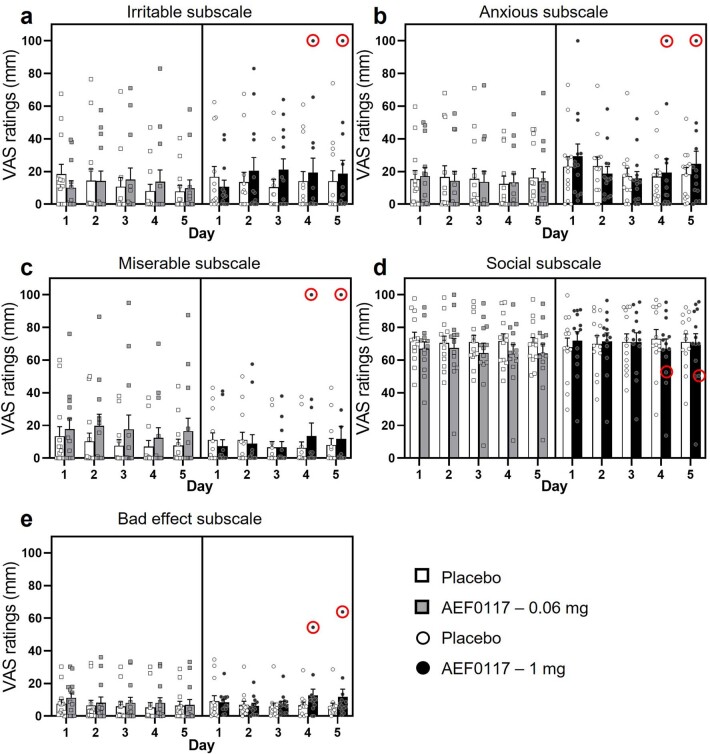
Effects of AEF0117 on mood after cannabis smoking in the Phase 2a study. The subscales of the 44-item VAS used to measure precipitated cannabis withdrawal: (**a**) Irritable, (**b**) Anxious, and (**c**) Miserable were also used to measure mood alterations after smoking cannabis. In this context, in the MMRM analysis performed for all 3 subscales a small but significant effect was found for the ‘Irritable’ subscale (Treatment*Day interaction, P = 0.0418). Small but statistically significant changes were also found in the MMRM analysis for some of the other subscales used to measure mood effects of AEF0117 during cannabis smoking: (**d**) a decrease for the ‘Social’ subscale (Treatment*Day interaction, P = 0.0215) and (**e**) an increase for the ‘Bad effect’ subscale (Dose*Treatment*Day interaction P = 0.0388). n = 13 per dose cohort. Data are represented as mean + s.e.m. Red-encircled dots represent data from one participant, the same individual highlighted by red encircled dots in Fig. 4.

The ‘Miserable’, ‘Anxious’, ‘Irritable’, ‘Tired’, ‘Confused’, ‘Social’ and ‘Bad effect’ subscales of the 44-item VAS^22^ were also used to evaluate potential negative mood effects of AEF0117 after cannabis administration. There were small but significant effects of treatment for the ‘Irritable’ and ‘Bad effect’ subscales (Extended Data Fig. 9a,e). These do not appear to reflect the effects of AEF0117 in combination with cannabis, as ratings of ‘Irritable’, for example, were similarly elevated before cannabis administration (Fig. 4a,b) and seemed driven by the same individual who showed signs of spontaneous withdrawal at the 1-mg dose (red encircled dots). In an exploratory analysis, no significant effects of AEF0117 (1 mg) were found when this individual was excluded from the analysis. There was also a small (<8-mm difference between AEF0117 and placebo in a 100-mm scale; Supplementary Table 21) but statistically significant decrease in ratings on the ‘Social’ subscale (Extended Data Fig. 9d).

Overall, the absence of effects of AEF0117 on food intake or sleep (robust measures of cannabis withdrawal) and the small amplitude changes observed on certain mood ratings do not suggest that AEF0117 precipitates cannabis withdrawal or produces clinically relevant changes in mood among volunteers smoking cannabis.

**Extended Data Fig. 10:**
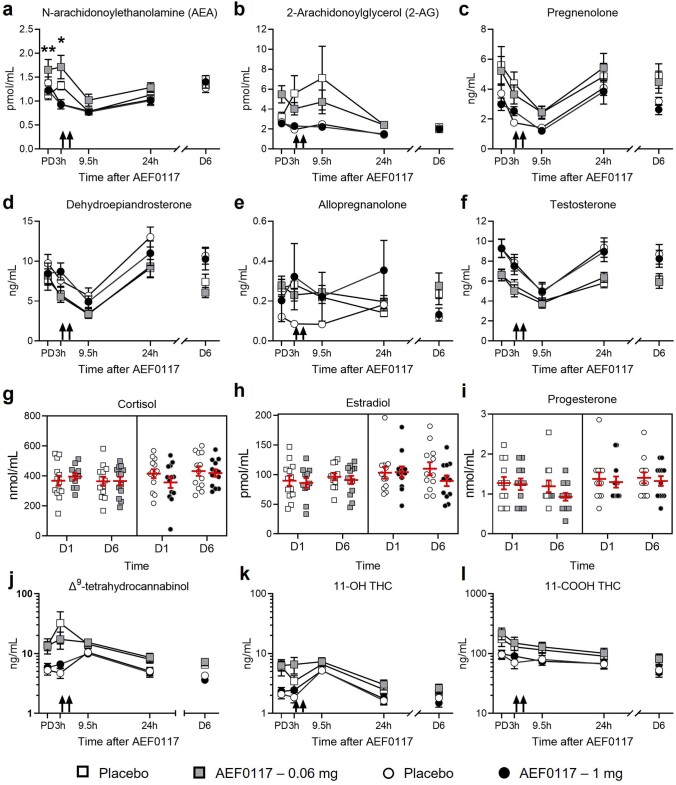
Effects of AEF0117 on endocannabinoids (AEA and 2-AG), pregnenolone, pregnenolone’s downstream steroids, THC and its metabolites in volunteers with cannabis use disorder (CUD) in the Phase 2a study. (**a**) N-arachidonoylethanolamine (Anandamide, AEA). (**b**) 2-Arachidonoylglicerol (2-AG). (**c**) Pregnenolone. (**d**) Dehydroepiandrosterone (DHEA). (**e**) Allopregnanolone. (**f**) Testosterone. (**g**) Cortisol (**h**) Estradiol (only males). (**i**) Progesterone (only males). (**j**) Δ^9^-tetrahydrocannabinol (THC). (**k**) 11-OH THC. (**l**) 11-COOH THC. A MMRM analysis for each dose cohort (n = 13) was performed. (**a**) For AEA the MMRM overall crossover analysis identified a significant Dose*Treatment*Time interaction (P = 0.0015). When the analysis was performed for each dose cohort, a significant Treatment effect was found for the 0.06 dose only (P = 0.0063). This effect seemed to be due to higher basal AEA levels (PD) before AEF0117 dosing (P = 0.0079) that remained high 3 h post dosing (P = 0.0397), suggesting the effect was not caused by AEF0117 administration. The MMRM global crossover analysis also identified significant Dose*Treatment*Time interactions for: (**d**) Testosterone, P = 0.0012 and (**e**) Allopregnanolone, P = 0.0009. However, when these analyses were performed for each dose cohort independently, no significant effect of Treatment or Treatment*Time interaction was found. Other MMRM analysis did not revealed any significant effects of Treatment or Treatment*Time. Data are represented as mean ± s.e.m. of plasma concentrations. PD = pre-dose levels obtained 30 min before AEF0117 administration. Black arrows indicate cannabis smoking at 3.5 and 5.5 h after AEF0117 dosing. * P = 0.0397 and ** P = 0.0079 AEF0117 0.06 mg compared to placebo.

Consistent with animals and healthy volunteers, endocannabinoids (2AG and AEA), pregnenolone and its downstream steroids did not increase during AEF0117 administration compared to placebo in research volunteers with CUD (Extended Data Fig. 10), except that AEA levels were significantly higher with the 0.06-mg AEF0117 dose relative to placebo (Extended Data Fig. 10a). However, participants in this cohort had high basal AEA levels (before medication administration), and levels remained high 3 h after dosing (*P* < 0.05), suggesting that the effect was not caused by AEF0117 administration. Finally, no statistically significant differences were observed in plasma THC and its metabolites (11-COOH-THC and 11-OH-THC) between the periods of AEF0117 and placebo dosing (Extended Data Fig. 10j–l).

## Discussion

AEF0117 is the first of a new pharmacological class, the CB_1_-SSi, with an MOA that has never before, to our knowledge, been investigated in humans. We chose the name SSi to purposely differentiate this new pharmacological class from known inhibitors: orthosteric antagonists and prototypical negative allosteric modulators (NAMs). These drugs act mainly by blocking (antagonists) or decreasing (NAMs) the access of ligands to the receptor^9^. Such MOAs modify all receptor activity and, thus, can impair normal physiological function and produce serious adverse effects. As a result, few antagonists or NAMs have been approved to treat brain diseases. CB_1_-SSi binds to an allosteric binding site but has a distinctive MOA. CB_1_-SSi does not modify orthosteric ligand binding but, rather, restricts the conformational changes that an agonist can induce in the CB_1_, thereby inhibiting only some of its cellular activity. CB1-SSi can, thus, be considered a subclass of biased allosteric modulators^26^. By this mechanism, AEF0117 potently inhibits the effects of the receptor ligand, THC, without altering behavior per se in animals or humans. CB1-SSi appears to be one of the few classes of compounds able to inhibit the effects of a receptor agonist without having psychoactive effects per se, which provides a considerable advantage for its potential therapeutic use and constitutes a major advance in the pharmacology of inhibitors.

The unique pharmacological profile of AEF0117 is also illustrated by its ability to decrease self-administration, addiction-related subjective effects and the unconditioned effects of cannabis and THC without precipitating withdrawal. By contrast, CB_1_ agonists may decrease cannabinoid self-administration but do not inhibit THC’s unconditioned effects, whereas CB_1_ antagonists precipitate withdrawal, decrease the unconditioned and subjective effects of THC and typically result in compensatory increases in self-administration. One possible explanation for the unique action of AEF0117 on THC’s effects is that AEF0117, by its signaling-specific inhibition, transforms the effects of THC into a biased CB_1_ agonist, resulting in reduced addiction-related effects.

Although the current findings establish the signaling-specific effects of AEF0117 and its unique pharmacological profile, future studies are needed to identify its full MOA. AEF0117 could potentially modify other important CB_1_-activated signaling pathways beyond MAPK, such as Go-mediated inhibition of voltage-operated calcium channels. It is also possible that AEF0117 has ligand specificity and interferes less with endocannabinoid-mediated than with THC-mediated CB_1_ activation. This hypothesis is supported by the minimal effects of repeated AEF0117 administration per se on spontaneous behavior and on measures of anxiety, sucrose preference and food intake. Further study is needed to test the ligand specificity of AEF0117 under conditions of increased endocannabinoid availability, as occurs after administration of MAGL and FAAH inhibitors, for example.

The long half-life of AEF0117 seems to be caused by a combination of at least two factors: (1) slow clearance: AEF0117 is lipophilic so it is distributed to adipose tissue and then slowly cleared; (2) metabolic stability: AEF0117 does not modify the activity of, nor is it notably metabolized by, any major drug metabolic pathways (CYP and phase II enzymes), and no metabolite accounting for more than 1% of the parent compound has been identified. Although compounds with a long half-life could raise safety concerns, this does not seem to be the case for AEF0117. The simulation performed with the population PK model showed that, after 3 months of treatment with AEF0117 (1 mg d^−1^), the simulated median plasma concentration was similar to that observed in the MAD study after 2 mg d^−1^ for 7 d—that is, lower by a factor of 3 than those after 6 mg d^−1^ for 7 d, a dose that was still safe and well tolerated. Furthermore, the simulated concentrations were well below (320 times lower) the exposure observed at the NOAEL found in the 3-month preclinical repeated toxicology studies. Rather than causing a safety concern, the long half-life of AEF0117 may actually be a beneficial feature of a medication developed to treat CUD by reducing potential issues with medication compliance.

One factor that likely contributed to the successful and rapid development of AEF0117 is the innovative selection process used. The major causes of attrition in drug development are (1) lack of therapeutic efficacy, (2) toxicity and (3) formulation and bioavailability issues, each accounting for approximately one-third of the global attrition rate^27^. Usually, drug candidates are selected for their potency and efficacy. Toxicity, formulation and bioavailability are studied only later in development, resulting in only about 4% of developed compounds achieving approval^28^. By using in vitro toxicity, formulation and bioavailability as the first criteria of selection, we were able to reduce the impact of two of the three primary causes of attrition early on and could then dedicate considerable resources for an extensive pharmacodynamic characterization of a small number of compounds that had a higher chance of achieving and succeeding in phase 2 studies than by using the classic approach.

In conclusion, AEF0117 is the first of a new pharmacological class of inhibitors, CB_1_-SSi, that modify the activity of their target receptor in a signaling-specific manner. Because these drugs reproduce the effects of a natural mechanism to counteract CB_1_ overactivation^11^, they can inhibit the effects of THC without altering the basal activity of the CB_1_. Therefore, these compounds seem to have no effect on normal behavior and physiological activity while decreasing cannabis’ abuse-related and reinforcing effects, resulting in a well-tolerated and potentially efficacious therapy for CUD.

## Methods

### Laboratory animals

#### Non-GLP experiments

Rodents (rats and mice) used to study the effects of AEF0117 and rimonabant on THC-mediated responses and for PK studies were individually housed in temperature-controlled (22 °C) and humidity-controlled (60%) facilities under a constant light/dark cycle (lights on, 8:00–20:00, except for self-administration studies: lights on 20:00–8:00). Food and water were freely available except for food intake studies and for WIN55,212-2 self-administration in mice. After arrival, the mice and rats were handled periodically for 2 weeks before experiments. Rodents were purchased from Janvier Labs, Charles River Laboratories or IFFA CREDO. All experiments were conducted in strict compliance with the recommendations of the European Union (2010/63/EU) and approved by the respective ethics committees: French Ministry of Agriculture and Fisheries (authorizations nos. 3310035, 3309004 and 3312059); Ethical Committee of the University of Bordeaux; Ethical Committee for Animal Research (CEEA-PRBB), University Pompeu Fabra; and Oncodesign Internal Ethical Committee. Except if specified elsewhere, the following strains of adult male or female rodents were used: Sprague Dawley rats (RRID: RGD_734476, weighing 200–380 g depending on the experiments); C57BL/6J mice (RRID: MGI:5752053, weighing 23–25 g); C57BL/6N mice (RRID: MGI:6236253, weighing 22–24 g); and CD-1 Swiss mice (RjORL: SWISS, weighing 25–43 g).

Male non-human primate/squirrel monkeys (*Saimiri sciureus*), weighing 800–1,100 g, used for self-administration experiments, were from an in-house colony (Intramural Research Program, National Institute on Drug Abuse (NIDA), National Institutes of Health (NIH), originally from the NIH Animal Center). The monkeys were housed one per cage in a two-tier rack with six compartments (Environ-Richment 6-Pack, Britz & Company; dimensions of the inside compartment: 17 1/2-ft width × 27 1/8-ft diameter × 30-ft height; floor space: 3.2 ft^2^ per compartment) under a 12-h light/dark cycle. Controlled temperature (21–23.5 °C) and humidity (35–55%) were provided in the housing facility and test rooms. The monkeys were acclimatized to the animal housing room for a period of at least 12 months. They were fed a daily food ration consisting of five biscuits of high-protein monkey diet (Lab Diet 5045, PMI Nutrition International) and two pieces of Banana Softies (Bio-Serv) that maintained their body weight at a constant level throughout the study. Fresh fruits, vegetables and environmental enrichment were provided daily. The animals had free access to filtered tap water. The experiments were performed in accordance with the Guide for the Care and Use of Laboratory Animals (8th edition) and guidelines of the Institutional Animal Care and Use Committee of the Intramural Research Program, NIDA, NIH, Department of Health and Human Services (DHHS). The monkeys were maintained in facilities fully accredited by the Association for Assessment and Accreditation of Laboratory Animal Care (AAALAC).

#### GLP experiments

Rats and dogs used for the toxicology and safety pharmacology experiments conducted using GLP conditions were housed in accordance with the guidelines of Directive 2010/63/UE of the European Parliament and of the council of 22 September 2010 for the protection of animals used for scientific purposes. Male and female Sprague Dawley SPF (specific pathogen free) rats aged 6–8 weeks (from Charles River Laboratories) and male and female Beagle dogs aged 8–10 months (weighing 6–12 kg) (from Marshall BioResources) were used. Rats were housed (separated by sex) in standard-sized cages with sawdust (or equivalent) bedding and had ad libitum access to food (RM1 (E)-SQC SDS/DIETEX) and drinking water. In the 28-d study, dogs were individually housed in standard-sized pens (2.25 m^2^) and received a daily ration of 300 g of food (SDS/DIETEX D3(E) SQC) with 1,500 ml of drinking water. In the 91-d study, dogs were housed collectively by group and by sex in standard-sized pens and received a daily ration of 230 g of food (ssniff Hd Ereich Extrudate V3286 SQC) with ad libitum access to water. The rats and dogs were housed in an air-conditioned (20–24 °C) animal house kept at a relative humidity between 45% and 65%.

### Drugs

Δ^9^-tetrahydrocannabinol (THC) for the rodent studies was purchased as dronabinol resinous oil (THC-1295S-250) or 10 mg ml^−1^ solution in 100% ethanol (THC-LOO657-E-1010) (THC Pharm GMBH–The Health Concept). The resin was dissolved at 50 mg ml^−1^ (w/v) in 100% ethanol. For injection, ethanol solutions were solubilized in 0.9% NaCl containing ethanol (2%) and Tween 80 (2%). For the food intake, locomotion and PPI experiments, THC was solubilized in 0.9% NaCl containing ethanol (4%) and Cremophor (4%).

For the self-administration experiments with monkeys, THC in ethanol solution (49.9 ± 0.027 mg ml^−1^) was provided by RTI International (RTI log no.: 13475-1212-186). Stock solution (0.4 mg ml^−1^) was prepared by dissolving THC in a vehicle containing 1% ethanol, 1% Tween 80 and saline. The stock solution was further diluted with saline as needed.

Rimonabant (SR141716A, Cayman Chemical, reference: 90000484) was dissolved in DMSO (2%) and Tween 80 (2%) in injectable NaCl 0.9% solution.

When THC and rimonabant were administered through the intraperitoneal (i.p.) route, the injection volumes were 1 ml kg^−1^ of body weight for rats and 10 ml kg^−1^ for mice.

WIN55,212-2 (Sigma Chemical Co.), used for the mice self-administration experiments, was dissolved in one drop of Tween 80 and diluted in saline solution.

Ketamine hydrochloride (100 mg kg^−1^) and xylazine hydrochloride (20 mg kg^−1^) were mixed and dissolved in ethanol (5%) and distilled water (95%). This anesthetic mixture was administered i.p. before catheter implantation in an injection volume of 20 ml kg^−1^ of body weight. Thiopental sodium (5 mg ml^−1^) was dissolved in distilled water and delivered by infusion of 0.1 ml through the i.v. catheter.

AEF0117 was provided by Aelis Farma to all research sites. Laboratory-scale batches, made by the contract research organization (CRO) Atlanchim Pharma, were used for selection, proof-of-concept and PK studies. Non-GMP pilot-scale batches, made by the contract development and manufacturing organizations (CDMOs) Roowin and Symeres, were used for toxicology studies. Good Manufacturing Practice (GMP) batches, made by Roowin and Symeres, were used for clinical studies.

### Formulation and dosing of AEF0117

The solubility of two parent compounds was evaluated first in an in vitro screening with 10 surfactants and lipidic excipients. The four excipients (lipidic) providing the best solubility were tested with 12 additional compounds, including AEF0117, and 10 compounds were then compared in vivo in initial PK studies that showed that corn oil was the most appropriate marketable formulation. Consequently, AEF0117 in a corn oil solution was used for animal and human studies. All in vitro solubility screening was performed by the CRO Drugabilis.

### Steroids and cannabinoids quantification

#### Plasma sampling

Blood samples were collected in EDTA or lithium heparin-coated tubes. After centrifugation for 20 min at 500*g* under refrigeration (4 ± 2 °C), the plasma supernatant was divided into two tubes to separately analyze steroids and cannabinoids (endocannabinoids and THC and its metabolites). Samples were snap frozen after removal and kept at −80 °C until analysis.

#### Plasma sample analysis

The steroids pregnenolone (PREG), testosterone (TESTO), allopregnanolone (ALLO), dehydroepiandrosterone (DHEA) and corticosterone (CORT); the cannabinoids N-arachidonoylethanolamine (Anandamide, AEA), 2-arachidonylglycerol (2-AG), Δ^9^-tetrahydrocannabinol (THC); and THC metabolites 11-Nor-9-carboxy-Δ^9^-tetrahydrocannabinol (11-COOH-THC) and 11-hydroxy-Δ^9^-tetrahydrocannabinol (11-OH-THC) were quantified using an isotopic dilution method (with deuterated internal standard analogs) combined with gas chromatography-negative chemical ionization–tandem mass spectrometry (GC-NCI–MS/MS) for steroids analysis or liquid chromatography-positive chemical ionization–tandem mass spectrometry (LC-APCI–MS/MS) for cannabinoids analysis. Bioanalysis of endocannabinoids and steroids by MS were performed at the Neurocentre Magendie of INSERM.

Progesterone, cortisol and estradiol (E2) assays were performed by LabCorp Test Master (using an electrochemiluminescence immunoassay (ECLIA); tests 004515 and 004317 for progesterone and cortisol, respectively, and the LC–MS/MS method for E2).

#### Extraction and purification of steroids and cannabinoids for MS analysis

Analysis of the compounds of interest required preliminary steps to allow pre-concentration and reduction of biological matrices.

For steroids, plasma was first spiked with deuterated analog internal standards (PREG-d4, TESTO-d3, ALLO-d4, DHEA-d3 and CORT-d8) and then mixed with methanol/H_2_O (75/25, v/v) for homogenization. Dried extracts were diluted with methanol/H_2_O (5/95, v/v), and then steroids were extracted by a simple solid-phase extraction (SPE) method using reverse-phase C18 columns (Agilent) and methanol as elution solvent, as described previously^11^_._

For cannabinoids, a homogenous solution of plasma (qsp 1 ml with Tris-HCl 50 mM pH 7.5 if needed)/methanol/chloroform (1:1:2) was spiked with deuterated analog internal standards (AEA-d4, 2-AG-d5, THC-d3, 11-COOH-THC-d3 and 11-OH-THC-d3). Chloroform was added to perform liquid–liquid extraction (LLE). This step was repeated two times. The dried lipid extract was diluted with methanol/H_2_O (30/70, v/v) and purified by SPE using cyclohexane/ethyl acetate (1:1) as elution solvent^29^.

After elution, steroid or cannabinoid extracts were concentrated under a gentle nitrogen stream evaporation.

#### Derivatization of steroid extracts

Dried methanol extracts of plasma samples were subjected to deconjugation and derivatization steps to release the free analytes and to increase volatility, heat resistance and ionizability. The formation of pentafluorobenzyl oximes for NCI detection was followed by trimethylsilyl ether formation for adequate sensitivity and selectivity for GC–MS/MS analysis.

#### Quality control and calibration curves

All analyses were conducted in compliance with GLP-like procedures (but not GLP) according to qualified assay methods for steroids and cannabinoids, including selectivity, sensitivity, accuracy, between-run and within-run precision and recovery. In addition, an assay of the stability of the internal standards in each run of analysis was performed to ensure that the amount of non-deuterated steroids and cannabinoids was always less than 0.3%. A calibration standard curve was implemented in each batch of analyzed samples by spiking deuterated internal standards with increasing amounts of reference standards through 10 calibration levels (CCT0 to CCT9) to calibration samples, and the extraction procedure was performed as described above. The response was linear (R^2^ > 0.990) for each analyte. To evaluate between-run precision and reproducibility, quality control samples were run for each batch of samples analyzed.

#### MS quantification

The derivatized steroid samples were injected (1 μl) directly into a GC–MS/MS XLS Ultra Thermo mass spectrometer (Thermo Finnigan) via an AS3000 II autosampler. The instrument was employed in negative ion chemical ionization mode and a 15-m Rtx-5Sil MS W/Integra Guard capillary column (Restek) with a 0.25-mm inside diameter, and 0.1-μm film thickness was employed for analyte resolution. Data were acquired using Thermo Xcalibur Access (Thermo Fisher Scientific).

Mass spectral analyses of cannabinoids were performed on a liquid chromatography-atmospheric pressure chemical ionization–tandem mass spectrometry (LC-APCI–MS/MS) device operating in positive ion mode. The TSQ Quantum Access triple-quadrupole instrument was used in conjunction with a Surveyor LC Pump Plus (Supelco C18 Discovery analytical column) and cooled autosampler. Data were acquired using Thermo Xcalibur version 2.0.7 (Thermo Fisher Scientific).

For quantification, the mass spectrometers were operated in selected reaction monitoring (SRM) mode to enhance sensitivity, and the concentration of each compound was calculated by linear regression of the peak area corresponding to the diagnostic fragment ion (*m*/*z*) with the highest intensity.

For each analyte, the isotope dilution method was used to achieve accurate quantification using the respective calibration curve. Individual plasma concentrations were expressed as ng ml^−1^ for steroids and THC and its metabolites and as pmol ml^−1^ for endocannabinoids.

### AEF0117 quantification

Plasma and brain AEF0117 concentrations were measured using LC–MS/MS. AEF0117 was derivatized by the addition of hydroxylamine during the extraction process.

The CRO Oncodesign performed the animal sample quantification, and the CRO Biotrial Bioanalytical Services performed the human sample quantification. Methods used for toxicokinetics (TK) analysis in animals and for the human samples were validated according to the applicable principles of GLP. System control and data collection were done using Analyst software version 1.5 or version 1.6 (AB Sciex). PK parameters were determined using WinNonLin version 6.3 (Certara).

### In vitro characteristics of AEF0117

#### Effect of AEF0117 on in vitro radioligand binding assay

HEK293 cells (American Type Culture Collection (ATCC), CRL-1573, RRID: CVCL_0045) were stably transfected with human CB_1_ N-terminally tagged with bovine pre-prolactin signal sequence and 3-hemagglutinin residues as previously described^30^. Cells were cultured in DMEM + 10% FBS under zeocin-resistant (250 μg ml^−1^) antibiotic selection. Cells were expanded into approximately 24 × 175-cm^2^ vented-cap plastic culture flasks and, when confluent, dislodged using ice-cold 5 mM EDTA. Cells were sedimented and snap frozen at −80 °C. The pellet was resuspended in ice-cold sucrose buffer (200 mM sucrose, 50 mM Tris-HCl pH 7.4, 5 mM MgCl_2_, 2.5 mM EDTA) and manually homogenized using a glass pestle and dounce homogenizer. The homogenate was centrifuged at 1,000*g* for 10 min. The membrane-rich supernatant was retained and re-centrifuged at 26,916*g* for 30 min. The membrane pellet was resuspended in sucrose buffer, and protein levels were quantified using a Bradford protein assay kit and stored at −80 °C.

Competition binding assays were conducted on purified membrane preparations as described previously^30^. Concentration dilution series of AEF0117 (0.1 nM, 1 nM, 0.01 μM, 0.1 μM, 1 μM and 10 μM, plus vehicle) were prepared in binding buffer (50 mM HEPES pH 7.4, 1 mM MgCl_2_, 1 mM CaCl_2_, 2 mg ml^−1^ BSA). [^3^H]CP55,940 (PerkinElmer) was also diluted to a final concentration of 1.6 nM in binding buffer. Membranes were similarly diluted to 5 μg per assay point. Reagents were mixed at a final assay volume of 200 μl in V-bottom polypropylene 96-well plates and incubated for 1 h at 30 °C. Simultaneously, 1.2-μm pore fiberglass filters of a 96-well harvest plate (PerkinElmer) were blocked with 0.1% w/v branched polyethylenimine. After incubation, the harvest plate was applied to a vacuum manifold at 5 mmHg. Wells were washed with 200 μl of ice-cold wash buffer (50 mM HEPES pH 7.4, 500 mM NaCl, 1 mg ml^−1^ BSA). Drug and membrane were transferred from the V-bottom mixing plate and applied to the harvest plate. Wells of the V-bottom plate were washed with 200 μl of ice-cold wash buffer, and the wash was also applied to the respective wells on the harvest plate. Finally, each well on the harvest plate was washed three times with 200 μl of ice-cold wash buffer. The harvest plate was then allowed to dry overnight. The underside of the harvest plate was sealed, and 50 μl of Irgasafe Plus (PerkinElmer) was applied to each well and read in a Wallac MicroBeta TriLux liquid scintillation counter for 2 min per well. Counts were acquired using a MicroBeta^2^ Windows Workstation, version 2.2.0.19 (PerkinElmer). The Trilux scintillation counter has three detectors. For the results among the detectors to be equivalent, data are normalized for small variations in efficiency and background detection among the detectors. Data measured in counts per million (CPM) are corrected by dividing by this efficiency coefficient and reported as corrected CPM (CCPM). Corrected counts were exported and analyzed in GraphPad Prism. The specific binding window for each of *n* = 3 independent experiments was normalized to radioligand binding in the presence of vehicle only (100%) or displacement caused by 10 µM THC (0%).

#### Effect of AEF0117 on THC-induced decreases in cellular respiration

The aim of this study was to test the effect of AEF0117 on the inhibition of cellular respiration induced by THC (1 µM) in HEK293 cells transiently transfected, using polyethylenimine (PolySciences), with the hCB_1_ receptor provided by Ken Mackie (Gill Center for Biomolecular Science, Indiana University).

HEK293 cells (ATCC, CRL-1573, RRID: CVCL_0045, batch 59534772) transiently transfected with the hCB_1_-expressing plasmid were first treated with AEF0117 (dissolved in acetonitrile 0.01%). After 15 min of incubation, THC (1 µM, dissolved in ethanol 0.0034%) or vehicle was added in the culture dishes for 30 min. First, a dose–response experiment with AEF0117 (0 nM, 1 nM, 5 nM, 50 nM and 100 nM) was performed with *n* = 4 per condition. Then, two supplementary experiments were performed to confirm the reversal by AEF0117 (100 nM) of THC inhibition of cellular respiration (*n* = 6 per condition). The effect of AEF0117 on cellular respiration was also studied (*n* = 4 per condition) in HEK293 cells with a mutated receptor that expressed a glycine (G) in position 1.49 (hCB_1_Rp.E1.49G) instead of a glutamate (E); we previously showed^11^ that this mutation invalidates the pregnenolone binding site and suppresses pregnenolone’s effects.

Cellular respiration was measured in a calibrated oxygraph (Oxygraph-2k, Oroboros Instruments) equipped with a Clark electrode and DatLab software. Oxygen consumption (OC) rate was used to measure cellular respiration. The effects of THC, in the absence and in presence of AEF0117, on OC rate were expressed as a percentage of the baseline OC of the cell treated with the AEF0117 vehicle and the THC vehicle in the same experiment.

#### Effect of AEF0117 on THC-induced decreases in cAMP

CHO cells stably expressing the human CB_1_ receptor (CHO-hCB_1_) were used in these experiments (ES-110C, PerkinElmer).

The effects of AEF0117 at four doses (0 nM, 10 nM, 100 nM and 1 μM, dissolved in N,N-dimethylformamide 0.01%) were tested against a dose–response function of THC (0.3 nM, 1 nM, 3 nM, 10 nM, 30 nM, 100 nM and 300 nM, plus vehicle) dissolved in ethanol 0.0063%.

CHO-hCB_1_ cells were treated by concomitantly adding THC and the test compound for 45 min. Forskolin (2.5 µM) was also simultaneously added in all the conditions tested to sustain cAMP basal level. At the end of the treatment, cells were lysed for cAMP quantification. All measures were performed in triplicate in one experiment.

The quantitative determination of cAMP was done using a competitive fluorescence immunoassay. Data were expressed as % of Δ fluorescence (ΔF) that was calculated as follows: ΔF% = (sample fluorescence − negative control fluorescence) / negative control fluorescence. These experiments were done by the CRO Fluofarma.

#### Effect of AEF0117 on THC-induced increase in p-ERK1/2

The aim of this study was to assess the effect of AEF0117 on THC-induced increases in p-ERK1/2 in two different cell lines: CHO-hCB_1_ and STHdh^Q7/Q7^.

CHO-hCB_1_ cells are CHO-K1 cells that stably express hCB_1_ (ES-110-C, PerkinElmer). These cells were plated in 96-well plates (35,000 cells per well) in DMEM-F12 culture medium (11330, Gibco) supplemented with 10% FBS and geneticin and incubated for 24 h at 37 °C under 5% CO_2_ to reach approximately 90% confluence. Then, the cells were deprived of FBS for 4 h in the presence of 0.1% BSA (04-100-812-C, Euromedex), pre-incubated for 30 min with increasing concentrations of AEF0117 (0.1 µM, 0.3 µM, 1 µM, 3 µM and 10 µM, dissolved in DMSO 1%) or its vehicle and then treated for 10 min with THC (30 nM, dissolved in DMSO 0.05%) or its vehicle in DMEM-F12 supplemented with 0.1% BSA.

STHdh^Q7/Q7^ cells are a striatum-derived cell line that endogenously express murine CB_1_ (Coriell, CH00097, RRID: CVCL_M590). These cells were plated in 96-well plates (20,000 cells per well) in DMEM culture medium (61965, Gibco) supplemented with 10% FBS and incubated for 24 h at 33 °C under 5% CO_2_ to reach approximately 90% confluence. After 24 h of FBS deprivation, cells were pre-incubated for 30 min with increasing concentrations of AEF0117 (0.1 µM, 0.3 µM, 1 µM, 3 µM and 10 µM, dissolved in DMSO 1%) or its vehicle and then treated for 30 min with THC (10 µM, dissolved in DMSO 0.05%) or its vehicle in DMEM medium.

At the end of the treatment, both cell lines were lysed with AlphaLISA lysis buffer (100 µl per well), and the activation of the ERK1/2 pathway was evaluated by quantifying phosphorylated-ERK1/2 (p-ERK1/2) levels using AlphaLISA SureFire Ultra p-ERK1/2 (Thr202/Tyr204) assay kit (ALSU-PERK-A10K, PerkinElmer) according to the manufacturerʼs guidelines. The resulting signal was acquired with an EnSpire Alpha plate reader (PerkinElmer) using EnSpire Manager software. Data are expressed as a percentage of the THC effect in the absence of AEF0117. In the case of CHO-hCB_1_ cells, data represent the mean alpha signal obtained in one representative experiment with *n* = 4. For STHdh^Q7/Q7^ cells, data represent the mean alpha signal of four independent experiments, each with at least *n* = 3 replications.

### Administration of AEF0117 in laboratory animals

In laboratory animal studies, unless otherwise specified, AEF0117 was administered by oral gavage in a corn oil solution (Sigma-Aldrich) between 2 ml kg^−1^ and 5 ml kg^−1^ depending on the study.

### PK experiments with AEF0117 in laboratory animals

#### Plasma and brain concentrations of AEF0117 in male and female mice

After administration of AEF0117 (0.3 mg kg^−1^, 4 mg kg^−1^ and 10 mg kg^−1^, orally) to male and female CD-1 Swiss mice (*n* = 3 per sex), blood was withdrawn by cardiac puncture after isoflurane anesthesia at 0.5, 1, 1.5, 2, 4, 5, 6, 8, 10, 24, 36 and 48 h after dosing. Blood samples were centrifuged within 15 min after collection (~7 min at 1,600*g* and 4 °C) to obtain plasma. The brain was also harvested, weighed and frozen on dry ice. Plasma and brains were stored below −70 °C until analysis.

#### Plasma and brain concentrations of AEF0117 in male and female rats

After dosing with AEF0117 (1.6 mg kg^−1^, orally), blood was collected from male and female Sprague Dawley rats (*n* = 3 per sex, 8–9 weeks old) 12 times (0.5, 1, 1.5, 2, 3, 4, 5, 6, 7, 24, 36 and 48 h). Blood samples were obtained from catheterized animals housed in Culex cages with an automated blood sampler—a robotic system designed to facilitate the sampling of whole blood from awake and freely moving rodents. At the last withdrawal timepoint (48 h after dosing), blood aliquots were manually collected from the femoral catheter. This experiment was performed by the CRO Oncodesign.

The blood and brain concentrations of AEF0117 were studied in a separate experiment. After administration of AEF0117 (1.6 mg kg^−1^ by oral gavage), Sprague Dawley male rats (*n* = 3, per timepoint) were anaesthetized by isoflurane anesthesia at 1, 2, 3, 5, 7, 10 and 24 h after dosing. Blood was withdrawn by cardiac puncture, and the brains were harvested. Brains were also collected at 48 h from rats in the first experiment aimed to compare AEF0117 in male and female rats.

In both experiments, blood samples were centrifuged ~7 min at 1,600*g* (4 °C), and the plasma was collected. The brain was weighed and frozen on dry ice. Plasma and brain were stored below −70 °C until analysis.

#### Plasma concentrations of AEF0117 after oral and i.v. administration in dogs

This study involved three male and three female dogs (Beagles, CEDS, 21–36 months old) that received two administrations of AEF0117: the first was orally, and the second was i.v., with 21 d between administrations. For both conditions, animals were fasted overnight before dosing and up to approximately 2 h afterwards. On the day of the oral study, animals were dosed with AEF0117 at 0.7 mg kg^−1^ dissolved in corn oil, and blood samples were collected from the cephalic vein at 0.5, 1, 1.5, 2, 4, 5, 6, 8, 10, 24, 36 and 48 h after dosing. For the i.v. study, AEF0117 was dissolved in 30% 2-hydroxypropyl-β-cyclodextrin in 5% glucose at a concentration of 0.31 mg ml^−1^. AEF0117 was administered at a volume of 2.25 ml kg^−1^ to obtain a final dose of 0.7 mg kg^−1^. Blood samples were drawn from the cephalic or jugular veins at the following timepoints: before dose and then at 0.125 h (7.5 min), 0.25 h, 0.5 h, 1 h, 2 h, 4 h, 6 h, 8 h, 10 h, 24 h and 48 h after dosing.

Blood samples were immediately cooled on ice. Plasma was prepared by centrifugation for 7 min at 1,600*g* under refrigeration (4 ± 2 °C), performed within 15 min at maximum after blood sampling. Plasma was divided into two polypropylene tubes containing at least 500 µl and then stored frozen (≤−70 °C) until assay.

#### Toxicokinetic studies in rats and dogs (GLP conditions)

PK evaluations of AEF0117 administered orally were also performed during the 28-d and 91-d oral toxicity study in male and female rats (Sprague Dawley SPF) and dogs (Beagle). The analysis was performed for all doses of AEF0117 (28-d toxicity: 2, 9 and 36 mg kg^−1^ d^−1^; 91-d toxicity: 2, 20 and 65 mg kg^−1^ d^−1^). For all studies in rats and the 91-d toxicity study in dogs, the timepoints on days 1, 28 and 91 were: before dose and 1, 3, 5, 8 and 24 h after dose. For the 28-d toxicity study in dogs, the timepoints on days 1 and 28 were: before dose and 1, 2, 4, 8, and 24 h after dose. In both species, only mean pre-dose plasma concentrations were calculated on day 14 for the 28-d study and on day 28 for the 91-d study. For the experiments in rats, blood samples for drug analysis were taken from the satellite animals used for the toxicokinetic (TK) analysis (*n* = 3 per sex per timepoint and per group). For experiments performed in dogs, blood samples were drawn from the cephalic or jugular veins of all animals (*n* = 3 per sex per group).

TK analysis was performed separately per sex on mean concentrations by a non-compartmental analysis (NCA). The linearity of exposure was evaluated by comparison of the AUC/dose and C_max_/dose ratios. Possible accumulation was evaluated from the ratio of AUC on day 28 and on day 1 for each dose level and each sex. Samples lower than the limit of quantification (LLQ) values (that is, < 8.00 ng ml^−1^ in rats and <20.0 ng ml^−1^ in dogs) were not included in the calculation of TK parameters. Concentrations of AEF0117 were found below LLQ in all pre-dose samples on day 1.

### Measurement of endocannabinoids and pregnenolone’s downstream steroids after administration of AEF0117 in laboratory animals

#### Experiments in rats (Sprague Dawley SPF)

Blood samples for steroid and endocannabinoid assays were also taken from the satellite animals used for the 28-d repeated oral toxicology study (*n* = 3 per sex per dose per timepoint) testing three doses of AEF0117 (2, 9 and 36 mg kg^−1^ d^−1^). Blood samples were drawn from the jugular vein under isoflurane anesthesia. Timepoints were: day 14 at 5 h and 24 h after dose and day 28 at 24 h after dose. The steroids assayed were: TESTO, DHEA and ALLO. The endocannabinoids assayed were: N-arachidonoylethanolamine (Anandamide, AEA) and 2-arachidonylglycérol (2-AG).

#### Experiments in dogs (Beagles)

Blood samples for steroid and endocannabinoid assays were also taken from the animals (*n* = 3 per sex per dose) used for the 28-d repeated oral toxicology studies that received one of three doses of AEF0117 (2, 9 and 36 m kg^−1^ d^−1^). Timepoints were: day 1 at pre-dose and 1, 2, 4, 8 and 24 h post-dose; day 14: pre-dose; and day 28: pre-dose for treated animals. Vehicle timepoints were day 1 at pre-dose and 2 h and 4 h post-dose and day 14 and day 28 at pre-dose. We used MS to assay the same steroids and endocannabinoids described for the rat studies.

### Toxicology and safety studies with AEF0117 in vitro and in laboratory animals

#### Initial in vitro toxicity screening

A toxicity screen for AEF0117 and other parent compounds was conducted at the initial compound selection stage. Three models were used: (1) neurotoxicity in primary culture of rat cortical neurons; (2) hepatotoxicity and biliary function in primary culture of rat hepatocytes in a sandwich configuration; and (3) genotoxicity measuring histone H2AX phosphorylation (γH2AX) in HeLa cells. AEF0117 was tested up to 100 µM. These studies were performed by the CRO Fluofarma.

#### Neurotoxicity

The cytotoxic effect of AEF0117 in primary culture of rat (embryonic day 19 (E19) embryos) cortical neurons was determined by analyzing the percentage of cytolyzed neurons over time by time-lapse imaging with a fluorescent cytolysis marker. Primary cortical neurons from E19 rat embryos were plated in 96-well plates. After 10 d of culture, neurons were treated with AEF0117 (0 µM, 10 µM, 30 µM and 100 µM) or staurosporine (100 nM, used as a reference compound) and a soluble fluorescent cytolysis marker. N-methyl-2-pyrrolidone (NMP) was used as a solvent with a final concentration of 0.1% in all experimental conditions. After treatment, the cells were followed by time-lapse imaging for 72 h and then permeabilized. This procedure measured cytolysis over time as a percentage of the total number of cells per well.

#### Hepatotoxicity and biliary function

The cytotoxic effect of AEF0117 in primary culture of rat hepatocytes (from 10–12-week-old male Wistar rats) was analyzed by measuring the percentage of cytolyzed hepatocytes over time by time-lapse imaging with a fluorescent cytolysis marker. The number of bile canaliculi after 48 h of treatment was determined using a fluorescent bile salt analog. Primary rat hepatocytes from 10–12-week-old Wistar rats (from Janvier Labs) were isolated using a two-step collagenase perfusion method and plated in 96-well plates. After 24 h in vitro, cells were covered with a layer of Matrigel to perform a sandwich configuration culture. After 24 h, cells were treated with AEF0117 (0, 0.1, 0.3, 1, 3, 10, 30 and 100 µM final concentrations) and a fluorescent cytolysis marker and then monitored by fluorescent and phase-contrast time-lapse imaging for 48 h. Cells were then stained using a fluorescent bile salt analog to measure the bile canaliculi network state and the Bsep pump activity. NMP was used as a solvent with a final concentration of 0.1% in all experimental conditions. Acetaminophen (Sigma-Aldrich, reference A7085) was added as a positive control of hepatotoxicity at 30 mM. Cyclosporin A (Sigma-Aldrich, reference 30024) at 1 µM was added as a positive control of biliary canaliculi loss. Data were acquired with Incucyte Base software (Sartorius).

#### Genotoxicity

These studies were performed in HeLa cells by measuring histone H2AX phosphorylation (γH2AX), which is the cellular response to DNA damage resulting in double-stranded DNA breaks. HeLa cells were seeded in 96-well plates and cultured for 24 h. Cells were then treated with AEF0117 at 0.1, 0.3, 1, 3, 10, 30 and 100 µM final concentrations for 24 h. NMP was used as a solvent with a final concentration of 0.1% for all experimental conditions. Etoposide at 3 µM was added to each plate as a positive control of genotoxic effects. Immunofluorescence was assessed on treated cells using a specific antibody against the phosphorylated histone γH2AX. Nuclei were stained with a fluorescent DNA intercalating agent. The stained cells were imaged and analyzed on a BD Pathway 855 imager (×20 objective, BD Pathway software suite, BD Biosciences).

#### Mutagenic and genotoxic effects (GLP studies)

These studies were performed by the service provider Institut Pasteur de France.

##### Ames’s test

The mutagenic activity of AEF0117 was first assessed in four *Salmonella typhimurium* strains (TA1535, TA1537, TA98 and TA100) and in two *Escherichia coli* strains (WP2 (pKM101) and WP2 uvrA (pKM101)) tested in either the presence or absence of metabolic activation, according to OECD 471 guidelines, in three independent assays. Five nominal doses (8.16, 24.5, 81.67, 245 and 816 µg per plate) of AEF0117 were tested. The dose of 816 µg per plate is approximately a concentration of 84 µM.

##### Chromosome aberration in human lymphocytes

The genotoxic activity of AEF0117 was also assessed by means of the in vitro metaphase analysis test evaluating chromosome aberration in human lymphocytes according to the ICH S2 (R1) guideline and the OECD 473 guideline. Human lymphocytes were taken from young (18–35 years old) healthy non-smoker individuals.

This assay was carried out both with and without metabolic activation using Aroclor 1254-induced S9 from rat livers (5%). In the test performed without metabolic activation, two treatment durations were studied: (1) 4 h + 16 h recovery period (short-term treatment) and (2) 20 h without recovery period (continuous treatment). In the test performed with metabolic activation, the treatment period of 4 h + 16 h recovery period with 5% S9 mix was used. The nominal concentrations of AEF0117 tested ranged between 0.64 µg ml^−1^ and 40.8 µg ml^−1^ after a factor 2 progression.

##### Micronucleus test in rat bone marrow

The potential in vivo genotoxic activity of AEF0117 was tested using the in vivo micronucleus test in rat bone marrow, in compliance with OECD guideline 474 (2016). AEF0117 was administered by the oral route (gavage) once a day for 2 d. Male OFA Sprague Dawley rats received two administrations of AEF0117 (65, 500, 1,000 and 2,000 mg kg^−1^) at 24-h intervals. The dose of 65 mg kg^−1^ was tested because a series of PK studies using doses of AEF0117 up to 1,000 mg kg^−1^ in male rats demonstrated that 65 mg kg^−1^ in corn oil generated the highest plasma exposure. The rats were killed 24 h after the last AEF0117 administration, and the bone marrow was harvested. As a control for the genotoxicity induction, a single i.p. injection 24 h before sampling of the reference substance cyclophosphamide (CPA, Baxter, batch 5K044J in NaCl at 0.9% in distilled water, at a dose of 25 mg kg^−1^ under a volume of 10 ml kg^−1^) was used. In parallel to the main genotoxicity assay, five additional groups of three male rats received one injection of the vehicle or the test item at 65, 500, 1,000 and 2,000 mg kg^−1^, and the plasma concentrations of AEF0117 were evaluated 5 h after administration.

#### GLP safety pharmacology tests

These studies were carried out following the general requirements of GLP, and the study design followed the ICH S7A guideline for Safety Pharmacology. These studies were performed by the CRO European Research Biology Center (ERBC). Data collection and analysis were performed using RS/1 software (release 6.3, Applied Materials).

##### hERG tail currents

The aim of this study was to assess possible effects of AEF0117 on hERG tail current in stably transfected HEK293 cells. The following treatments were tested: Tyrode’s solution; AEF0117 vehicle (0.3% DMSO in Tyrodeʼs solution); AEF0117 at 10.98 × 10^*−*8^ M, 10.98 × 10^*−*7^ M and 10.98 × 10^*−*6^ M. E-4031 was used as positive control and was tested in one separate HEK293 cell to support the validity of the method used. Cells were clamped to −80 mV, depolarized to 0 mV for 5 s allowing activation of hERG current and repolarized to −50 mV for 5 s allowing hERG tail current to deactivate. This experimental procedure was repeated at a frequency of 0.06 Hz. Currents were filtered at 1 kHz and acquired at the frequency of 2 kHz. Amplitude of hERG tail current was measured during the repolarizing pulse from 0 to −50 mV. Cells were perfused with Tyrodeʼs solution, AEF0117 vehicle, and then with AEF0117 solutions for at least 5 min until steady state was reached for each perfusion period. Currents were measured before and after exposure to the test compound. The individual data were collected using pClamp software (release 8.2, AXON Instruments, Molecular Devices).

##### Irwin test

The aim of this study was to assess potential neurobehavioral effects and effects on body temperature of AEF0117 after single oral administration in the rat. The study involved four groups of six male Wistar rats weighing between 154.0 g and 185.9 g. Groups were dosed, respectively, with vehicle (corn oil, 4 ml kg^−1^) or with AEF0117 at 2, 9 or 36 mg kg^−1^ in 4 ml kg^−1^ corn oil. On study day, animals were first scored by the Irwin standardized observation battery, and body temperature was measured. Subsequently, rats were dosed by the oral route with one AEF0117 dose or its vehicle in a volume of 4 ml kg^−1^. Irwin scores and measurement of body temperature were done again at 1, 3, 6, 8 and 24 h after dose.

##### Respiration in unrestrained conscious rats

The aim of this study was to assess effects of a single oral administration of AEF0117 on respiratory parameters (respiratory rate, peak inspiratory and peak expiratory flows, inspiration and expiration times, airway resistance index, tidal volume and minute volume) measured by whole-body plethysmography in conscious rats. The study involved four groups of six male Wistar rats weighing between 283.2 g and 346.3 g (8–11 weeks old). Groups were dosed, respectively, with vehicle (corn oil, 4 ml kg^−1^) or with AEF0117 (2, 9 or 36 mg kg^−1^ in 4 ml kg^−1^ corn oil). Animals had access only to water the day before the study. On the study day, animals were placed in the plethysmograph, and measurements were started immediately. The whole-body plethysmography method measures variations in air flow due to thoracic cage movements during respiration and enables the measurement of respiratory parameters in the conscious freely moving animal. At least 15 min after the start of measurements, animals were administered AEF0117 or its vehicle by the oral route. Respiration was recorded for 6 h after dosing using the ART computerized acquisition system, release 4.33 (Data Sciences International). Respiratory parameters were determined from analysis of respiratory cycles.

##### Blood pressure, heart rate, ECG and body temperature in conscious dogs

The aim of this study was to evaluate possible effects of AEF0117 on blood pressure, heart rate, body temperature and ECG after a single oral administration to four male Beagle dogs weighing between 13.3 kg and 15.0 kg (17–40 months old). The dogs had telemetry transmitters for arterial blood pressure, body temperature and ECG measurements. The study was conducted in two parts. In part I, each animal received vehicle (corn oil 4 ml kg^−1^) or AEF0117 (2, 9 or 36 mg kg^−1^ in 4 ml kg^−1^ corn oil) by the oral route according to an ascending-dose design with a washout period of 1 week between doses. Telemetry measurements of arterial blood pressure, heart rate, body temperature and ECG (epicardial lead II) started at least 2 h before each dosing and continued for at least 24 h after dosing and was performed using the ART computerized acquisition system, release 4.33 (Data Sciences International). In part II, animals again received AEF0117 at either 9 mg kg^−1^ or 36 mg kg^−1^ (*n* = 2 per dose level) by the oral route for blood sampling and observation.

#### Ninety-one-day repeated oral toxicity study in rats and dogs

In addition to the GLP 91-d repeated oral toxicity study, described in this article, the oral toxicology of AEF0117 was studied in non-GLP (maximal tolerated dose and 14-d administration) and in a 28-d repeated administration (2, 9 and 36 mg d^−1^) GLP study in rats (Sprague Dawley SPF, 7 weeks old) and dogs (Beagle, 7–8 months old). In all studies, AEF0117 appeared to be well tolerated with no major toxic effects. Studies were conducted by the CRO ERBC.

The 91-d GLP study included additional animals in the control and highest dose groups (drug withdrawal groups) to study the reversibility, persistence or delayed occurrence of toxic effects for 28 d after treatment. Satellite groups of animals dosed with the vehicle or the test item at each dose level were included to assess TK parameters. Any toxicity seen during repeated oral administration of AEF0117 was evaluated in male and female rats in accordance with general recommendations found in OECD guideline 407, adopted on 16 October 2008, and the EMEA Note for Toxicokinetics: A Guidance for Assessing Systemic Exposure in Toxicology Studies (CPMP/ICH/384/95; ICH S3A), adopted in June 1995.

**Design of the 91-d oral toxicity study in the rat**

**Table:** 

Group	Main groups, number of animals and sex	Satellite groups, number of animals and sex	Drug withdrawal groups^a^, number of animals and sex	Treatment vehicle or test item (mg kg^−1^ d^−1^)
1	10 M, 10 F	3 M, 3 F	10 M, 10 F	Vehicle
2	10 M, 10 F	6 M, 6 F	–	2
3	10 M, 10 F	6 M, 6 F	–	20
4	10 M, 10 F	6 M, 6 F	10 M, 10 F	65

^a^ Four-week treatment-free recovery period. AEF0117 or its vehicle was administered once a day between 8:00 and 12:00 at each chosen dose level by the oral route for 91 consecutive days in a volume of 4 ml kg^−1^ body weight. F, females; M, males.

**Design of the 91-d oral toxicity study in the dog**

**Table:** 

Group	Number of animals and sex	Drug withdrawal groups^a^, number of animals and sex	Treatment vehicle or test item (mg kg^−1^ d^−1^)
1	3 M, 3 F	2 M, 2 F	Vehicle
2	3 M, 3 F	–	2
3	3 M, 3 F	–	20
4	3 M, 3 F	2 M, 2 F	65

^a^ Four-week treatment-free recovery period. F, females; M, males.

In both species, AEF0117 was tested at three doses (2, 20 and 65 mg kg^−1^ administered in corn oil at 4 ml kg^−1^). The 65 mg kg^−1^ dose was chosen as the highest dose because it is the oral dose producing the highest possible exposure to AEF0117 in rats as shown in preliminary PK studies. The 65 mg kg^−1^ dose of AEF0117 should provide an adequate safety margin because it is 13,000 times higher than the most frequently observed ID_50_ (that is, 0.005 mg kg^−1^) for inhibiting THC effects in mice, rats and non-human primates.

In both species, morbidity and mortality checks were done twice daily. General observations were done before the first dose and then daily. A full clinical examination was performed weekly. Functional and neurobehavioral tests (including temperature measurement) were performed before the first dosing and then monthly, on the last week of the treatment period and at the end of the drug withdrawal period. All clinical observations were performed at approximately 3 h after dose. Body weight was recorded before dose and then weekly. Food consumption was measured weekly. Ophthalmological examination was performed before the first dosing, during the last week of treatment and then at the end of the recovery period.

In rats, blood samples for hematology and clinical chemistry parameters and urine were collected at the end of the first month of treatment (day 29), at the end of the treatment period (week 14) and then at the end of recovery period (week 18).

In dogs, blood samples for hematology parameters were collected before the start of treatment (pre-dose) and then monthly up to the end of the treatment period and at the end of the drug withdrawal period (week 18). Blood samples for clinical chemistry analysis and urine were collected before the start of treatment (pre-dose), after the first month of treatment (week 4 or 5), at end of the treatment period (week 13 or 14) and at the end of the drug withdrawal period (week 18 or 19). Urine was collected before the start of treatment (pre-dose), at the end of the treatment period (week 13) and at the end of the drug withdrawal period (week 18). ECG and systolic blood pressure (SBP) and respiratory measurements were done before the start of treatment, at the end of the treatment period and at the end of the drug withdrawal period.

In both species, all animals from the main groups were sacrificed on week 14 (day 92), and all animals from the drug withdrawal groups were sacrificed on week 18 (day 122). Selected organs were weighed, fixed and preserved at necropsy and examined histopathologically. Epididymis was sampled for sperm analysis and testicular staging.

#### Effects of a repeated treatment with AEF0117 and rimonabant on food intake and body weight in mice

These experiments evaluated the effect of repeated treatment with AEF0117 and the CB_1_ orthosteric antagonist rimonabant on food intake and body weight in a diet-induced obese (DIO) mouse model. Body weight and food intake were studied because they are reduced by repeated treatment with rimonabant both in mice^31^ and in humans^13^. DIO mice were used because the effects of CB_1_ antagonists are of greater amplitude in obese mice than in lean mice. Male C57BL/6J mice (8 weeks old) were fed ad libitum with a high-fat diet (HFD) for 8 weeks before the start of the pharmacological treatments. During pharmacological treatments, the HFD was maintained, and food intake and body weight were measured daily. The food consumed was calculated by subtracting the food left in the hoppers from the initial pre-weighed amount. In the first experiment (*n* = 7–10 per group), the effect of AEF0117 (0, 0.005, 0.015 and 0.05 mg kg^−1^, in 2 ml kg^−1^ corn oil) for 4 weeks was analyzed. In the second experiment (*n* = 8 per group), the effects of AEF0117 (0.5 mg kg^−1^ and 15 mg kg^−1^ in 5 ml kg^−1^ corn oil) were compared to those of rimonabant (10 mg kg^−1^ in 5 ml kg^−1^ corn oil) over 2 weeks of treatment. AEF0117 and rimonabant were administered by oral gavage once a day 2 h before the start of the dark phase of the light/dark cycle.

#### Effects of AEF0117 on precipitated withdrawal in mice

These experiments aimed to evaluate the ability of AEF0117 and rimonabant to precipitate withdrawal in male mice (CD-1 Swiss, 8–9 weeks old) chronically treated with THC 20 mg kg^−1^ twice a day i.p. for 5 d. The effects of rimonabant (10 mg kg^−1^, i.p.) and of AEF0117 (0.15 mg kg^−1^, orally) were studied in independent experiments in CD-1 Swiss mice. From day 1 to days 4–5, mice were injected i.p. with vehicle or THC (20 mg kg^−1^) twice per day. On the last day of treatment, mice in the THC group received rimonabant or AEF0117. All other animals received the respective vehicle. Recordings were analyzed for 45 min immediately (rimonabant) or 3 h (AEF0117) after administration. The dose and schedule of rimonabant administration chosen was shown to precipitate THC withdrawal in mice^14^. For the measure of precipitated withdrawal, mice were placed in a novel home cage, and a video camera was positioned in front of each cage to record behavior. Scoring was performed for 1 min every 5-min period. Two withdrawal signs were analyzed—paw tremors and head shaking—because they are the most common signs of THC withdrawal observed in mice^14^.

#### Effects of a repeated administration of AEF0117 on anxiety-related and depression-related behaviors in mice

These experiments aimed to evaluate whether repeated treatment with AEF0117 or rimonabant increased anxiety-related and depression-related behaviors in male mice (C57BL/6J). Anxiety-related and depression-related behaviors have been shown to increase after repeated administration of CB_1_ orthosteric antagonists in rodents^32,33^ and in humans^13^. Anxiety-like behaviors were studied in the elevated plus maze (EPM), a rodent model used to evaluate the putative anxiogenic or anxiolytic effects of pharmacological compounds. Depression-related behaviors were studied using the sucrose preference test as a model of anhedonia, one of the cardinal symptoms of depression.

The EPM apparatus comprises four elevated arms arranged in a cross-like shape, with the two opposite arms enclosed by walls and the other two arms open. For all experiments, mice were placed in the center of the EPM and were free to explore the maze for 5 min after receiving the treatment. The time spent and the number of entries into the open and closed arms were measured by an automatic video tracking system (EthoVision XT version 12, Noldus Information Technology). A decrease in the percentage of visits and/or the time spent in open arms is considered an index of increased anxiety.

The sucrose preference test was done in the home cage. Two identical bottles, one containing water and the other containing a 2% sucrose solution, were placed in the hopper of each cage. The mice had unlimited access to water and sucrose solutions during the active phase—the dark phase of the light/dark cycle that started at 20:00. The volume of water and of sucrose solution consumed was measured over two 1.3-h intervals, the first between 19:00 and 20:30 and the second between 20:30 and 22:00. At each timepoint, the bottles were weighed, and the intake volume was calculated by subtracting the initial bottle weight from the final bottle weight.

Male C57BL/6J mice (*n* = 6–8 per group) received either one daily administration of AEF0117 (0.05, 5 or 15 mg kg^−1^, orally), rimonabant (10 mg kg^−1^, i.p.) or the respective vehicle for 28 d. The EPM assessment was done on day 26, and the sucrose preference test was done on day 28. All the behavioral procedures started 3 h after AEF0117 or its vehicle and 30 min after rimonabant or its vehicle.

#### Effects of AEF0117 on glucocorticoid secretion in mice

These experiments aimed to evaluate in mice the effects of AEF0117 on plasma concentrations of corticosterone, the main glucocorticoid produced by the adrenal gland in rodents, corresponding to cortisol in humans. The effects of AEF0117 on corticosterone levels were studied because the orthosteric CB_1_ antagonist rimonabant increases plasma corticosterone concentrations^34^.

The effects of AEF0117 (0.3 mg kg^−1^ and 10 mg kg^−1^, orally) or vehicle on plasma corticosterone levels were studied in male and female CD-1 Swiss mice (9–10 weeks old). Blood sampling was performed 2, 5, 8 and 24 h after dosing (*n* = 3 per sex per dose per sampling time). For blood sampling, mice were anaesthetized under isoflurane, and blood was collected by cardiac puncture. Blood was centrifuged, and plasma was taken and frozen at −80 °C until quantifications of corticosterone by GC–MS/MS.

### Efficacy studies with AEF0117 in laboratory animals

#### Effects of AEF0117 on i.v. self-administration of the CB_1_ agonist WIN55,212-2 in mice

The effect of AEF0117 on i.v. self-administration of WIN55,212-2 was measured in male CD-1 Swiss mice. Before the start of the self-administration sessions, mice were implanted under anesthesia with catheters into the right jugular vein. The self-administration experiments were conducted 3 d after surgery in mouse operant chambers equipped with one ‘active’ hole and one ‘inactive’ hole. When the mouse inserted its nose (nose poke) in the active hole, it received an i.v. infusion of WIN55,212-2 (12.5 µg kg^−1^); nose poking in the inactive hole had no scheduled consequences. Mice were trained under a fixed ratio 1 (FR1) schedule of reinforcement.

Two-hour daily self-administration sessions were conducted 6 d per week for 18 d. Mice received corn oil vehicle (2 ml kg^–1^) orally on days 9 and 10 to be habituated to the oral gavage procedure. On day 11, mice were randomized into two groups (*n* = 13 per group)—one received AEF0117; the other received corn oil vehicle 3 h before the start of the self-administration session for eight consecutive days. AEF0117 was administered at 5 µg kg^–1^ for the first 4 d and at 15 µg kg^–1^ for the remaining 5 d.

#### Effects of AEF0117 on i.v. self-administration of THC and reinstatement of THC-seeking in non-human primates

These experiments aimed to evaluate the effect of AEF0117 on the reinforcing effects of THC in non-human primates (squirrel monkeys, *Saimiri sciureus*). Two experimental models were used: (1) THC i.v. self-administration and (2) THC-induced reinstatement of THC seeking. These two approaches were used to model the maintenance of daily cannabis use and cannabis seeking after a period of abstinence. For all the experiments, AEF0117 was administered orally in a grape in a volume of 0.1 ml of corn oil 4 h before testing.

For the self-administration experiments, four male squirrel monkeys (800–1,100 g; the estimated age was 17 years for three monkeys and 14 years for the fourth one) were used because this species reliably self-administers i.v. THC. Monkeys were trained to lever press for an i.v. injection of THC (4 µg kg^–1^ per injection) under a 10-response fixed ratio schedule of drug injection (FR10; each 10th response on the lever produced an injection of THC, followed by a 60-s timeout). Number of lever presses and number of injections per session were recorded. The effects of AEF0117 (0, 1.5, 5, 15 and 50 µg kg^–1^) were tested for 3 d each in ascending order, with a minimum 6-d washout period between the different doses and demonstration of a stable baseline for three consecutive days.

For the THC-induced reinstatement of THC seeking, the monkeys underwent daily extinction sessions during which lever presses led to vehicle infusions and the visual cues previously paired with THC infusions but not THC. After at least two extinction sessions, when responding had reached a low level, the effect of pre-treatment with AEF0117 (1.5, 5 and 15 µg kg^–1^) or vehicle on THC-induced (40 µg kg^–1^, i.v.) reinstatement of THC seeking was determined. THC injections were given immediately before the start of the test sessions. During testing, lever presses (FR10) continued to produce only vehicle injections and the THC-paired cues. The effect of 15 µg kg^–1^ of AEF0117 on vehicle priming was also tested to determine whether AEF0117 alone would affect responding after extinction. Recommendations from the Guide for the Care and Use of Laboratory Animals (8th edition) and guidelines of the Institutional Animal Care and Use Committee of the Intramural Research Program, NIDA, NIH, DHHS, were followed. Animals were maintained in facilities fully accredited by the AAALAC. Operation of the experimental chambers and data collection were controlled by IBM computers using the MED Associates MED-PC software package.

#### Effects of AEF0117 on THC’s effects on food intake

Cannabis use can increase food intake and preference for palatable food^35^. The effects of THC on food intake were studied using the fasting–refeeding model^36^ in male CB_1_-flox (CB_1_^f/f^) mice (8 weeks old). CB_1_^f/f^ mice were backcrossed into the C57BL/6N for at least 7–8 generations and carry a floxed *Cnr1* gene, which codes for the CB_1_. These mice were bred in-house (Neurocentre Magendie), have a wild-type expression of CB_1_ receptors and were used to avoid stress effects on food intake caused by transportation from commercial vendors. The effect of AEF0117, administered orally at three doses (5, 15 and 50 µg kg^–1^) plus vehicle, in combination with THC (1 mg kg^–1^, i.p.) or its vehicle, on food intake was assessed in 24-h food-deprived mice that were re-fed 30 min after THC, and food intake was measured for 1 h afterward. Food intake and body weight were measured daily before the dark phase in animals housed in their home cage. Spillage of food was checked daily. The food consumed was calculated by subtracting the food left in the hoppers from the initial pre-weighted amount. Independent groups of animals (at least *n* = 8 per group) were used for each treatment condition.

#### Effects of AEF0117 on THC-induced increase in psychomotor stimulation

This behavior was studied because it is considered as a model of psychotic-like symptoms that can be observed after cannabis use^18^. Locomotor activity in an open field with a square-patterned floor was measured for 5 min in C57BL/6N male mice 45 min after THC or vehicle administration (i.p.) by counting the number of squares crossed. The effect of AEF0117 at six doses (0, 0.15, 0.5, 1.5, 15 and 150 µg kg^–1^) was tested in combination with THC (0.3 mg kg^–1^, i.p.) and compared to vehicle. Independent groups of animals (at least *n* = 10 per group) were used for each experimental condition.

#### Effects of AEF0117 on THC-induced impairment of PPI

The PPI test was chosen because it is a model of impaired sensory motor gating observed in psychosis and after THC administration^37^. The effect of AEF0117 at six doses (0, 0.5, 1.5, 15, 30 and 50 µg kg^–1^) was tested on the impairment in PPI induced by THC (10 mg kg^–1^, i.p.) and compared to vehicle in C57BL/6N male mice (9 weeks old). PPI was measured using automated PPI cages and recording the animal’s startle reactions (SR-LAB Startle Response System software, San Diego Instruments). Each mouse (at least *n* = 8 per group) was placed in the PPI cage for 45 min, 60 min after THC administration. The test included different types of trials consisting of background noise, a startle stimulus (S; 120 dB) alone, one of the pre-pulse (82 dB) stimuli alone or a combination of each pre-pulse stimulus (PPI), followed by the startle stimulus (PPI-S). The startle response after the pulse presentation was recorded, and an index of PPI was calculated (% PPI = 100 × (S − PPI-S)/S).

#### Effects of AEF0117 on THC’s memory effects

In mice, long-term memory can be evaluated using the object recognition test in which memory of a specific object is evaluated 24 h later. CD-1 Swiss male mice (9 weeks old) received an acute oral administration of AEF0117 (5 µg kg^–1^) or corn oil (5 ml kg^–1^) vehicle, followed 3 h later by an i.p. injection of THC (6 mg kg^–1^; 10 ml kg^–1^). Ten minutes before THC injection, mice were allowed to explore two identical objects in an ‘L’-shaped maze. The day after, one object was replaced by a novel one. According to the spontaneous novelty preference, mice investigate novel objects for a longer period than familiar objects. The comparison of the time spent exploring the familiar and novel objects is used as an index of discrimination between familiarity and novelty. Therefore, this parameter is used to evaluate object recognition performances and, consequently, long-term memory.

#### Effects of AEF0117 on THC’s effects on social interaction

Social interaction was studied because social withdrawal, defined as the indifference or lack of desire to have social interaction, is observed in psychosis^38^. Social interaction can be evaluated in mice by measuring their spontaneous preference for an encounter with a congener as compared to a non-social encounter. In this paradigm, the acute administration of THC (3 mg kg^–1^) reduces social preference^18^, providing a model of the social withdrawal endophenotype in psychosis.

Mice (8–10-week-old adult male C57BL/6N) were tested in an open field (35 × 35 cm) with two plastic containers (plastic cylinders of 8-cm diameter with holes for odor interaction) placed at two opposite corners, one of them hosting a mouse, the other remaining empty. Each corner was designated as a ‘social’ and ‘non-social’ zone as an 8-cm area surrounding the containers. For each experimental group, the position of the container with the mouse was counterbalanced. The experimental mouse was placed in the center of the open field to explore for 5 min, filmed by a camera. The time spent in both zones was counted (the animal was in a zone when all of its four paws were inside the drawn lines). Social index was calculated as follows: social index = time spent in the ‘social zone’ / total time spent in both zones. Mice were administered THC (3 mg kg^–1^, i.p.) or its vehicle 2 h before entering the open field. AEF0117 (5, 15 or 50 µg kg^–1^) or its vehicle was administered orally 3 h before THC or its vehicle.

#### Effects of AEF0117 on THC’s effects on reality testing

Alterations in the mental representation of stimuli leading to mismatches between perception and reality are key features of positive psychotic symptoms^18^. In rodents, the ‘reality testing’ task assesses the potential mismatch between internal representation of a stimulus (odor or taste) and the reality they predict.

This test is based on the conditioned aversion paradigm. Two stimuli of equal valence, typically a taste and an odor, are first presented simultaneously in a repeated manner (six times). One of the stimuli, for instance the odor, is then associated with a noxious event (that is, lithium chloride (LiCl)-induced gastric malaise). After conditioning, mice show a specific aversion for the stimulus paired with the noxious event (that is, the odor) but not for the neutral stimulus (that is, the taste), although the odor and the taste were previously presented together. These responses suggest that mice built specific representations of each of the stimuli. However, psychotogenic drugs, including MK-801, amphetamine and THC, lead to aversion for both stimuli, including the one that was not conditioned with the noxious event (mediated aversion). This effect suggests that psychotogenic drugs induce an inaccurate representation of the ‘neutral’ stimulus. These alterations are reversed by the atypical anti-psychotic risperidone. Therefore, impairment of ‘reality testing’ by THC as well as other psychotogenic drugs in mice shows both face and predictive validity for the investigation of positive psychotic symptoms.

Reality testing consists of four phases with different pairings (a pairing refers to the association of two stimuli at a time): habituation (3 d), pre-conditioning (six pairings of odor and taste, 12 d), conditioning (that is, three pairings of odor and injection of an agent inducing malaise, LiCl, 6 d), recovery (1 d with water) and finally the tests (mediated aversion and direct aversion tests).

Mice (8–10-week-old adult male C57BL/6N) were water deprived for 24 h before starting habituation that consisted of 1-h access to water per day for 3 d to get animals used to receiving liquid every day for 1 h, to reach a consistent consumption over the protocol. This was followed by the pre-conditioning phase in which the mice were given 1-h access per day to a mixed solution (O1T1) with one odor (either almond or banana, O1) and one taste (either maltodextrine or sucrose, T1) in water. On day 2, mice received 1-h access to the solution with the odor and taste not given the previous day (O2T2). After six pairings of O1T1 and O2T2 (12 d), the conditioning phase was started.

At the first day of conditioning, mice were given 1-h access to odorized water (O1), directly followed by i.p. injection of saline. The next day, mice were given access to the second odorized water (O2), directly followed by an i.p. injection of LiCl (0.3 M) at a volume of 10 ml kg^–1^. After three pairings of O1/saline and O2/LiCl (6 d), mice were given a recovery day with 1-h access to water.

The next day, mediated aversion was assessed by performing a two-choice test with two bottles of water containing one of the two tastes: the taste paired with the odor associated with LiCl injection (T2), called C+, or the taste paired with the other odor that was associated with the saline injection (T1), called C−. In this test, the appearance of mediated aversion is signaled by a decrease of the consumption of water containing the taste paired with the odor that had been previously paired with LiCl (T2, C+) as compared to the water containing the other taste that was associated with the odor that was never paired with LiCl (T1, C−). The tests results were expressed by the aversion index as follows:

$$\begin{array}{l}Aversion\,index = (Consumption\,of\,{\rm{C}}{\hbox{-}}-Consumption\,of\,{\rm{C}}+)\\ \qquad \qquad \qquad \qquad /Total\,consumption\end{array}$$

Mice (*n* = 10–20 per condition) were administered THC (1 mg kg^–1^, i.p.) or its vehicle 2 h before the two-choice test assessing mediated aversion. AEF0117 (15 µg kg^–1^ or 50 µg kg^–1^) or its vehicle was administered orally 3 h before THC or its vehicle.

#### Effects of AEF0117 on THC-induced catalepsy

Catalepsy was studied because it can be considered a model of catatonia, which is one of the negative symptoms of psychosis and has been observed after the use of synthetic cannabinoids^19^.

The cataleptic effects of AEF0117 (0, 1.5, 5, 15 or 50 mg kg^–1^) in 5 ml kg^–1^ corn oil was studied after THC (10 mg kg^–1^) in C57BL/6J male mice. THC was injected 3 h after AEF0117 administration. Measures began 30 min after THC injection using the catalepsy bar test. Forepaws were placed on a bar fixed horizontally at 3.5 cm from the bench surface. The latency to move from the bar was recorded with a cutoff time fixed at 420 s (7 min). Each mouse completed up to four consecutive trials. The maximum latency shown in one trial was selected as the measure of catalepsy.

#### Effects of AEF0117 on THC-induced DA release in the Nac

These experiments evaluated the effects of AEF0117 on THC-induced increases in DA release in the Nac of freely moving rats, as measured by microdialysis. DA release in the Nac was studied because it is considered a primary mechanism by which most drugs produce reinforcing effects, including THC^20,21^.

The effect of AEF0117 at three doses (5, 15 or 50 µg kg^–1^, orally) or vehicle was tested after THC (1 mg kg^–1^, i.p.) administration to male Sprague Dawley rats. THC was solubilized in 0.9% NaCl containing ethanol (2%) and Tween 80 (2%) that was also used as control vehicle and administered i.p. in a volume of 1 ml kg^–1^.

Rats (*n* = 5–7 per group, 350–380 g) were implanted under anesthesia with a guide cannula just above the shell subregions of the right Nac. On the day of the experiment (5–7 d after surgery), freely moving rats received AEF0117 or vehicle, and the microdialysis probe was implanted into the guide cannula that was then perfused with artificial cerebrospinal fluid. Dialysates were collected every 15 min. Then, 180 min after the beginning of the perfusion, all animals received an injection of THC, after which DA outflow was measured for 120 min. The concentrations of DA in dialysate samples were analyzed by reverse-phase high-performance liquid chromatography (HPLC) coupled with electrochemical detection, as described previously^39^. Data were acquired using Azur (Datalys). DA content in each sample was expressed as the percentage of the average baseline level calculated from the three fractions preceding THC administration. The AUC was calculated for each group from sampling time 0–60 min after THC injection.

All experiments in rodents were conducted in strict compliance with European Union recommendations (2010/63/EU) and with the guidelines of the European Communities Directive 86/609/EEC regulating animal research. All procedures were reviewed and approved by the local ethics committee of the University of Bordeaux and the local ethics committee of Pompeu Fabra University (CEEA-PRBB) and were approved by the French Ministry of Agriculture and Fisheries. Maximal efforts were made to reduce the suffering and the number of animals used.

### Data collection and analysis

Statistical analyses were carried out using GraphPad Prism, Statistica (Statsoft) or SigmaPlot (Systat Software) software. The effect of treatment (AEF0117 and vehicle) was determined using one-way ANOVA with AEF0117 dose as the between-subject factor, two-way repeated-measures ANOVA with AEF0117 dose as the between-subject factor and time (or session) as the within-subject factor. When appropriate, significant main effects were analyzed by subsequent multiple paired comparisons or multiple comparisons versus the control group using the appropriate post hoc test (Dunnett, Tukey or Sidak test). Different but similar post hoc tests were used because the experiments were performed by independent research groups that used similar but not identical statistical procedures. The one-tailed Student *t*-test was only used to analyze the effect of AEF0117 on reality testing and the effect of rimonabant on sucrose intake and behavior in the EPM. For non-parametric analysis, a Kruskal–Wallis ANOVA was used, followed, when appropriate, by the Mann–Whitney test for pairwise comparisons. Unless otherwise stated, all the performed statistical tests were two-sided. The ID_50_ or IC_50_ was estimated by nonlinear regression using the ‘log(inhibitor) versus response’ equation model with GraphPad Prism software. All results are expressed as mean ± s.e.m. The significance threshold was set at *P* < 0.05.

### Formulation of AEF0117 used in the clinical studies

AEF0117 was administered in soft oval gelatin capsules containing a solution of AEF0117 in a pharmaceutical-grade corn oil produced by the CDMO Catalent using GMP conditions. Matched placebo control was an identical soft gel capsule containing the corn oil. All excipients used were compliant with current European Pharmacopoeia (Ph. Eur.) monographs and United States Pharmacopoeia-National Formulary (USP-NF) monographs.

### Clinical studies in healthy volunteers: single and multiple ascending doses

The single-dose (SAD; NCT03325595) and multiple-dose escalation (MAD; NCT03443895) phase 1 studies were the first-in-human trials with the NME AEF0117. The protocols were prospectively approved under the FDA’s IND 126501 and by the IntegReview institutional review board (IRB00001035) and the New York State Psychiatric Institute (NYSPI) institutional review board (IRB00000488). Written informed consent was obtained before performing any study-related procedures, and the trials were conducted in accordance with GCP.

#### Study procedures and conduct

The SAD and MAD phase 1 studies investigated AEF0117 safety and PK in healthy adult volunteers and were conducted at the Biotrial clinical facility. Biotrial also provided clinical support, including protocol development, study monitoring, data management, statistical analyses and clinical study report preparation.

Biotrial Bioanalytical Services, a subsidiary of Biotrial, carried out the analysis of AEF0117 in plasma and the PK analysis.

Clincase version 2.6 (Quadratek Data Solutions) was used for data management and SAS version 9.3 or 9.4 (SAS Institute) for the statistical analysis. Statistical analyses were performed following a SAP signed and filed before the database lock.

#### Study design

The SAD and MAD studies were two phase 1, single-center, double-blind, randomized, placebo-controlled, single-period, single-dose (SAD) and multiple-dose (MAD) escalation trials with AEF0117 in healthy male and female volunteers (18–55 years of age, inclusive). Four single oral dose levels (0.2, 0.6, 2 and 6 mg) were given in the morning on day 1 in the SAD study, and three oral doses (0.6, 2 and 6 mg) were given in the morning of day 1 to day 7 in the MAD study, to independent dose cohorts. The pharmacist and his/her attendant were the only personnel to have access to the randomization list, to prepare the drug for administration, which was packaged in a double-blind manner. Each dose cohort comprised eight volunteers (six (5/1) active and two (1/1) placebo per dose level).

In the SAD study, in each dosing cohort, two sentinel participants (one randomized to AEF0117 and one to placebo) were dosed and observed for at least 24 h before initiating dosing in the remaining six participants. Subsequent participants within a cohort (five randomized to AEF0117 and one to placebo) were dosed with an interval of 5–10 min, following a review by Michael Dobrow (Biotrial) of the available safety data and based on clinical judgment to continue dosing the cohort.

The 2-mg dose was repeated (cohorts 3 and 4) as requested by the FDA because of the asymptomatic increase in creatine phosphokinase (CPK) observed in one participant. In total, 177 male and female participants were screened, and 40 were randomized in a total of five cohorts of eight volunteers. Due to a requirement of the FDA that all laboratory values be within normal limits for the volunteers in the initial two cohorts (0.2 mg and 0.6 mg), many people had to be screened, as this condition is rarely seen (<10% of screened healthy volunteers). During the rest of the study, only volunteers with pathological values were excluded.

In the MAD study, each randomized participant received a single oral dose per day on days 1 through 7. In total, 66 male and female participants were screened, and 24 were randomized for a total of three cohorts of eight volunteers. In the AEF0117 2-mg cohort, some participants (five active treatment and one placebo) received the daily dose on day 1 and days 3–8, as bad weather conditions prevented dosing on day 2 (staff not available). Thus, all day 2 treatment administrations and assessments were canceled for this cohort. As an approximation, plasma PK concentrations and parameters were presented for all participants as if occurring on day 7.

The planned dose-escalation schema for the SAD study was based on preclinical pharmacodynamics (PD), safety and toxicology data translated to humans using body surface ratios to ensure a large safety margin. For the MAD study, safety and PK data from the SAD study were used to determine doses. In both studies, all safety data for a given cohort/dose level were evaluated by a Data Safety Monitoring Board that was guided by predefined stopping criteria. The stopping criteria were based on the seriousness and severity combined with frequency of AEs that would lead to either not proceeding to the next dose level or stopping the trial. Dose escalation was also to be halted if the peak plasma levels exceeded that corresponding to the NOAEL in the most sensitive animal species (that is, the rat) in preclinical toxicology studies. In addition, the maximum AEF0117 dose administered was selected so that the plasma exposures for AEF0117 in humans would not exceed the mean AUC of 41,611 ng × h ml^−1^ and/or mean C_max_ of 4,361 ng ml^−1^, corresponding to the highest dose tested in animals without toxicity at the time of the study.

Administration of AEF0117 to each dose cohort could not occur before participants in the previous dose cohort had been treated and data—that is, safety results and PK from those participants—had been reviewed by the Data Safety Monitoring Board.

Participants were admitted to the research clinic at midday before dosing (day −1). In the SAD study, they remained in-house until day 8 for PK and PD samples and safety assessments. In the MAD study, participants remained in-house until day 14 and returned to the research facility on an outpatient basis to have PK and safety assessments at 216 h and 264 h (day 16 and day 18) after their last dose (day 7). Safety monitoring (physical examinations, vital sign measurements, 12-lead ECGs, clinical safety laboratory tests and AE monitoring) were performed throughout the studies. Psychometrics tests (Bond & Lader VAS, Addiction Research Center Inventory (49-item short form) (ARCI-49) and Profile of Mood States 65 self-report items (POMS-65)) and the Columbia-Suicide Severity Rating Scale (C-SSRS) test were performed at baseline, before dose and at 4 h and 24 h after dose on day 1 and day 7 (MAD study, only) and C-SSRS only at 24 h.

Participants had a final follow-up safety evaluation on day 8 in the SAD study and on day 18 in the MAD study. All participants who had AEs, whether considered associated with the use of the investigational product or not, were monitored for as long as needed to determine resolution.

#### PK analysis

##### SAD study

For each participant, blood samples were collected for analysis of AEF0117 plasma concentration before dose and at the following timepoints: day 1 at 0.5, 1, 1.5, 2, 3, 4, 6, 8 and 12 h after dose and on the morning of day 2, day 3, day 4, day 5, day 4 and day 7 (that is, 24, 48, 72, 96, 120 and 144 h from dosing, respectively).

##### MAD study

Samples were collected at the same timepoints on day 1 and day 7: before dose and 0.5, 1, 1.5, 2, 3, 4, 6, 8 and 12 h after dose. Additional samples were collected on day 2 to day 6 before dose (that is, 24 h from the previous dose) and on days 8, 14 16 and 18 (that is, 24, 48, 72, 96, 120, 144, 168, 216 and 264 h after the last dose at day 7).

For both SAD and MAD studies, the PK parameters were calculated using standard non-compartmental methods for those with sufficient plasma concentration data. Plasma concentrations and PK parameters of AEF0117, including descriptive statistics, were performed separately for each treatment group.

#### Steroids and endocannabinoids analysis

For both the SAD and MAD studies, serial blood sample collections for steroid and endocannabinoid levels were done at day 1 before dose and 0.5, 1, 2, 4, 8, 12 and 24 h after dose. The data from the two studies were combined for analysis. The number of volunteers per group was: placebo (*n* = 16); 0.2 mg (*n* = 6); 0.6 mg (*n* = 12); 2 mg (*n* = 18); and 6 mg (*n* = 12). ALLO, DHEA, PREG, TESTO, CORT, estradiol and PROG, as well as AEA and 2-AG, were assayed. Data were analyzed using a repeated-measures ANOVA with treatment as between-group factor and time after dosing as within-group factor. Data are presented as mean ± s.e.m. of plasma concentrations.

### Clinical study with AEF0117 in research volunteers with CUD (phase 2a)

The protocol for this phase 2a study (NCT03717272) was approved by the FDA (IND 126501) and by the NYSPI institutional review board (IRB00000488). Written informed consent was obtained before performing any study-related procedures, and the study was conducted according to GCP principles. The principal aim of the study was to investigate the effects of AEF0117 on the subjective effects of cannabis related to abuse liability (primary outcome) and on cannabis self-administration (key secondary outcome) in research volunteers with CUD.

#### Data collection

The study was conducted at the Cannabis Research Laboratory (NYSPI, Columbia University Irving Medical Center) and supervised by the CRO ClinSmart, which provided data management, eCRF preparation, data monitoring and safety management. The CRO BioClever contributed to the SAP and did the statistical analysis and medical writing. Biotrial Bioanalytical Services carried out the analysis of AEF0117 in plasma and the PK analysis. Data acquisition for the cognitive tests was performed using Inquisit (Millisecond Software) version 5.0.13. For data management, Panther-EDC version 3.70.3 (EDETEK) was used. Statistical analyses were performed using SAS version 9.3 or 9.4 following a SAP that was signed and filed before the database lock. Both male and female participants were included in the study, but sex was not considered in the data analysis because of the low number of females typically included in initial clinical trials with an NME. In addition, the results are expected to apply equally to both males and females.

#### Study population

Adult male and non-pregnant female individuals 21–60 years of age with CUD who were otherwise healthy were eligible for the study. Twenty-nine volunteers were enrolled in the study (see Table 1 for baseline demographic data and clinical characteristics) and randomized to two treatment groups (block size = 4; allocation ratio = 1:1).

#### Study design

This double-blind, placebo-controlled, randomized, dose-ranging, crossover, single-site study included two independent cohorts that received either a low (0.06 mg every day) or a high (1 mg every day) oral dose of AEF0117 in ascending order. Data from the first dose level were reviewed by the Data Safety Monitoring Board before ascending to the next dose level.

Even though 3–4 doses were initially planned to be tested in four cohorts according to the dose-escalation plan, this study was terminated after the second dose cohort. The reasons for early termination were:

- The results of this study showed that the crossover design used was hampered by a significant influence of dosing sequence on the treatment effect, likely because of a carryover effect that maintained the effects of AEF0117 even after ≥14 d of washout.
- The study, having provided clear evidence that AEF0117 inhibited behaviors related to CUD, had fulfilled its primary objective and was concluded.

Ratings of subjective effects of cannabis were performed using multiple items from two different instruments: (1) the 44-item VAS^22^ and (2) the CRF^23^. Both instruments used visual analog scales (from 0 mm to 100 mm) to measure subjective effects. The primary objective was to evaluate the effects of AEF0117 on the perceived ‘good effect’ of cannabis as a measure of abuse liability, which was measured by (1) a subscale of the 44-item VAS (primary endpoint), containing the arithmetic mean of two items, ‘I feel a Good Effect’ and ‘I feel High’, and (2) two individual items of the CRF (key secondary endpoints): ‘Felt Good Cannabis Effect’ and ‘Cannabis Cigarette Liking’. The subscale of the 44-item VAS was initially named the ‘Good Cannabis Effect’ subscale. Before starting the statistical analysis, this subscale was renamed ‘Intoxication’ subscale to avoid confusion with a key secondary endpoint on the CRF labeled ‘Felt Good Cannabis Effect’.

Cognitive performance was measured by a test battery that included the Sustained Attention to Response Task (SART), Behavioral Pattern Separation (BPS-O), Digital Substitution Task (DSST) and Stroop Color task.

The Cold Pressor Test (CPT)^40^ was used to assess the analgesic effects of cannabis and was performed on day 1 only of each dosing period.

Participants stayed in the research clinic for 6 d during each dosing period. A computer-generated randomization schedule was prepared by a statistical programmer not directly involved in the conduct of the study, and the investigational product was packaged in a double-blind manner. Participants were randomly assigned within a dose-escalation cohort to one of two treatment sequences: group 1, AEF0117 during period A and placebo during period B, or group 2, AEF0117 during period B and placebo during period A.

Research participants were advised that they would receive both active and placebo study medication but were blinded as to whether they received AEF0117 or placebo during the two periods (A and B). Research staff who interacted with participants were also blinded as to AEF0117 dose, as were the principal investigator and sponsor representatives; CRO personnel were blinded to the treatment condition until database closure/finalization for that cohort. The pharmacist and his/her attendant were the only personnel to have access to the randomization list, to prepare the drug for administration, which was packaged in a double-blind manner.

On each study day (days 1–5), at approximately 12:30, participants received an experimenter-administered ‘sample’ of cannabis (two cannabis cigarettes of approximately 800 mg, 7% THC, provided by NIDA). They were guided by an investigator through smoking a total of six 5-s inhalations using a paced-puff smoking procedure, where inhalation duration, time spent holding smoke in the lungs and inter-puff interval were timed. The controlled amount of experimenter-administered cannabis captures the effects of AEF0117 on cannabis’ subjective effects and cognitive performance relative to placebo. To capture a full timecourse of cannabis effects, participants completed the 44-item VAS and a CRF at 12:50, 13:10, 13:30, 14:00 and 14:15.

Cognitive performance was measured every day 3 h before the cannabis administration at 12:30 and 1 h afterwards at 13:30 after participants had completed this timepoint of the 44-item VAS and the CRF.

On study day 1, baseline pain threshold and pain tolerance were measured using the CPT at approximately 14:45. Participants received a second experimenter-administered ‘sample’ of cannabis (six puffs using the procedures described above), and the CPT test was repeated at 30, 60, 90, 135 and 195 min after cannabis administration.

On study days 2–5, beginning at 14:30, participants were given the option to self-administer individual puffs of cannabis (up to six puffs per timepoint) every 2 h until 20:30 (maximum of 24 puffs per day). Fifteen minutes before each self-administration opportunity, participants indicated the number of puffs they chose to purchase ($2 per puff), and they would pay for it (using faux money that was then subtracted from their study stipend) before receiving their individual ashtray, cannabis cigarette and lighter.

Participants were told at study onset that cannabis strength could vary from day to day and between participants, but whatever they each smoked at 12:30 that day was what was available that day for self-administration. Thus, if the medication altered cannabis’ effects for that individual, they might think that they were receiving a lower strength of cannabis than other days or than other participants.

On day 6 of each period, before discharge from the research center, participants had sitting blood pressure and pulse rate measurements, clinical laboratory tests (chemistry, hematology and urinalysis) and body weight taken. The study start date was 23 October 2018; primary completion date was 31 July 2020; and actual study completion date was 1 January 2021. After the second study phase, participants were contacted 28 d after discharge so that we could inquire about the occurrence of any serious AEs (including pregnancies) that may have occurred since leaving the research site.

#### Assessment of precipitated withdrawal in participants with CUD

In addition to general safety assessment of vital signs, clinical chemistry and AEs, symptoms of cannabis withdrawal were assessed by daily measures of food intake, body weight, sleep efficiency^23,41,42^ and mood^22^.

##### Mood

The measure of potential negative mood states was performed using the subscales ‘Miserable’, ‘Anxious’ and ‘Irritable’ of a 44-item VAS^22^ that were assessed 30 min before and 2.5 h after AEF0117 administration (30 min before cannabis smoking).

##### Sleep

Participants wore an Actiwatch device each inpatient night that measured Sleep Onset, Sleep Efficiency, Sleep Percentage, Snooze Time, Wake Bouts and Wakefulness after Sleep Onset. Participants also completed a VAS sleep questionnaire each inpatient morning^23^ with the following items: ‘I slept well last night’, ‘I woke up early this morning’, ‘I fell asleep easily last night’, ‘I feel clear-headed this morning’, ‘I woke often last night’, ‘I was satisfied with my sleep last night’, ‘I had a lot of dreams last night’ and ‘How many hours did you sleep last night?’. Ratings were collected on day 1 before dosing with AEF0117 and 24 h from dosing with AEF0117 (day 2 to day 6, the day of discharge).

##### Caloric intake and body weight

Participants recorded the time and quantity of each food item consumed (verified by staff examining their food trash at the end of the day), and caloric intake was calculated for each day. Body weight was measured every morning from day 1 to day 6; day 6 was the day of discharge (no medication administered).

#### Assessment of negative mood symptoms while smoking cannabis

We measured seven subscales of the 44-item VAS (‘Miserable’, ‘Anxious’, ‘Tired’, ‘Confused’, ‘Irritable’, ‘Social’ and ‘Bad effect’). These measures were collected after participants smoked a controlled amount of cannabis 3.5 h after AEF0117 administration (12:30). Ratings were then done repeatedly (five times at 20, 40, 60, 90 and 120 min after cannabis).

#### Statistical analysis

For this phase 2a study, the sample size considerations were based on the expected treatment effect, and variability was extrapolated from other medication studies in participants with CUD, as the current study was the first one, to our knowledge, to assess a medication with this MOA. The sample size estimation was based on a *t*-test for paired samples under the assumption that the within-subjects correlation is 0.50 and the standard deviation within treatment period is 35 mm. Using 12 participants, a difference of 36 mm could be demonstrated with 90% power. Of note, a statistically significant effect (two-sided *P* ≤ 0.05) could be shown, should a mean difference of 21.7 mm be observed with a standard deviation of 35 mm.

An amended version of the SAP of this study was signed and filed before the database lock. The amendment aimed to clarify the secondary objectives and better define the key secondary, secondary and exploratory endpoints. Principally, this amendment stated that timecourse data should be analyzed for the primary and secondary endpoints evaluating the subjective effects of cannabis. It also added as a key secondary endpoint the percentage of particpants who self-administer over time as a function of medication dose.

As specified in the SAP, the statistical analyses employed an MMRM analysis using the REML as estimation method and structured covariance matrix with compound symmetry; the model included as factors and covariates: sequence, dose, treatment, time and days as fixed effects and subject within sequence as random effect.

Based on the SAP, the following analytical steps depending on results of the various analyses were undertaken. The primary and key secondary endpoints were first analyzed with a global crossover MMRM analysis (using combined cohorts: 0.06 mg + 1 mg). After this first analysis and because of the result:

- For the variables for which a Sequence × Treatment and/or a Sequence × Treatment × Time or Sequence × Treatment × Day were observed, the crossover analysis was stopped.
- For the variables for which a significant Treatment × Dose interaction was observed with the crossover analysis without a Treatment × Sequence interaction, then a ‘dose per dose’ analysis was performed.
- For the primary and key secondary endpoints for which an interaction between treatment and dosing sequence was observed, a parallel group MMRM analysis (period A only) over time was performed on the combined cohorts (0.06 mg + 1 mg).
- If a significant Dose × Treatment interaction was observed in the parallel group MMRM analysis, then a parallel group MMRM analysis (period A only) ‘dose per dose’ was performed.

Due to the significant interaction between dosing sequence and treatment observed in the global crossover MMRM analysis, the following complementary analyses described in the SAP and listed below were not performed:

- Analysis of baseline values before cannabis administration at 12:30.
- Analysis of peak effect after cannabis administration at 12:30 as change from baseline (11:30) before cannabis administration.
- Analysis of cannabis effects over time after cannabis administration at 12:30 as change from baseline (11:30) before cannabis administration.
- Analysis of the changes in cannabis subjective effects on day 4 and day 5.

As a significant Sequence × Treatment interaction was observed, a post hoc exploratory analysis was performed after the unblinding of the data to try to characterize the sequence effects. The data over time of the ‘Intoxication’ subscale of the 44-item VAS, the item ‘Felt Good Cannabis Effect’ of the CRF and the ‘number of puffs’ during self-administration were plotted by treatment sequence and visually inspected. The results were similar for the three variables and were best exemplified by the ‘Felt Good Cannabis Effect’ item of the CRF, which are reported here in Fig. 3i.

To choose the doses of AEF0117 to be used in the phase 2a study, we used a statistical population PK model built using the SAD and MAD PK data. Population PK analysis of AEF0117 was performed using a nonlinear mixed effects model as implemented in the Nonlinear Mixed Effects Modeling (NONMEM) computer program. The usual first-order conditional estimation (FOCE) method with interaction was used throughout. For the structural model, the NONMEM analysis subroutines ADVAN 1, ADVAN 2, ADVAN 4, ADVAN 5, ADVAN 6, ADVAN 12 and ADVAN 13 were used to test for one-, two- and three-compartment models with an oral dosing compartment. Once the structural model was defined, covariates were tested to explain inter-individual variability on PK parameters. The final mathematical model, using Monte Carlo simulations (as implemented in the NONMEM software) and the final estimated parameters, allowed us to simulate, with a high level of accuracy, plasma drug levels as a function of dose, even for doses not directly tested. In addition, this type of model includes covariates explaining individual variability of plasma concentrations, which allowed us to estimate which dose would result in 90% of the participants achieving plasma concentrations at or above a particular target.

#### PK studies in research volunteers with CUD

Blood samples were collected on day 1 of each treatment period with sampling before dose and 3 h, 9.5 h and 24 h after the first dose of AEF0117 or placebo. On day 6, another blood sample was drawn 24 h after the last (5th) dose of AEF0117 or placebo. THC and its metabolites (11-OH-THC and 11-COOH-THC) were assessed at the same timepoints. Exposure of AEF0117 was estimated using the PK dataset (*n* = 14 for the 0.06-mg dose and *n* = 15 for the 1-mg dose). THC and its metabolites analyses were performed using PD dataset (*n* = 13 for both doses).

PK parameters for AEF0117 were subject to descriptive statistics separately for each treatment group. The effect of AEF0117 on the plasma concentration of THC, 11-OH-THC and 11-COOH-THC were analyzed using a global crossover MMRM analysis.

#### Steroids and endocannabinoid analysis

Blood samples were collected on day 1 of each treatment period with sampling 30 min before (pre-dose) and 3 h, 9.5 h and 24 h after the first administration of AEF0117 or placebo. On day 6, another blood sample was drawn 24 h after the last (5th) dose of AEF0117 or placebo. ALLO, DHEA, PREG and TESTO, as well as AEA and 2-AG, were measured. Data were analyzed using a global crossover MRMM analysis on the PD dataset (*n* = 13 per dose cohort). CORT, estradiol and PROG were analyzed before dose on day 1 and 24 h after the last (5th) dose of AEF0117 or placebo (day 6) using the safety dataset, comprising all the randomized participants (*n* = 14 for the 0.06-mg dose and *n* = 15 for the 1-mg dose).

### Reporting summary

Further information on research design is available in the Nature Portfolio Reporting Summary linked to this article.

## Online content

Any methods, additional references, Nature Portfolio reporting summaries, source data, extended data, supplementary information, acknowledgements, peer review information; details of author contributions and competing interests; and statements of data and code availability are available at 10.1038/s41591-023-02381-w.

## Supplementary information

Supplementary Information Supplementary Methods and Tables 1–21.

Reporting Summary

## Data Availability

All data are available in the main text, Extended Data and Supplementary Information. For privacy reasons, individual participant data pertaining the clinical trials reported in this article will be provided, after de-identification, upon reasonable request to qualified scientific researchers who provide methodologically sound and justified research proposals. Requests could be subject to a confidential disclosure agreement or a material transfer agreement, depending on the data (for this purpose, contact the corresponding author). Access to at least the minimum data from the clinical trials and/or related documents that are necessary to carry out the proposed research will be granted within a reasonable period, which, according to the request, can range from 1 month to 3 months, and for a pre-specified amount of time and through a secure server depending on the nature of the research plan.
