# Supplementary material for: Signaling-specific inhibition of the CB1 receptor for cannabis use disorder: phase 1 and phase 2a randomized trials
Source: Nat Med. 2023 Jun 8;29(6):1487–99. doi: 10.1038/s41591-023-02381-w (PMC10287566; doi:10.1038/s41591-023-02381-w)
Supplement: Supplementary file 1 — Supplementary Methods and Tables 1–21. [file 41591_2023_2381_MOESM1_ESM.pdf]

nature medicine

Article https://doi.org/10.1038/s41591-023-02381-w

Signaling-specific inhibition of the CB_1 receptor for cannabis use disorder: phase 1 and phase 2a randomized trials

In the format provided by the authors and unedited

Pharmacokinetic results in mice rats and dogs

PK comparison in males and females. After single administration of AEF0117 in mice (0.3, 4 and 10 mg/kg, *per os*), rats (1.6, 2, 9, 20, 36 and 65 mg/kg, *per os*) or dogs (0.7, 2, 9, 20, 36 and 65 mg/kg, *per os* and 0.7 mg/kg, iv), there were no differences between males and females for the PK parameters of AEF0117 in the plasma nor in the brain (Supplementary Table 1, Extended Data Fig. 2). In the brain, sex differences were compared only in mice and data are presented as average values of males and females. Similarly, no sex differences were found in the TK studies after repeated (28- or 91-day) administration of AEF0117 (2, 9, 20, 36, and 65 mg/kg) in rats and dogs.

AUC/dose linearity. After single administration *per os* of AEF0117 (Supplementary Table 1, Extended Data Fig. 2) the increase in AUC in plasma was linear over the three doses studied (0.3, 4 and 10 mg/kg) in mice. In rats, the increase in AUC was largely linear up to 20 mg/kg and less than linear afterwards (36 and 65 mg/kg), with the highest plasmatic exposure observed after administering 65 mg/kg *per os*. Complementary experiments (data not shown) showed that above 65 mg/kg and up to 1000 mg/kg, the AUC of AEF0117 in rats progressively declined. In dogs, the increase in AUC was linear between 0.7 and 36 mg/kg (0.7, 2, 9, 20, and 36 mg/kg) and less than linear between 36 and 65 mg/kg, which indicates a saturation of absorption at this dose.

PK comparison between species. After single administration (Supplementary Table 1) of AEF0117 *per os* in mice, rats, and dogs, the t_{max} of AEF0117 was similar (~3 h), suggesting a comparable rate of AEF0117 absorption. The half-life of AEF0117 was longer in dogs (24.4-31.6 h *per os*; 33.5-38.2 h iv) than mice (10.1-25.6 h) and shortest in rats (7.3-15.8 h). AUC/dose ratios were higher in dogs (1048-2251) than

mice (750-1220) and lowest in rats (412-836). After repeated administration of AEF0117 (28 days) in rats and dogs, exposure to AEF0117 increased more in dogs than in rats (Ratio Day 28/Day 1: rats=1.9-2.9; dogs=3.6-6.9). In both species, a steady state was achieved after approximately 14 days of administration.

Bioavailability. The bioavailability of AEF0117 in dogs, calculated as the ratio of the AUC after *per os* and iv administration was approximately 68%.

Distribution in brain and plasma. In mice and rats, AEF0117 had a longer t_{max} (7 h vs 3 h) and higher AUC and C_{max} in the brain than in plasma. The AUC_{last} and C_{max} brain/plasma ratio seemed inversely related to the dose, with the highest ratios (C_{max} =2.8 and AUC= 7.3) observed for 0.3 mg/kg AEF0117. Half-life of AEF0117 in the brain and plasma were similar.

Results of the 91-day repeated oral toxicology study in rats and dogs

The findings of the 91-day repeated oral toxicity studies are reported below. The No-Observed Adverse Effect Level (NOAEL) corresponded to the highest dose tested in both rats and dogs: 65 mg/kg/day. Given that the most observed ID50 of AEF0117 for inhibiting the effects of THC in mice, rats and non-human primates is 0.005 mg/kg, AEF0117 has a therapeutic index (TI) ≥13000.

Findings in rats

In male rats, a slight increase in blood urea level was observed at the two highest doses (+13% at 20 mg) and (+10% at 65 mg) at the end of the treatment period (Week 14); this was not associated with histopathological changes in the kidneys. Moreover, blood urea levels were lower than that measured in the vehicle control group in Week 18 (end of the drug withdrawal period). Consequently, this change was not considered to have potential clinical significance.

In female rats, there was a change in thymus weight at 65 mg/kg/day at the end of the drug withdrawal period (Day122) (-17% in absolute value, -19% relative to body weight value and -18% relative to brain weight value, when compared to the vehicle group). However, there were no changes in thymus weight at any other time point or dose and no changes in lymphocyte count (cells produced by this organ). Furthermore, a similar incidence of thymic atrophy was observed in animals treated with AEF0117 and in the control group. Consequently, these changes observed in thymus weight were not likely to be related to the treatment with AEF0117.

At necropsy on Day 92, pale liver with pinpoint change was observed in most animals, including in the vehicle control group. After a 4-week recovery period (i.e., at necropsy on Day 122), this finding was no longer present. Since no microscopic abnormalities were observed in the liver of the animals sacrificed on Day 92 and since there were no changes in liver enzymes, these changes could be related to the vehicle (corn oil) after three months of daily administration and were thus not considered to be of toxicological significance.

Findings in Dogs

From Day 35 up to the end of the treatment period, the body weight gain of animals treated with AEF0117 at 20 and 65 mg/kg/day was slightly, but not statistically significantly, reduced as compared to the vehicle control group or to the group dosed with 2 mg/kg/day. At the end of the AEF0117 withdrawal period, the body weight of animals administered AEF0117 was not different from the body weight of vehicle control animals.

From week 5 of treatment, there was a lower eosinophil count in all groups administered AEF0117 (-41 at week 5 to -54% at week 14). At the end of the treatment period (week 14), there was also a lower lymphocyte count in all the

groups administered AEF0117 (-17 to -24% at week 14). These changes were still present at the end of the drug withdrawal period (week 18) in the group that was given AEF0117 (eosinophil count: -66%; lymphocyte count -19%). At the end of the treatment period, the thymus weight was slightly lower in all animals treated with AEF0117 than in the vehicle control group (-15 to -24%). This effect fully recovered: at the end of the drug withdrawal period, the thymus weight of animals administered AEF0117 (65 mg/kg/day) was similar to that of the vehicle control group.

Phase 1 SAD and MAD studies in healthy volunteers

Pharmacokinetics results

AEF0117 maximum plasma concentrations were reached at a median time to maximum concentration (t_{max}) within 2 to 3 h (Supplementary Table 18, Extended Data Fig. 2e,f). After administration of single ascending oral doses of 0.2 to 6 mg of AEF0117, a close to dose proportional increase of C_{max} and $AUC_{0\text{-}last}$ was observed, regardless of the dose range, 0.2 to 6 mg, or 0.6 to 6 mg (Supplementary Table 18). A trend for a small, but non-significant increase in peak concentrations and exposure of AEF0117 was observed between the first and the 7th oral administrations (Supplementary Table 18).

Plasma concentrations of AEF0117 decreased in 2 phases. The first phase, characterized by a rapid decrease in concentration that lasted approximately 24 h after dosing, corresponded to the distribution phase and the beginning of the elimination phase. A second phase, characterized by a much slower decrease in AEF0117 concentration, occurred 24 h after dosing (Extended Data Fig. 2f, Supplementary Table 18). In the SAD study, the second phase was only evaluated after the 0.6, 2 and 6 mg doses of AEF0117, at which time a more sensitive analytical assay was available. As the $AUC_{0\text{-}24h}$ represents between 54.7 and 74.3%

of the $AUC_{0\text{-}inf}$, results suggest that the first phase accounts for the minority of the total exposure. Consequently, the second phase did not contribute considerably to the overall drug exposure, and likely reflects the slow elimination of AEF0117 from body tissue. The terminal half-life for these cohorts was not evaluated over a sufficient interval to be fully determined. For this reason, no descriptive statistics could be computed, but the minimum and maximal values observed in different participants are shown in Supplementary Table 4. For the MAD study, the sampling period was longer (264 h) and the terminal elimination half-life ranged between participants between 152 and 259 h and concentrations at 264 h corresponded to 4% of the C_{max} observed at Day 7 for all doses.

Phase 2a in research volunteers with CUD

Pharmacokinetic analysis

Analysis of the data pooled across the two dosing schedules (AEF0117 first or placebo first) according to the two AEF0117 doses tested (0.06 and 1 mg) showed an increase as a function of repeated administration: mean AEF0117 concentrations were 30-50% higher 24 h after the 5th dose administration compared to 24 h after the 1st dose administration. There was considerable inter-individual variability, especially at 3 and 9.5 h after dose administration, which most likely indicates that the t_{max} occurred at different times among participants between these two timepoints. Plasma levels after 0.06 and 1 mg AEF0117 administration were in the range predicted from animal data (i.e., C_{max} ≥150 pg/mL and ≥1500 pg/mL, respectively) and by the PK population model developed for AEF0117 based on the PK data in healthy volunteers.

When the plasma concentration data were analyzed taking the sequence of administration (AEF0117 or placebo first) into consideration, it was apparent Fig. 3

j,k) that measurable concentrations of AEF0117 were still present after 14 days of washout, most notably after 1 mg AEF0117 (Fig. 3k). Inspection of the individual plasma concentration data for all participants showed that quantifiable plasma concentrations were seen in all participants when placebo was administered in the second dosing period if they had received 1 mg AEF0117 in the first dosing period (AEF0117 first), i.e., more than 14 days earlier. For the 0.06 mg cohort, one participant in the AEF0117 first group also had plasma levels of AEF0117 above the lower limit of quantification (LLOQ)14 days later. Further, there were detectable plasma concentrations in 3 of the remaining 5 participants in the 0.06 mg dosing group but concentrations were below the LLOQ (range 0.2 to 9.3 pg/mL, data included in Fig. 3j). These findings may be explained by the long terminal half-life of AEF0117, with relative low exposures during the terminal phase contributing to the overall exposure after multiple doses. In the first period no AEF0117 was detectable in the plasma after dosing with placebo (Fig. 3j,k).

Species	Dosing	Dose mg/kg	n	C_{max} (ng/mL)	t_{max} (h)	AUC_{last} (ng/mL*h)	AUC /dose ratio	AUC M/F ratio	t½ (h)
**Mice**	***per os***	0.3	6	35.5	2.5	251.65	838.85	1.1	17.8
		4	6	489.55	2.25	3734	933.5	1.5	18.3
		10	6	1787	2.25	11304.5	1130.45	0.9	18.2
**Rats**	***per os***	1.6	9	116.4	4.1	1058.7	661.7	0.7	13.9
		2(1)	6	106.45	4	1454	727	1.36	8
		2(2)	6	86.35	4	1207.3	603.5	1.7	7.7
		9(1)	6	577.8	3	6146.5	683	1.08	11.65
		20(2)	6	1057.5	3	12718.5	636	1.1	7.6
		36(1)	6	2064	3	16495.5	458.5	2.12	9.8
		65(2)	6	1663.2	3	18221.7	280.5	1.2	10.45
**Dogs**	**i.v.**	0.7	6	505	0.125	2192	3131	1.28	35.9**
	per os	0.7	6	137.7	2.67	1452.5	2074.5	0.93	28.0**
		2* (0.5)(1)	6	95	4	1023.5	512 (1997)	0.81	ND
		2(2)	6	241.1	4	2912.9	1460.2	0.72	ND
		9(1)	6	1195	6	18526.5	2058.5	1.12	ND
		20(2)	6	2529.5	4	26470.7	1323.5	0.79	ND
		36(1)	6	3482	4	50327.5	1398.0	0.5	ND
		65(2)	6	3304.8	4	43508.1	669.4	0.87	ND

Supplementary Table 1. Plasma pharmacokinetic parameters in mice, rats, and dogs after single AEF0117 administration. Data from males and females were averaged.
(1) data from the 28-day repeated oral toxicity study.
(2) data from the 91-day repeated oral toxicity study.
* theoretical dose: all the animals from this group were underdosed, receiving approximately 0.5 mg/kg (in brackets) during the first week of treatment. The C_{max} and AUC values should be roughly multiplied by 4 to compensate for the lower dose administered. The AUC/dose ratio in brackets is calculated using the actual dose of 0.5 mg/kg.
**= $t_{½}$ determined using a manual linear regression technique choosing the last three points of each PK curve.
ND= Not determined

Behavioral effects of THC	ID50 (µg/kg)	ID100 (µg/kg)	% Inhibition
**Behaviors inhibited by AEF0117 1.5 µg/kg dose range**			
Reinstatement of THC Self-administration*	ND	≤1.5	80
Increase in psychomotor stimulation	0.36	1.5	100
Impairment of long-term memory	ND	≤5	100
**Behaviors inhibited by AEF0117 15 µg/kg dose range**			
Self-administration of THC*	5	15	80
Self-administration of WIN 55,212-2	5	15	80
Impairment of pre-pulse inhibition	4.5	15	100
Impairment of social interaction	5	15	100
Impairment of reality testing	ND	≤15	100
Increase in food intake	15	50	100
Catalepsy	5	50	50

Pharmacodynamic effects per se	Overall Effect		Highest dose tested (mg/kg)		N fold ID50[&] or ID100[£]	
	AEF	Rimo	AEF	Rimo	AEF	Rimo
Inhibition of spontaneous food-intake**	**No**	Yes	15	10	3000	1[§]
Precipitated withdrawal in THC-dependent mice	**No**	Yes	0.15	10	30	1[§]
Increase in anxiety[#]-related behaviors**	**No**	Yes	15	10	3000	1[§]
Increase in depression[$]-related behaviors**	**No**	Yes	15	10	3000	1[§]
Increase in glucocorticoid secretion	**No**	Yes	10	10	2000	1

Supplementary Table 2. Effect of AEF0117 on cannabinoids-mediated behaviors and *per se* compared to the CB_1 orthosteric antagonist rimonabant.

ND = Not determined.

* = experiments performed in squirrel monkeys; all the other experiments were performed in mice.

** = these experiments were performed both after acute and repeated (28 days once a day) administration of AEF0117.

# = measured by the elevated plus maze.

$ = measured by the sucrose preference test.

& = for AEF0117 5 µg/kg most observed ID50 to inhibit THC behavioral effects.

£ = for rimonabant 10mg/kg the known ID100 for these effects.

§ = rimonabant and AEF groups were run in parallel.

Behaviors	Time (h)	AEF0117 (mg/kg)				Behaviors	Time (h)	AEF0117 (mg/kg)			
		0	2	9	36			0	2	9	36
Animal eats	0	0/6	0/6	0/6	0/6	Aggressiveness towards cagemates	0	0/6	0/6	0/6	0/6
	1	0/6	0/6	0/6	0/6		1	0/6	0/6	0/6	0/6
	3	0/6	0/6	0/6	0/6		3	0/6	0/6	0/6	0/6
	6	0/6	0/6	0/6	0/6		6	0/6	0/6	0/6	0/6
	8	0/6	0/6	0/6	0/6		8	0/6	0/6	0/6	0/6
	24	0/6	0/6	0/6	0/6		24	0/6	0/6	0/6	0/6
Animal drinks	0	0/6	0/6	0/6	0/6	Wet sawdust	0	0/6	0/6	0/6	0/6
	1	0/6	0/6	0/6	0/6		1	0/6	0/6	0/6	0/6
	3	0/6	0/6	0/6	0/6		3	0/6	0/6	0/6	0/6
	6	0/6	0/6	0/6	0/6		6	0/6	0/6	0/6	0/6
	8	0/6	0/6	0/6	0/6		8	0/6	0/6	0/6	0/6
	24	0/6	0/6	0/6	0/6		24	0/6	0/6	0/6	0/6
Animal sleeps	0	0/6	0/6	0/6	0/6	Vocalization (home cage)	0	0/6	0/6	0/6	0/6
	1	0/6	0/6	0/6	0/6		1	0/6	0/6	0/6	0/6
	3	0/6	0/6	0/6	1/6		3	0/6	0/6	0/6	0/6
	6	1/6	3/6	2/6	1/6		6	0/6	0/6	0/6	0/6
	8	3/6	1/6	0/6	1/6		8	0/6	0/6	0/6	0/6
	24	0/6	0/6	0/6	0/6		24	0/6	0/6	0/6	0/6
Animal moving around in the cage	0	3/6	3/6	2/6	5/6	Grooming (home cage)	0	0/6	0/6	0/6	0/6
	1	3/6	5/6	4/6	4/6		1	0/6	0/6	0/6	0/6
	3	4/6	2/6	1/6	1/6		3	0/6	0/6	0/6	0/6
	6	0/6	0/6	1/6	2/6		6	0/6	0/6	0/6	0/6
	8	0/6	2/6	1/6	1/6		8	0/6	0/6	0/6	0/6
	24	1/6	4/6	1/6	3/6		24	0/6	0/6	0/6	0/6
Piloerection (home cage)	0	0/6	0/6	0/6	0/6	Unusual behaviour (home cage)	0	0/6	0/6	0/6	0/6
	1	0/6	0/6	0/6	0/6		1	0/6	0/6	0/6	0/6
	3	0/6	0/6	0/6	0/6		3	0/6	0/6	0/6	0/6
	6	0/6	0/6	0/6	0/6		6	0/6	0/6	0/6	0/6
	8	0/6	0/6	0/6	0/6		8	0/6	0/6	0/6	0/6
	24	0/6	0/6	0/6	0/6		24	0/6	0/6	0/6	0/6

Supplementary Table 3. Irwin Test. Group incidence scores for behaviors in the home cage.
Results are expressed as the group incidence of animals showing the sign. Time=0: measurement before dose administration. For all measures $P>0.05$, when compared with the control group dosed with vehicle, Fisher's test.

Behaviors (range of scores; normal score)	Time (h)	AEF0117 (mg/kg)				Behaviors (range of scores; normal score)	Time (h)	AEF0117 (mg/kg)			
		**0**	**2**	**9**	**36**			**0**	**2**	**9**	**36**
**Arousal (0-8; 4)**	**0**	4.0	4.0	4.0	4.0	**Positional passivity (0-8; 4)**	**0**	3.0	2.0	3.0	2.0
	**1**	4.0	4.0	4.0	4.0		**1**	5.0	5.5	6.0	6.0
	**3**	4.0	4.0	4.0	4.0		**3**	6.0	6.0	5.0	6.0
	**6**	3.5	3.0	3.0	4.0		**6**	5.0	6.0	6.0	5.5
	**8**	3.0	3.0	3.0	3.0		**8**	6.0	6.0	6.0	5.5
	**24**	4.0	4.0	4.0	4.0		**24**	5.0	6.0	6.0	5.0
**Finger approach (0-8; 4)**	**0**	4.0	5.0	4.0	4.0	**Catalepsy (0-4; 0)**	**0**	0.0	0.0	0.0	0.0
	**1**	5.0	6.0	4.0	5.0		**1**	0.0	0.0	0.0	0.0
	**3**	3.0	2.0	3.0	3.0		**3**	0.0	0.0	0.0	0.0
	**6**	2.0	2.0	2.0	2.0		**6**	0.0	0.0	0.0	0.0
	**8**	2.0	2.0	2.0	2.0		**8**	0.0	0.0	0.0	0.0
	**24**	4.0	5.0	4.0	6.0		**24**	0.0	0.0	0.0	0.0
**Head touch (0-8; 4)**	**0**	4.0	4.0	4.0	4.0	**Visual placing (0-8; 4)**	**0**	4.0	4.0	4.0	4.0
	**1**	4.0	4.0	4.0	4.0		**1**	4.0	4.0	4.0	4.0
	**3**	4.0	4.0	4.0	3.0		**3**	4.0	4.0	4.0	4.0
	**6**	2.0	2.0	4.0	4.0		**6**	4.0	4.0	4.0	4.0
	**8**	2.0	4.0	4.0	2.0		**8**	4.0	4.0	4.0	4.0
	**24**	4.0	4.0	4.0	4.0		**24**	4.0	4.0	4.0	4.0
**Unusual behaviour (arena) (0-4; 0)**	**0**	0.0	0.0	0.0	0.0						
	**1**	0.0	0.0	0.0	0.0						
	**3**	0.0	0.0	0.0	0.0						
	**6**	0.0	0.0	0.0	0.0						
	**8**	0.0	0.0	0.0	0.0						
	**24**	0.0	0.0	0.0	0.0						

Supplementary Table 4. Irwin Test. Median values for awareness measures.
Results are expressed as median values of scores of the signs. Time=0: measurement before dose administration. For all measures P>0.05, when compared with the control group dosed with vehicle, non-parametric Mann-Whitney U test.

Behaviors (range of scores; normal score)	Time (h)	AEF0117 (mg/kg)				Behaviors (range of scores; normal score)	Time (h)	AEF0117 (mg/kg)			
		0	2	9	36			0	2	9	36
Fear (0-8; 4)	0	4.0	4.0	4.0	4.0	Aggressiveness/ irritability (0-4; 0)	0	0.0	0.0	0.0	0.0
	1	4.0	4.0	4.0	4.0		1	0.0	0.0	0.0	0.0
	3	4.0	4.0	4.0	4.0		3	0.0	0.0	0.0	0.0
	6	4.0	4.0	4.0	4.0		6	0.0	0.0	0.0	0.0
	8	4.0	4.0	4.0	4.0		8	0.0	0.0	0.0	0.0
	24	4.0	4.0	4.0	4.0		24	0.0	0.0	0.0	0.0
Grooming (0-4; 0)	0	0.0	0.0	0.0	0.0	Abnormal vocalization (0-4; 0)	0	0.0	0.0	0.0	0.0
	1	0.0	0.0	0.0	0.0		1	0.0	0.0	0.0	0.0
	3	0.0	0.0	0.0	0.0		3	0.0	0.0	0.0	0.0
	6	0.0	0.0	0.0	0.0		6	0.0	0.0	0.0	0.0
	8	0.0	0.0	0.0	0.0		8	0.0	0.0	0.0	0.0
	24	0.0	0.0	0.0	0.0		24	0.0	0.0	0.0	0.0

Supplementary Table 5. Irwin Test. Median values for mood measures.
Results are expressed as median values of scores of the signs. Time=0: measurement before dose administration. P>0.05, when compared with the control group dosed with vehicle, non-parametric Mann-Whitney U test.

Behaviors (range of scores; normal score)	Time (h)	AEF0117 (mg/kg)				Behaviors (range of scores; normal score)	Time (h)	AEF0117 (mg/kg)			
		0	2	9	36			0	2	9	36
Body position (0-8; 4)	0	4.0	4.0	4.0	4.0	Ataxic gait (0-4; 0)	0	0.0	0.0	0.0	0.0
	1	4.0	4.0	4.0	4.0		1	0.0	0.0	0.0	0.0
	3	4.0	4.0	4.0	4.0		3	0.0	0.0	0.0	0.0
	6	4.0	4.0	4.0	4.0		6	0.0	0.0	0.0	0.0
	8	4.0	4.0	4.0	4.0		8	0.0	0.0	0.0	0.0
	24	4.0	4.0	4.0	4.0		24	0.0	0.0	0.0	0.0
Spontaneous locomotor activity (0-8; 4)	0	4.0	4.0	4.0	4.0	Rearing (0-8; 4)	0	4.0	4.0	4.0	4.0
	1	4.0	4.0	4.0	4.0		1	4.0	4.0	4.0	4.0
	3	4.0	4.0	4.0	4.0		3	4.0	4.0	4.0	4.0
	6	3.0	3.0	3.0	3.0		6	2.0	2.0	2.0	1.0
	8	3.0	3.0	3.0	3.0		8	2.0	2.0	3.0	1.0
	24	4.0	4.0	4.0	4.0		24	4.0	4.0	3.0	4.0

Supplementary Table 6. Irwin Test. Median values for measures of motor activity/coordination.

Results are expressed as median values of scores of the signs. Time=0: measurement before dose administration. For all measures P>0.05, when compared with the control group dosed with vehicle, non-parametric Mann-Whitney U test.

Behaviors (range of scores; normal score)	Time (h)	AEF0117 (mg/kg)				Behaviors (range of scores; normal score)	Time (h)	AEF0117 (mg/kg)			
		0	2	9	36			0	2	9	36
Twitches (0-4; 0)	0	0.0	0.0	0.0	0.0	Startle response (0-8; 4)	0	4.0	4.0	4.0	4.0
	1	0.0	0.0	0.0	0.0		1	4.0	4.0	4.0	4.0
	3	0.0	0.0	0.0	0.0		3	4.0	4.0	4.0	4.0
	6	0.0	0.0	0.0	0.0		6	4.0	4.0	4.0	4.0
	8	0.0	0.0	0.0	0.0		8	4.0	4.0	4.0	4.0
	24	0.0	0.0	0.0	0.0		24	4.0	4.0	4.0	4.0
Seizures (0-4; 0)	0	0.0	0.0	0.0	0.0	Sensitivity to pinching the tail (0-8; 4)	0	4.0	4.0	4.0	4.0
	1	0.0	0.0	0.0	0.0		1	4.0	4.0	4.0	4.0
	3	0.0	0.0	0.0	0.0		3	4.0	4.0	4.0	4.0
	6	0.0	0.0	0.0	0.0		6	4.0	4.0	4.0	4.0
	8	0.0	0.0	0.0	0.0		8	4.0	4.0	4.0	4.0
	24	0.0	0.0	0.0	0.0		24	4.0	4.0	4.0	4.0
Tremors (0-4; 0)	0	0.0	0.0	0.0	0.0						
	1	0.0	0.0	0.0	0.0						
	3	0.0	0.0	0.0	0.0						
	6	0.0	0.0	0.0	0.0						
	8	0.0	0.0	0.0	0.0						
	24	0.0	0.0	0.0	0.0						

Supplementary Table 7. Irwin Test. Median values for measures of central excitation. Results are expressed as median values of scores of the signs. Time=0: measurement before dose administration. P>0.05, when compared with the control group dosed with vehicle, non-parametric Mann-Whitney U test.

MUSCLE TONE						REFLEXES					
Behavior (range of scores; normal score)	Time (h)	AEF0117 (mg/kg)				Behavior (range of scores; normal score)	Time (h)	AEF0117 (mg/kg)			
		0	2	9	36			0	2	9	36
Hypotonic gait (0-4; 0)	0	0.0	0.0	0.0	0.0	Corneal reflex (0-8; 4)	0	4.0	4.0	4.0	4.0
	1	0.0	0.0	0.0	0.0		1	4.0	4.0	4.0	4.0
	3	0.0	0.0	0.0	0.0		3	4.0	4.0	4.0	4.0
	6	0.0	0.0	0.0	0.0		6	4.0	4.0	4.0	4.0
	8	0.0	0.0	0.0	0.0		8	4.0	4.0	4.0	4.0
	24	0.0	0.0	0.0	0.0		24	4.0	4.0	4.0	4.0
Grip strength (0-8; 4)	0	4.0	4.0	4.0	4.0	Pinna reflex (0-8; 4)	0	4.0	4.0	4.0	4.0
	1	4.0	4.0	4.0	4.0		1	4.0	4.0	4.0	4.0
	3	4.0	4.0	4.0	4.0		3	4.0	4.0	4.0	4.0
	6	4.0	4.0	4.0	4.0		6	4.0	4.0	4.0	4.0
	8	4.0	4.0	4.0	4.0		8	4.0	4.0	4.0	4.0
	24	4.0	4.0	4.0	4.0		24	4.0	4.0	4.0	4.0
Body tone (0-8; 4)	0	4.0	4.0	4.0	4.0	Hind limb reflex (0-8; 4)	0	4.0	4.0	4.0	4.0
	1	4.0	4.0	4.0	4.0		1	4.0	4.0	4.0	4.0
	3	4.0	4.0	4.0	4.0		3	4.0	4.0	4.0	4.0
	6	4.0	4.0	4.0	4.0		6	4.0	4.0	4.0	4.0
	8	4.0	4.0	4.0	4.0		8	4.0	4.0	4.0	4.0
	24	4.0	4.0	4.0	4.0		24	4.0	4.0	4.0	4.0
Abdominal tone (0-8; 4)	0	4.0	4.0	4.0	4.0	Righting reflex on the ground (0-4; 0)	0	0.0	0.0	0.0	0.0
	1	4.0	4.0	4.0	4.0		1	0.0	0.0	0.0	0.0
	3	4.0	4.0	4.0	4.0		3	0.0	0.0	0.0	0.0
	6	4.0	4.0	4.0	4.0		6	0.0	0.0	0.0	0.0
	8	4.0	4.0	4.0	4.0		8	0.0	0.0	0.0	0.0
	24	4.0	4.0	4.0	4.0		24	0.0	0.0	0.0	0.0
Limb tone (0-8; 4)	0	4.0	4.0	4.0	4.0	Air righting reflex (0-4; 0)	0	0.0	0.0	0.0	0.0
	1	4.0	4.0	4.0	4.0		1	0.0	0.0	0.0	0.0
	3	4.0	4.0	4.0	4.0		3	0.0	0.0	0.0	0.0
	6	4.0	4.0	4.0	4.0		6	0.0	0.0	0.0	0.0
	8	4.0	4.0	4.0	4.0		8	0.0	0.0	0.0	0.0
	24	4.0	4.0	4.0	4.0		24	0.0	0.0	0.0	0.0

Supplementary Table 8. Irwin Test. Median values for measures of muscle tone and reflexes.

Results are expressed as median values of scores of the signs. Time=0: measurement before dose administration. P>0.05, when compared with the control group dosed with vehicle, non-parametric Mann-Whitney U test.

Family	Assay	% Inhib[1]		Family	Assay	% Inhib[1]		Family	Assay	% Inhib[1]	
		Preg	AEF 0117			Preg	AEF 0117			Preg	AEF 0117
**GPCRs**								**Ion channels**			
Adenosine	A1	-25	14	Muscarinic	M1	-14	-14	Calcium channels	Ca^{2+}-L	-19	-1
	A2A	-1	-3		M2	-4	-3	Potassium channels	KV	-23	-5
	A3	11	-20		M3	2	-18		SKCa	-8	12
Adrenergic	alpha1	1	-5		M4	-4	0	Sodium channels	Na^{+}-site2	23	5
	alpha2	-2	7		M5	8	-5	Purinergic channels	P2X	13	-29
	beta2	-5	4	Neurokinin	NK1	17	-18	Serotonin	5-HT3	6	-7
	beta1	4	3		NK2	-1	-28	**Transporters**			
Angiotensin-II	AT1	-28	7		NK3	11	-1	Dopamine	DAT	-12	0
	AT2	-18	-8	Neuropeptide Y	Y1	-11	-9	Norepinephrine	NET	-12	-5
Bombesin	BB	-18	12		Y2	-9	-22	Serotonin	SET	1	8
Bradykinin	B2	-9	-1	Opioid and opioid-like	delta2 (DOP)	9	-4	**Other receptors**			
Calcitonin gene-related peptide	CGRP	-12	-21		kappa (KOP)	38	1	Mitochondrial function	PBR	42	4
Cannabinoid	CB_1	10	11		mu (MOP)	-5	-10	Cell surfaces	TNF-alpha	4	-5
	CB_2	-19	-2		NOP (ORL1)	2	-7	GABA	GABA	10	9
Chemokines	CXCR2 (IL-8B)	-10	-1	Neurotensin	NTS1 (NT1)	-19	-14		BZD (central)	-43	1
	CCR1	-10	5	Prostanoid	EP2	24	27		Cl- channel	29	-4
Cholecystokinin	CCK1 (CCKA)	-30	-19		EP4	21	10	Glutamate	PCP	5	2
	CCK2 (CCKB)	-38	-35		IP (PGI2)	-12	-4	Kinase	PDGF	-15	-8
Dopamine	D1	-8	-12	Purinergic	P2Y	-3	16	Sigma	sigma	26	-4
	D2S	5	-1	Somatostatin	sst	-8	-4	**Nuclear receptors**			
	D3	11	2	Vasoactive intestinal peptide	VPAC1 (VIP1)	-10	-4	Steroid nuclear receptors	GR	88	22
	D4.4	20	-7		PAC1 - PACAP	-25	4		AR	95	20
	D5	-4	-2	Vasopressin	V1a	10	0		ER	26	31
Endothelin	ETA	-3	-4	Serotonin	5-HT1A	12	-11		PR	87	29
	ETB	4	2		5-HT1B	-28	-15	Non-steroid nuclear receptors	PPAR gamma	4	7
Galanin	GAL1	-16	-4		5-HT2A	-1	2		PXR	15	7
	GAL2	1	-9		5-HT2B	-38	-15				
Histamine	H1	0	4		5-HT2C	-9	4				
	H2	9	3		5-HT5a	11	1				
Melanocortin	MC4	-15	-8		5-HT6	5	-2				
Melatonin	MT1 (ML1A)	26	-11		5-HT7	11	-1				

Supplementary Table 9. Binding specificity of AEF0117 and pregnenolone to 85 receptors.

[1] Percent inhibition of control specific binding.

Time point	Placebo	AEF0117			
		0.2 mg	0.6 mg	2 mg	6 mg
	Mean ± SD	Mean ± SD	Mean ± SD	Mean ± SD	Mean ± SD
**Alertness (0 to 100 mm)**					
Baseline	89.4 ± 13.4	87.7 ± 13.6	89.6 ± 9.8	88.8 ± 12.7	92.8 ± 5.4
Day 1 - T_{max}	85.3 ± 15.9	88.6 ± 12.1	92.3 ± 5.6	85.9 ± 16.2	93.1 ± 7.5
Day 2 -H24:00	82.4 ± 16.4	89.6 ± 11.7	92.0 ± 6.8	86.3 ± 16.9	95.7 ± 4.2
**Contentedness (well-being) (0 to 100 mm)**					
Baseline	88.6 ± 13.8	88.9 ± 15.7	84.9 ± 8.5	82.4 ± 12.9	89.7 ± 15.7
Day 1 - T_{max}	85.6 ± 16.3	89.6 ± 14.7	88.4 ± 8.3	85.2 ± 13.4	89.0 ± 15.6
Day 2 -H24:00	81.4 ± 16.5	89.6 ± 14.3	91.7 ± 7.8	86.8 ± 12.3	91.8 ± 7.1
**Calmness (well-being) (0 to 100 mm)**					
Baseline	86.8 ± 18.0	88.2 ± 17.0	90.3 ± 9.8	88.0 ± 12.4	96.9 ± 5.3
Day 1 - T_{max}	82.7 ± 16.7	81.3 ± 22.7	84.2 ± 17.5	86.0 ± 14.7	93.8 ± 12.7
Day 2 -H24:00	73.8 ± 21.0	79.5 ± 21.2	85.7 ± 16.8	82.8 ± 17.8	98.6 ± 1.4

Supplementary Table 10. "Bond and Lader VAS" psychometrics test in the SAD study. Summary descriptive statistics. No clinically relevant differences between groups were observed in any of the three scales.

Time point	Placebo	AEF0117		
		0.6 mg	2 mg	6 mg
	Mean ± SD	Mean ± SD	Mean ± SD	Mean ± SD
**Alertness (0 to 100 mm)**				
Baseline	82.8 ± 13.4	69.8 ± 13.7	91.4 ± 5.6	92.3 ± 6.6
Day 1 - T_{max}	77.1 ± 14.2	69.9 ± 16.6	92.7 ± 7.3	83.3 ± 22.5
Day 2 - H24:00	79.1 ± 20.3	73.0 ± 16.3	100.0 ± 0.0	82.0 ± 19.0
Day 7 - H00:00	86.5 ± 20.1	72.7 ± 16.9	82.2 ± 30.8	90.6 ± 8.8
Day 7 - T_{max}	82.9 ± 18.6	70.9 ± 21.3	85.2 ± 24.0	87.5 ± 14.5
Day 8 - H24:00	82.4 ± 19.1	73.4 ± 16.1	88.0 ± 18.1	73.8 ± 25.0
**Contentedness (well-being) (0 to 100 mm)**				
Baseline	84.0 ± 17.0	68.1 ± 16.1	88.9 ± 10.3	88.1 ± 7.3
Day 1 - T_{max}	79.6 ± 20.3	75.4 ± 14.4	93.2 ± 6.2	77.9 ± 15.2
Day 2 - H24:00	70.8 ± 21.6	70.4 ± 19.6	98.8 ± 0.0	79.2 ± 15.8
Day 7 - H00:00	83.4 ± 21.0	74.2 ± 16.4	80.8 ± 21.6	71.4 ± 32.3
Day 7 - T_{max}	81.2 ± 21.5	69.3 ± 18.1	82.0 ± 21.9	83.9 ± 11.6
Day 8 - H24:00	76.5 ± 18.8	75.0 ± 16.6	82.1 ± 17.8	65.8 ± 30.1
**Calmness (0 to 100 mm)**				
Baseline	85.3 ± 20.2	69.3 ± 14.4	87.6 ± 16.9	76.9 ± 21.1
Day 1 - T_{max}	81.8 ± 24.5	74.7 ± 13.5	84.0 ± 20.8	78.2 ± 20.7
Day 2 - H24:00	78.0 ± 19.6	67.3 ± 16.7	100.0 ± 0.0	79.6 ± 19.0
Day 7 - H00:00	80.9 ± 27.8	71.3 ± 16.5	77.4 ± 24.6	55.5 ± 30.7
Day 7 - T_{max}	76.3 ± 24.5	68.4 ± 17.4	85.5 ± 20.2	82.7 ± 17.9
Day 8 - H24:00	67.5 ± 23.1	68.7 ± 18.0	89.3 ± 12.2	67.6 ± 28.1

Supplementary Table 11. “Bond and Lader VAS” psychometrics test in the MAD study. Summary descriptive statistics. No relevant differences between groups were observed in any of the three scales.

Time point	Placebo	AEF0117			
		0.2 mg	0.6 mg	2 mg	6 mg
	Mean ± SD	Mean ± SD	Mean ± SD	Mean ± SD	Mean ± SD
**Total Mood Disturbance (-36 to 200)**					
Baseline	-16.4 ± 13.6	-17.5 ± 4.6	-8.2 ± 14.6	-12.2 ± 13.2	-7.2 ± 13.5
Day 1 - T_{max}	-15.4 ± 13.6	-16.7 ± 7.0	-6.8 ± 12.8	-9.6 ± 14.7	-7.5 ± 8.3
Day 2 -H24:00	-18.8 ± 12.4	-18.2 ± 5.6	-6.8 ± 13.8	-10.7 ± 14.2	-8.4 ± 6.7
**Anger - Hostility (11 items, max. score = 44)**					
Baseline	0.3 ± 0.9	0.5 ± 0.8	3.8 ± 6.3	0.8 ± 1.2	0.8 ± 1.3
Day 1 - T_{max}	0.2 ± 0.4	0.3 ± 0.8	3.0 ± 5.0	0.8 ± 1.1	0.7 ± 1.6
Day 2 - H24:00	0.2 ± 0.6	0.3 ± 0.8	1.0 ± 1.7	0.8 ± 1.2	0.0 ± 0.0
**Confusion - Bewilderment (10 items, max. score = 40)**					
Baseline	1.1 ± 1.7	0.7 ± 0.5	2.3 ± 1.4	2.1 ± 2.0	2.5 ± 2.1
Day 1 - T_{max}	1.6 ± 1.8	0.8 ± 0.8	2.0 ± 1.3	2.0 ± 2.2	2.8 ± 1.0
Day 2 - H24:00	1.1 ± 1.5	1.0 ± 0.9	2.5 ± 1.9	2.1 ± 2.1	2.8 ± 2.0
**Depression - Dejection (13 items, max. score= 52)**					
Baseline	0.0 ± 0.0	0.2 ± 0.4	1.5 ± 2.5	0.4 ± 0.9	1.2 ± 2.9
Day 1 - T_{max}	0.0 ± 0.0	0.2 ± 0.4	1.3 ± 2.4	0.7 ± 1.0	0.0 ± 0.0
Day 2 - H24:00	0.0 ± 0.0	0.0 ± 0.0	1.0 ± 1.5	0.4 ± 0.9	0.2 ± 0.4
**Fatigue - Inertia (6 items, max. score = 24)**					
Baseline	0.4 ± 0.8	0.2 ± 0.4	2.3 ± 3.7	1.5 ± 3.5	0.7 ± 1.6
Day 1 - T_{max}	1.4 ± 1.9	0.3 ± 0.8	1.3 ± 2.8	1.8 ± 3.7	0.3 ± 0.8
Day 2 - H24:00	0.1 ± 0.3	0.0 ± 0.0	1.8 ± 3.3	1.3 ± 3.7	0.6 ± 0.9
**Tension - Anxiety (10 items, max. score = 40)**					
Baseline	2.2 ± 2.3	1.2 ± 1.2	2.0 ± 2.4	2.0 ± 2.0	2.5 ± 2.1
Day 1 - T_{max}	1.8 ± 2.1	1.0 ± 0.6	2.3 ± 2.3	2.6 ± 2.2	2.3 ± 2.1
Day 2 - H24:00	0.9 ± 1.0	1.2 ± 1.2	3.2 ± 3.5	2.0 ± 1.7	2.6 ± 1.5
**Vigor - Activity (9 items, max. score = 36)**					
Baseline	20.4 ± 9.7	20.2 ± 3.7	20.2 ± 4.1	18.9 ± 7.0	14.8 ± 6.9
Day 1 - T_{max}	20.4 ± 9.8	19.3 ± 5.3	16.8 ± 4.1	17.3 ± 7.3	13.7 ± 6.2
Day 2 - H24:00	21.1 ± 9.9	20.7 ± 4.2	16.3 ± 5.4	17.3 ± 7.4	14.6 ± 5.3
**Friendliness (6 items, max. score = 24)**					
Baseline	19.9 ± 8.3	18.8 ± 3.7	15.3 ± 5.9	19.6 ± 5.6	15.5 ± 6.3
Day 1 - T_{max}	18.6 ± 7.7	17.3 ± 4.5	14.7 ± 5.2	17.5 ± 4.6	15.0 ± 6.1
Day 2 - H24:00	18.7 ± 8.1	18.0 ± 4.7	13.8 ± 6.3	17.8 ± 4.8	17.4 ± 6.0

Supplementary Table 12. "POMS 65" psychometric test in the SAD study.
Summary descriptive statistics for the POMS sub-scores. Each item of the subscales has a score from 0 to 4. The total mood disturbance score (higher scores indicate a greater degree of mood disturbance) is calculated adding the scores of the five subscales anger-hostility, confusion-bewilderment, depression-dejection, fatigue-inertia, tension-anxiety and subtracting the score of the vigor-activity subscale. No relevant trend was observed in any of the eight sub-scales.

Time point	Placebo	AEF0117		
		0.6 mg	2 mg	6 mg
	Mean ± SD	Mean ± SD	Mean ± SD	Mean ± SD
**Total Mood Disturbance (-36 to 200)**				
Baseline	-12.7 ± 9.1	-7.6 ± 8.8	-8.2 ± 5.9	-12.0 ± 22.7
Day 1 - T_{max}	-7.7 ± 7.2	-4.4 ± 10.3	-10.5 ± 7.5	-5.7 ± 28.0
Day 2 - H24:00	-6.6 ± 3.7	-5.8 ± 9.7	-9.0 ± 0.0	-1.2 ± 24.2
Day 7 - H00:00	-12.8 ± 11.8	-7.0 ± 11.5	-0.2 ± 36.5	-7.0 ± 45.5
Day 7 - T_{max}	-10.7 ± 11.7	-1.6 ± 16.9	-7.0 ± 20.8	-8.5 ± 13.9
Day 8 - H24:00	-9.3 ± 9.9	-4.6 ± 16.0	-11.0 ± 9.9	39.8 ± 76.9
**Anger - Hostility (11 items, max. score = 44)**				
Baseline	0.0 ± 0.0	0.2 ± 0.4	0.5 ± 1.2	1.7 ± 4.1
Day 1 - T_{max}	0.2 ± 0.4	0.8 ± 1.1	0.5 ± 1.2	1.8 ± 4.5
Day 2 - H24:00	1.8 ± 4.0	1.0 ± 2.2	0.0 ± 0.0	3.0 ± 5.6
Day 7 - H00:00	0.2 ± 0.4	1.2 ± 2.7	3.2 ± 6.4	5.3 ± 10.8
Day 7 - T_{max}	0.7 ± 1.6	0.8 ± 1.8	1.2 ± 1.5	1.5 ± 3.2
Day 8 - H24:00	0.7 ± 1.6	1.8 ± 4.0	0.7 ± 1.2	14.2 ± 21.0
**Confusion - Bewilderment (10 items, max. score = 40)**				
Baseline	1.5 ± 1.0	2.2 ± 1.8	2.3 ± 1.4	2.0 ± 2.4
Day 1 - T_{max}	1.5 ± 1.5	2.2 ± 1.1	2.0 ± 2.1	2.5 ± 3.2
Day 2 - H24:00	1.6 ± 0.5	2.6 ± 1.1	3.0 ± 0.0	2.3 ± 2.1
Day 7 - H00:00	2.0 ± 1.8	2.4 ± 1.3	3.5 ± 5.7	3.3 ± 5.8
Day 7 - T_{max}	1.0 ± 1.1	2.6 ± 1.5	2.5 ± 3.3	2.0 ± 2.3
Day 8 - H24:00	1.8 ± 1.6	2.2 ± 1.1	1.7 ± 1.2	7.7 ± 9.7
**Depression - Dejection (13 items, max. score= 52)**				
Baseline	1.0 ± 1.5	0.4 ± 0.9	1.5 ± 1.6	1.0 ± 1.7
Day 1 - T_{max}	0.8 ± 1.6	0.0 ± 0.0	0.3 ± 0.5	2.7 ± 6.1
Day 2 - H24:00	1.0 ± 1.7	0.8 ± 1.1	0.0 ± 0.0	2.5 ± 4.5
Day 7 - H00:00	0.0 ± 0.0	0.4 ± 0.9	3.0 ± 6.0	6.3 ± 10.6
Day 7 - T_{max}	0.2 ± 0.4	1.0 ± 1.4	1.2 ± 2.9	1.5 ± 2.5
Day 8 - H24:00	1.0 ± 1.5	0.8 ± 1.8	0.2 ± 0.4	16.5 ± 26.1
**Fatigue - Inertia (6 items, max. score = 24)**				
Baseline	0.7 ± 1.6	1.0 ± 1.7	0.0 ± 0.0	1.5 ± 3.7
Day 1 - T_{max}	1.8 ± 2.9	2.6 ± 2.6	0.2 ± 0.4	1.5 ± 2.5
Day 2 - H24:00	1.6 ± 2.1	0.4 ± 0.5	0.0 ± 0.0	2.2 ± 3.4
Day 7 - H00:00	0.7 ± 1.6	1.0 ± 1.7	3.0 ± 7.3	2.7 ± 4.5
Day 7 - T_{max}	1.0 ± 1.5	3.0 ± 4.2	1.7 ± 4.1	1.5 ± 1.8
Day 8 - H24:00	0.8 ± 1.3	0.8 ± 1.3	0.7 ± 0.8	8.2 ± 11.7

Time point	Placebo	AEF0117		
		0.6 mg	2 mg	6 mg
	Mean ± SD	Mean ± SD	Mean ± SD	Mean ± SD
**Tension - Anxiety (10 items, max. score = 40)**				
Baseline	2.2 ± 1.5	2.8 ± 1.6	4.5 ± 5.7	1.7 ± 2.9
Day 1 - T_{max}	2.2 ± 1.2	3.6 ± 2.3	2.7 ± 1.6	2.8 ± 3.0
Day 2 - H24:00	1.6 ± 0.5	2.8 ± 1.5	1.0 ± 0.0	3.3 ± 3.1
Day 7 - H00:00	1.7 ± 1.6	2.6 ± 1.5	3.5 ± 3.4	6.8 ± 10.4
Day 7 - T_{max}	2.5 ± 2.3	3.6 ± 2.2	3.0 ± 2.2	2.8 ± 2.9
Day 8 - H24:00	2.7 ± 1.8	3.2 ± 3.3	2.3 ± 1.4	10.0 ± 13.6
**Vigor - Activity (9 items, max. score = 36)**				
Baseline	18.0 ± 7.5	14.2 ± 4.5	17.0 ± 5.3	19.8 ± 9.7
Day 1 - T_{max}	14.2 ± 7.6	13.6 ± 4.8	16.2 ± 5.8	17.0 ± 10.8
Day 2 - H24:00	14.2 ± 6.3	13.4 ± 5.4	13.0 ± 0.0	14.5 ± 9.6
Day 7 - H00:00	17.3 ± 9.3	14.6 ± 5.0	16.3 ± 8.2	17.5 ± 7.1
Day 7 - T_{max}	16.0 ± 9.3	12.6 ± 8.6	16.5 ± 8.0	17.8 ± 8.0
Day 8 - H24:00	16.3 ± 10.5	13.4 ± 6.2	16.5 ± 7.9	16.7 ± 10.3
**Friendliness (6 items, max. score = 24)**				
Baseline	18.7 ± 6.4	13.2 ± 4.3	16.8 ± 4.1	18.7 ± 5.6
Day 1 - T_{max}	17.8 ± 7.2	13.0 ± 5.6	18.0 ± 4.8	14.7 ± 7.0
Day 2 - H24:00	17.0 ± 4.9	13.2 ± 5.8	11.0 ± 0.0	14.8 ± 4.7
Day 7 - H00:00	18.0 ± 9.0	13.2 ± 6.0	17.7 ± 7.4	17.7 ± 5.9
Day 7 - T_{max}	17.7 ± 7.4	12.4 ± 5.0	15.7 ± 7.6	14.0 ± 4.9
Day 8 - H24:00	16.2 ± 7.1	12.0 ± 7.4	18.3 ± 6.5	14.3 ± 8.7

Supplementary Table 13. "POMS 65" psychometric test in the MAD study.
Summary descriptive statistics for the POMS sub-scores. Each item of the subscales has a score from 0 to 4. The total mood disturbance score (higher scores indicate a greater degree of mood disturbance) is calculated adding the scores of the five subscales anger-hostility, confusion-bewilderment, depression-dejection, fatigue-inertia, tension-anxiety and subtracting the score of the vigor-activity subscale. An outlier was observed in the AEF0117 6 mg group: one subject at the Day8-H24 timepoint (the day of discharge) scored 4 (extremely) to all 63 individual items of the POMS (positive and negative measures), which is consistent with a non-compliant participant; this led to very high derived scores and variance for this single timepoint. The performance of this participant however appeared normal at the other timepoints. With the exception of this participant, no dose related trend was observed in any of the derived sub-scores.

Event	Placebo			AEF0117											
				0.2 mg			0.6 mg			2 mg			6 mg		
	n (%)			n (%)			n (%)			n (%)			n (%)		
	S	D1	D2	S	D1	D2	S	D1	D2	S	D1	D2	S	D1	D2
**Suicidal ideation**	0	0	0	0	0	0	0	0	0	0	0	0	0	0	0
Wish to be Dead	0	0	0	0	0	0	0	0	0	0	0	0	0	0	0
Non-Specific Suicidal Thought	0	0	0	0	0	0	0	0	0	0	0	0	0	0	0
**Suicidal behavior**	0	0	0	0	0	0	0	0	0	0	0	0	0	0	0
Actual Attempt	0	0	0	0	0	0	0	0	0	0	0	0	0	0	0
Interrupted Attempt	0	0	0	0	0	0	0	0	0	0	0	0	0	0	0
Aborted Attempt	0	0	0	0	0	0	0	0	0	0	0	0	0	0	0
Preparatory Acts/Behavior	0	0	0	0	0	0	0	0	0	0	0	0	0	0	0
Suicidal Behavior	0	0	0	0	0	0	0	0	0	0	0	0	0	0	0
Suicide	N/A	0	0	N/A	0	0	N/A	0	0	N/A	0	0	N/A	0	0
**Suicidal ideation or behavior**	0	0	0	0	0	0	0	0	0	0	0	0	0	0	0
Non-suicidal Self-injurious Behavior	0	0	0	0	0	0	0	0	0	0	0	0	0	0	0

Supplementary Table 14. "Columbia-Suicide Severity Rating Scale (C-SSRS) test" in the SAD study.
Number of subjects with suicidal ideation, suicidal behavior and self-injurious behavior without suicidal intent. S: Screening. D1: Day 1. D2: Day2.

Event	Placebo			AEF0117								
				0.6 mg			2 mg			6 mg		
	n (%)			n (%)			n (%)			n (%)		
	S	D1	D8	S	D1	D8	S	D1	D8	S	D1	D8
**Suicidal ideation**	0	0	0	0	0	0	0	0	0	0	0	0
Wish to be Dead	0	0	0	0	0	0	0	0	0	0	0	0
Non-Specific Suicidal Thought	0	0	0	0	0	0	0	0	0	0	0	0
**Suicidal behavior**	0	0	0	0	0	0	0	0	0	0	0	0
Actual Attempt	0	0	0	0	0	0	0	0	0	0	0	0
Interrupted Attempt	0	0	0	0	0	0	0	0	0	0	0	0
Aborted Attempt	0	0	0	0	0	0	0	0	0	0	0	0
Preparatory Acts/Behavior	0	0	0	0	0	0	0	0	0	0	0	0
Suicidal Behavior	0	0	0	0	0	0	0	0	0	0	0	0
Suicide	N/A	0	0	N/A	0	0	N/A	0	0	N/A	0	0
**Suicidal ideation or behavior**	0	0	0	0	0	0	0	0	0	0	0	0
Non-suicidal Self-injurious Behavior	0	0	0	0	0	0	0	0	0	0	0	0

Supplementary Table 15. “Columbia-Suicide Severity Rating Scale (C-SSRS) test” in the MAD study.
Number of subjects with suicidal ideation, suicidal behavior and self-injurious behavior without suicidal intent. S: Screening. D1: Day 1. D8: Day8.

Time point	Placebo	AEF0117			
		0.2 mg	0.6 mg	2 mg	6 mg
	Mean ± SD	Mean ± SD	Mean ± SD	Mean ± SD	Mean ± SD
**Amphetamine group (A) scale (mm)**					
Baseline	4.4 ± 2.2	4.7 ± 1.2	4.7 ± 2.4	4.3 ± 2.2	3.5 ± 1.9
Day 1 - T_{max}	4.4 ± 2.8	4.5 ± 2.1	3.8 ± 2.6	4.8 ± 2.6	3.5 ± 1.2
Day 2 -H24:00	4.3 ± 2.5	3.8 ± 2.3	4.3 ± 2.3	4.7 ± 3.0	2.8 ± 1.5
**Benzedrine group (BG) scale (mm)**					
Baseline	3.6 ± 2.0	3.8 ± 2.3	3.5 ± 1.4	3.1 ± 3.0	2.0 ± 1.7
Day 1 - T_{max}	3.7 ± 2.3	3.5 ± 2.1	3.0 ± 1.8	3.3 ± 2.9	1.8 ± 1.2
Day 2 -H24:00	3.1 ± 2.0	3.3 ± 2.3	3.0 ± 2.0	3.8 ± 3.1	1.4 ± 1.3
**LSD group scale (mm)**					
Baseline	-2.0 ± 0.7	-2.3 ± 0.5	-1.8 ± 1.3	-2.0 ± 1.0	-1.8 ± 0.8
Day 1 - T_{max}	-1.9 ± 1.2	-2.2 ± 0.8	-1.3 ± 1.2	-1.8 ± 0.9	-2.3 ± 0.5
Day 2 -H24:00	-1.9 ± 1.1	-2.2 ± 0.8	-1.0 ± 1.4	-2.3 ± 1.1	-2.0 ± 0.7
**Morphine Benzedrine Group (MBG) Scale (mm)**					
Baseline	7.2 ± 4.4	5.7 ± 4.1	8.0 ± 5.1	6.6 ± 4.6	5.0 ± 3.4
Day 1 - T_{max}	6.5 ± 4.8	5.8 ± 5.1	6.5 ± 4.4	7.2 ± 5.3	5.2 ± 4.1
Day 2 -H24:00	6.8 ± 4.8	5.5 ± 4.9	6.5 ± 4.4	7.7 ± 5.0	5.0 ± 3.5
**Pentobarbital Chlorpromazine Alcohol Group (PCAG) scale (mm)**					
Baseline	-2.7 ± 0.7	-2.7 ± 0.5	-2.3 ± 0.8	-1.1 ± 3.1	-2.5 ± 0.8
Day 1 - T_{max}	-2.4 ± 1.3	-2.5 ± 1.2	-2.5 ± 0.8	-1.1 ± 3.1	-2.3 ± 0.8
Day 2 -H24:00	-2.6 ± 1.1	-2.7 ± 0.8	-2.3 ± 1.2	-1.3 ± 3.0	-1.8 ± 1.6

Supplementary Table 16. “ARCI 49” psychometrics test in the SAD study.
Summary descriptive statistics for the ARCI 49 sub-scores: No relevant trend was observed in any of the five sub-scores.

Time point	Placebo	AEF0117		
		0.6 mg	2 mg	6 mg
	Mean ± SD	Mean ± SD	Mean ± SD	Mean ± SD
**Amphetamine group (A) scale (mm)**				
Baseline	3.5 ± 2.4	4.8 ± 2.2	5.7 ± 1.9	4.2 ± 1.6
Day 1 - T_{max}	4.0 ± 2.1	4.0 ± 1.7	5.3 ± 2.1	2.8 ± 2.0
Day 2 - H24:00	2.2 ± 2.5	3.8 ± 2.3	2.0 ± 0.0	2.5 ± 1.8
Day 7 - H00:00	3.5 ± 2.7	3.2 ± 1.5	4.7 ± 2.2	3.7 ± 2.3
Day 7 - T_{max}	3.3 ± 2.5	3.6 ± 1.1	4.7 ± 1.8	3.7 ± 2.9
Day 8 - H24:00	3.2 ± 2.0	2.8 ± 0.8	5.0 ± 1.9	4.7 ± 3.9
**Benzedrine group (BG) scale (mm)**				
Baseline	2.5 ± 1.6	3.4 ± 1.1	4.3 ± 2.7	3.2 ± 2.1
Day 1 - T_{max}	3.5 ± 1.6	2.6 ± 1.1	4.3 ± 2.7	2.5 ± 2.2
Day 2 - H24:00	2.4 ± 2.5	1.8 ± 1.5	1.0 ± 0.0	1.5 ± 2.0
Day 7 - H00:00	2.8 ± 1.9	2.4 ± 2.1	2.8 ± 3.7	3.0 ± 1.8
Day 7 - T_{max}	2.3 ± 1.6	1.4 ± 1.5	2.8 ± 4.2	2.7 ± 2.0
Day 8 - H24:00	2.8 ± 2.2	2.2 ± 1.1	3.7 ± 2.7	1.8 ± 2.5
**LSD group scale (mm)**				
Baseline	-1.3 ± 0.8	-1.4 ± 1.8	-1.8 ± 0.4	-2.3 ± 0.8
Day 1 - T_{max}	-2.2 ± 0.8	-1.2 ± 1.8	-2.0 ± 0.6	-1.7 ± 1.4
Day 2 - H24:00	-1.2 ± 1.3	-0.8 ± 1.9	-2.0 ± 0.0	-1.8 ± 0.8
Day 7 - H00:00	-1.3 ± 0.8	-2.0 ± 0.7	-1.7 ± 1.0	-0.5 ± 3.4
Day 7 - T_{max}	-1.5 ± 1.0	-1.0 ± 1.9	-1.5 ± 1.4	-1.5 ± 1.0
Day 8 - H24:00	-1.7 ± 1.0	-1.4 ± 1.5	-1.5 ± 0.8	0.3 ± 3.3
**Morphine Benzedrine Group (MBG) Scale (mm)**				
Baseline	4.5 ± 2.7	6.8 ± 3.1	8.5 ± 4.3	6.7 ± 4.6
Day 1 - T_{max}	4.2 ± 3.0	5.4 ± 3.4	8.7 ± 4.8	4.0 ± 3.2
Day 2 - H24:00	3.4 ± 4.6	5.8 ± 2.9	5.0 ± 0.0	3.8 ± 2.2
Day 7 - H00:00	5.2 ± 5.0	4.0 ± 1.6	7.2 ± 4.7	5.8 ± 4.6
Day 7 - T_{max}	4.2 ± 3.1	3.6 ± 1.8	7.0 ± 4.0	5.8 ± 4.6
Day 8 - H24:00	4.3 ± 5.0	4.6 ± 1.3	8.0 ± 3.6	5.8 ± 6.1
**Pentobarbital Chlorpromazine Alcohol Group (PCAG) scale (mm)**				
Baseline	-1.8 ± 1.3	-1.4 ± 2.5	-2.5 ± 0.5	-2.0 ± 0.9
Day 1 - T_{max}	-0.5 ± 2.0	-0.8 ± 1.3	-2.8 ± 0.4	0.0 ± 2.6
Day 2 - H24:00	-1.4 ± 1.3	-1.0 ± 2.8	-2.0 ± 0.0	-0.3 ± 2.8
Day 7 - H00:00	-2.2 ± 1.2	-1.0 ± 3.9	-0.5 ± 4.7	-0.7 ± 2.2
Day 7 - T_{max}	-1.2 ± 1.5	1.4 ± 5.6	-1.3 ± 2.7	-0.5 ± 2.8
Day 8 - H24:00	-2.2 ± 1.2	-0.8 ± 4.4	-2.8 ± 0.4	2.3 ± 4.0

Supplementary Table 17. "ARCI 49" psychometrics test in the MAD study.
Summary descriptive statistics for the ARCI 49 sub-scores. No relevant trend was observed in any of the five sub-scores.

Study	Adminis-tration day	Dose (mg/day)	n	C_{max} (ng/mL) Mean (±SD)	t_{max} (h) Median (Range)	$AUC_{0\text{-}last}$ (mg/L×h) Mean (±SD)	$t_{½}$ (h) Min-max
SAD	Day 1	0.2	6	1.3 ± 0.3	3.0 (1.5-6.0)	6.7 ± 2.0[(1)]	ND[(2)]
		0.6	6	3.5 ± 1.0	2.5 (2.0-4.1)	29.7 ± 9.9[(5)]	75.9-228.7[(3), (4)]
		2	6	14.7 ± 4.3	3.0 (2.0-6.1)	114.5 ± 30.8[(5)]	84.4-179.6[(3), (4)]
		6	6	38.6 ± 8.1	2.0 (2.0-4.0)	287.0 ± 54.0[(5)]	69.3-162.5[(3), (4)]
MAD	Day 1	0.6	6	4.6 ± 1.4	3.0 (1.5-4.0)	23.8 ± 4.4[(7)]	ND[(6)]
		2	6	11.6 ± 3.7	3.0 (2.0-6.0)	68.6 ± 30.9[(7)]	ND[(6)]
		6	6	35.9 ± 13.5	3.5 (2.0-6.0)	256.5 ± 55.1[(7)]	ND[(6)]
	Day 7	0.6	6	3.3 ± 0.3	3.0 (2.0-4.0)	62.7 ± 9.5[(5)]	153.3-251.9[(4)]
		2	6	14.5 ± 4.9	3.0 (1.5-4.0)	273.1 ± 102.0[(5)]	151.9-240.5[(4)]
		6	6	37.1 ± 7.3	2.5 (2.0-4.0)	834.5 ± 343.5[(5)]	159.5-258.3[(4)]

Supplementary Table 18. Plasma pharmacokinetic parameters after single and repeated AEF0117 *per os* administration in healthy volunteers.

SAD: single ascending dose. MAD: multiple ascending doses.

(1) For the first cohort (0.2 mg), $AUC_{0\text{-}last}$ was calculated over a shorter period (0-12 h) than for the other cohorts (0-144 h) because of the LLOQ of 0.1 ng/mL of the method that was used. For the other cohorts a more sensitive method with a LLOQ of 10 pg/mL was used.

(2) For the first cohort (0.2 mg), the terminal elimination half-life ($t_{½}$) was not evaluable because concentrations beyond 24 h could not be assessed due to the LLOQ of 0.1 ng/mL of the method that was used.

(3) The half-life for the SAD cohorts was evaluated over a shorter period of time (0-144 h) and are thus underestimated compared to the half-life estimates for the MAD cohorts (evaluated between 0 and 264 h).

(4) The half-life for these cohorts were not determined over a sufficient interval (i.e., 1.5 x t½). For this reason, no descriptive statistics could be computed but the minimum and maximal values are described in the table for each dose.

(5) For the 0.6, 2, 6 mg doses of AEF0117, $AUC_{0\text{-}last}$ in the SAD study was calculated over a period (0-144 h) that was shorter than the one used (0-264 h) for calculating the $AUC_{0\text{-}last}$ after the last administration (D7) of AEF0117 in the MAD study.

(6) For the first administration of the MAD study (D1) the terminal elimination half-life (t½) was not evaluable because a new oral administration was done every 24 h.

(7) $AUC_{0\text{-}last}$ after the first administration (D1) was calculated over a much shorter interval (0-24 h) than the one calculated (0-264 h) after the last administration (D7).

Treatment/dose	Time after THC	Intoxication subscale			Felt good cannabis effect item			Cannabis cigarette liking item		
		**Mean**	**±**	**SEM**	**Mean**	**±**	**SEM**	**Mean**	**±**	**SEM**
**Placebo 0.06 mg**	**20 min**	61.5	±	3.5	81.1	±	1.9	76.7	±	2.7
	**40 min**	57.5	±	3.6	81.0	±	1.9	77.0	±	2.4
	**60 min**	54.5	±	3.6	75.4	±	2.8	74.5	±	3.2
	**90 min**	48.9	±	4.1	75.8	±	2.8	73.9	±	2.8
	**105 min**	47.8	±	4.3	75.0	±	2.7	74.9	±	2.8
**AEF 0.06 mg**	**20 min**	63.7	±	2.9	77.9	±	1.9	76.2	±	2.6
	**40 min**	58.8	±	3.3	76.1	±	2.3	74.9	±	2.4
	**60 min**	53.9	±	3.5	72.7	±	2.7	72.6	±	2.7
	**90 min**	47.1	±	4.0	75.8	±	2.0	73.5	±	2.5
	**105 min**	44.4	±	4.0	75.0	±	1.9	73.2	±	2.4
**Placebo 1 mg**	**20 min**	68.3	±	3.0	74.2	±	2.6	64.5	±	4.3
	**40 min**	65.1	±	2.9	72.7	±	2.7	61.5	±	4.3
	**60 min**	59.6	±	3.3	68.5	±	3.3	62.1	±	4.9
	**90 min**	56.6	±	3.6	65.4	±	3.5	59.7	±	4.2
	**105 min**	51.8	±	3.7	64.3	±	3.6	59.0	±	4.3
**AEF 1 mg**	**20 min**	66.8	±	3.3	66.8	±	3.5	61.7	±	4.1
	**40 min**	61.5	±	3.6	62.4	±	3.8	57.8	±	4.2
	**60 min**	57.2	±	3.7	58.0	±	4.5	54.8	±	4.6
	**90 min**	48.2	±	3.8	56.1	±	4.3	54.0	±	4.5
	**105 min**	43.7	±	3.9	54.7	±	4.3	54.4	±	4.3

Supplementary Table 19. Global crossover data from the Phase 2a study for the subjective effects of cannabis.
Summary descriptive statistics for the "Intoxication" subscale and for the "Felt good cannabis effect" and "Cannabis cigarette liking" items of the CRF in the Phase 2a study from Fig. 3a-c.

Treatment/dose	Time after THC	Intoxication subscale			Felt good cannabis effect item			Cannabis cigarette liking item		
		**Mean**	**±**	**SEM**	**Mean**	**±**	**SEM**	**Mean**	**±**	**SEM**
**Placebo**	**20 min**	75.7	±	2.6	85.0	±	1.9	77.9	±	3.3
	**40 min**	72.0	±	2.6	84.5	±	2.0	76.0	±	3.4
	**60 min**	67.8	±	2.8	81.2	±	2.4	77.5	±	3.8
	**90 min**	63.4	±	3.4	82.2	±	2.2	75.6	±	3.4
	**105 min**	59.7	±	3.7	80.3	±	2.5	75.8	±	3.4
**AEF 0.06 mg**	**20 min**	57.1	±	4.1	73.0	±	2.6	68.3	±	3.9
	**40 min**	50.9	±	4.0	70.2	±	2.3	65.5	±	3.1
	**60 min**	44.4	±	3.9	65.7	±	2.6	62.3	±	3.0
	**90 min**	38.0	±	4.2	67.4	±	2.3	61.8	±	3.0
	**105 min**	37.8	±	4.7	68.2	±	1.9	63.1	±	2.5
**AEF 1 mg**	**20 min**	67.0	±	4.3	67.9	±	4.8	63.9	±	6.3
	**40 min**	58.9	±	5.0	60.6	±	5.1	59.3	±	6.2
	**60 min**	54.5	±	5.2	53.3	±	6.6	54.5	±	7.0
	**90 min**	45.5	±	5.9	50.7	±	6.6	55.7	±	6.8
	**105 min**	40.8	±	6.2	52.3	±	6.4	55.9	±	6.5

Supplementary Table 20. Parallel group data from Phase 2a study for the subjective effects of cannabis.
Summary descriptive statistics for the “Intoxication” subscale and for the “Felt good cannabis effect” and “Cannabis cigarette liking” items of the CRF in the Phase 2a study from Fig. 3d-f.

Dose group	Day	AEF0117 Mean (SEM)	Placebo Mean (SEM)	AEF0117-Placebo
0.06 mg Cohort	**Day 1**	66.9 (4.3)	72.9 (4.2)	-5.9
	**Day 2**	67.4 (5.8)	70.5 (4.2)	-3.1
	**Day 3**	64.4 (6.0)	71.0 (4.1)	-6.6
	**Day 4**	63.9 (5.6)	71.8 (4.3)	-7.8
	**Day 5**	64.0 (5.8)	70.0 (3.7)	-6.0
1 mg Cohort	**Day 1**	71.9 (5.5)	68.0 (5.5)	3.9
	**Day 2**	71.4 (5.2)	70.0 (4.9)	1.3
	**Day 3**	71.1 (5.6)	71.0 (5.1)	0.1
	**Day 4**	67.0 (5.9)	73.0 (5.7)	-6.0
	**Day 5**	69.5 (6.6)	71.1 (4.8)	-1.6

Supplementary Table 21: Ratings of the “Social” subscale after smoking cannabis.
