# Supplementary material for: Signaling-specific inhibition of the CB1 receptor for cannabis use disorder: phase 1 and phase 2a randomized trials
Source: Nat Med. 2023 Jun 8;29(6):1487–99. doi: 10.1038/s41591-023-02381-w (PMC10287566; doi:10.1038/s41591-023-02381-w)
Supplement: Supplementary file 2 — Reporting Summary [file 41591_2023_2381_MOESM2_ESM.pdf]

nature portfolio

Corresponding author(s): Pier Vincenzo Piazza

Last updated by author(s): 17/04/2023

Reporting Summary

Nature Portfolio wishes to improve the reproducibility of the work that we publish. This form provides structure for consistency and transparency in reporting. For further information on Nature Portfolio policies, see our Editorial Policies and the Editorial Policy Checklist.

Statistics

For all statistical analyses, confirm that the following items are present in the figure legend, table legend, main text, or Methods section.

n/a	Confirmed	
☐	☒	The exact sample size (*n*) for each experimental group/condition, given as a discrete number and unit of measurement
☐	☒	A statement on whether measurements were taken from distinct samples or whether the same sample was measured repeatedly
☐	☒	The statistical test(s) used AND whether they are one- or two-sided<br>*Only common tests should be described solely by name; describe more complex techniques in the Methods section.*
☒	☐	A description of all covariates tested
☐	☒	A description of any assumptions or corrections, such as tests of normality and adjustment for multiple comparisons
☐	☒	A full description of the statistical parameters including central tendency (e.g. means) or other basic estimates (e.g. regression coefficient) AND variation (e.g. standard deviation) or associated estimates of uncertainty (e.g. confidence intervals)
☐	☒	For null hypothesis testing, the test statistic (e.g. *F*, *t*, *r*) with confidence intervals, effect sizes, degrees of freedom and *P* value noted<br>*Give P values as exact values whenever suitable.*
☒	☐	For Bayesian analysis, information on the choice of priors and Markov chain Monte Carlo settings
☒	☐	For hierarchical and complex designs, identification of the appropriate level for tests and full reporting of outcomes
☒	☐	Estimates of effect sizes (e.g. Cohen's *d*, Pearson's *r*), indicating how they were calculated

Our web collection on statistics for biologists contains articles on many of the points above.

Software and code

Policy information about availability of computer code

Data collection	Preclinical data: MED-PC software package (MED Associates, East Fairfield, VT) version 4.1; SR-LAB Startle Response System software (San Diego Instruments, San Diego, CA) version 6300-000-A; Azur, version 4.6 (Datalys, St Martin D'Heres, France); Datlab software, version 7.4.0.4 (Oroboros Instruments, Innsbruck, Austria); Microbeta2 Windows Workstation, version 2.2.0.19 (PerkinElmer, Waltham, MA); EnSpire Manager software, version 4.13.3005.1482 (PerkinElmer, Waltham, MA); Incucyte ZOOM 2014A (Sartorius, Goettingen, Germany); pClamp software, release 8.2 (AXON instruments; Molecular Devices LLC., San Jose, CA); RS/1 software, release 6.3 (Applied Materials, Santa Clara, CA); ART computerized acquisition system, release 4.33 (Data Sciences International, New Brighton, MN); Ethovision XT version 12 (Noldus Information Technology, Wageningen, The Netherlands); BD Pathway software suite (Becton Dickinson) version 1.6; RS/1 software, release 6.3 (Applied Materials, Santa Clara, CA); ART computerized acquisition system, release 4.33 (Data Sciences International, New Brighton, MN); Ethovision XT version 12 (Noldus Information Technology, Wageningen, The Netherlands); NONMEM computer program version 7.4.1 (ICON).<br><br>Bioanalysis: Thermo Xcalibur version 2.0.7 or Xcalibur Access version 2.2 (ThermoFisher, Waltham, MA); Analyst software version 1.5 or 1.6 (AB Sciex, Framingham, MA).<br><br>Clinical data: Inquisit (Millisecond Software, LLC, Seattle, WA) version 5.0.13; Panther-EDC version 3.70.3 (EDETEK Inc., Princeton, NJ); Clincase version 2.6 (Quadratek Data Solutions Ltd, Berlin, Germany).
Data analysis	Prism versions 6.0 or 9.5.0 (GraphPad, San Diego, CA); Statistica versions 6.0, 8.0 or 9.0 (StatSoft Europe, Hamburg, Germany); SigmaPlot version 12.5 (Systat Software Inc., San Jose, CA); SAS version 9.3 or 9.4 (SAS Institute Inc., Cary, North Carolina); Phoenix WinNonLin version 6.3 (Certara USA Inc., Princeton, NJ).

For manuscripts utilizing custom algorithms or software that are central to the research but not yet described in published literature, software must be made available to editors and reviewers. We strongly encourage code deposition in a community repository (e.g. GitHub). See the Nature Portfolio guidelines for submitting code & software for further information.

Data

Policy information about availability of data

All manuscripts must include a data availability statement. This statement should provide the following information, where applicable:

- Accession codes, unique identifiers, or web links for publicly available datasets
- A description of any restrictions on data availability
- For clinical datasets or third party data, please ensure that the statement adheres to our policy

All data are available in the main text, extended figures and supplementary data. For privacy reasons, individual participant data pertaining the clinical trials reported in this article will be provided, after de-identification, upon request to qualified scientific researchers who provide methodologically sound and justified research proposals and could be submitted to a CDA or an MTA, depending on the data (for this purpose, please contact the corresponding author). Access to at least the minimum data from the clinical trials and/or related documents that are necessary to carry the proposed research will be granted within a reasonable period, which according to the request can range from one to three months, and for a pre-specified amount of time and through a secure server depending on the nature of the research plan.

Human research participants

Policy information about studies involving human research participants and Sex and Gender in Research.

Reporting on sex and gender	Findings in this report apply to all sexes/genders although most of the subjects included in these studies self-identified as males on the recruitment form. Gender or sex differences were not considered in the study design. Consent was obtained for sharing individual-level data with personally identifiable information only with specific companies and government agencies and with non-personally identifiable information in all other cases. Gender- or sex-based analyses could not be performed also because of the predominance of male subjects in the studies. This sex imbalance is likely due to the restrictive inclusion criterion for contraception for women, implemented because of the early phase testing of AEF0117.<br>- NCT03717272: 28M, 1F<br>- NCT03325595 (SAD): 36M, 4F<br>- NCT03443895 (MAD): 22M, 2F
Population characteristics	No covariates were used in the analysis of the clinical data.<br>- NCT03325595 (SAD): Mean (SD) age of the subjects was 36.8 (9.6) years, ranging from 22 to 55 years. Mean BMI of the subjects at study entry was 25.2 (2.6) kg/m2.<br>A majority of the subjects were black or African American (85.0% of the overall population). The ethnicity of the overall population was 90% non-Hispanic or Latino for 90.0% .<br>Fifteen (15) out of 40 subjects (37.5%) reported a medical history. All reported events were resolved and compatible with study participation.<br><br>- NCT03443895 (MAD): Mean (SD) age of the subjects was 38.1 (10.1) years, ranging from 20 to 55 years. Mean BMI of the subjects at study entry was 25.7 (2.7) kg/m2.<br>A majority of the subjects were black or African American (66.7% of the overall population). the ethnicity of the over all population was 83.3% non-Hispanic or Latino.<br>Seven out of 24 subjects (29.2%) reported a medical history. All reported events were resolved and compatible with study participation.<br><br>- NCT03717272 (phase 2a): Mean (SD) age of the subjects was 32.5 (6.3) years, ranging from 21 to 44 years. Mean BMI of the subjects at study entry was 24.3 (3.6) kg/m2. At study entry, subjects had a mean (SD) number of days of cannabis use per week of 6.9 (0.4) ranging between 5 and 7, and subjects used a mean (SD) of 2.85 (1.79) grams of cannabis per day, ranging from 1 to 8. Overall, the 4 groups of subjects (cohort 0.06 mg AEF first or placebo first, and cohort 1 mg AEF or placebo first) were reasonably well balanced. 58.6% of the subjects were black or African American. The ethnicity of the overall population was 58.6% non-Hispanic or Latino.<br>A total of 12 (41.4%) of the subjects included in the study had a significant event in their medical history. All reported events were resolved and compatible with study participation.
Recruitment	- NCT03325595 (SAD) and NCT03443895 (MAD): Participants were recruited by the CRO Biotrial primarily through advertisements and their website. Patients meeting the entry criteria who are known or referred to the study center were eligible for enrollment. There were no potential self-selection or other biases affecting recruitment.<br>- NCT03717272 (phase 2a): Eligible participants were recruited from the New York, NY area. Recruitment for this study occurred primarily through advertisements in local newspapers, online through Craigslist, and by word of mouth. Interested individuals responded to advertisements by calling the lab for information about the study. There were no potential self-selection or other biases affecting recruitment.
Ethics oversight	- NCT03325595 (SAD) and NCT03443895 (MAD): The study protocol, the subject information sheet, the informed consent, the amended protocols and Informed Consent Forms (ICFs), were submitted to IntegReview IRB, an independent IRB located in 3815 S. Capital of Texas Hwy, Suite 320, Austin, TX 78704, and to the New York State Psychiatric Institute - Columbia University Department of Psychiatry Institutional Review Board. They were approved before the start of the study.<br><br>- NCT03717272 (phase 2a): The study protocol and any information supplied to the subjects of the study to obtain their informed consent, including written ICFs, subject recruitment procedures (e.g., advertisements) and written information to be provided to subjects (information leaflets), have been reviewed and approved by the qualified IRB/IEC:

New York State Psychiatric Institute - Columbia University Department of Psychiatry Institutional Review Board. Prior to initiation of the study (before the enrolment of the subjects), the Sponsor received the documentation of the IRB approval, which specifically identified the study/protocol, and a list of the committee members. Amendments to the protocol and revisions to the informed consent were also submitted to and, if required, approved by the IRB.

All the clinical trial protocols were approved by the Food and Drug Administration (FDA).

Note that full information on the approval of the study protocol must also be provided in the manuscript.

Field-specific reporting

Please select the one below that is the best fit for your research. If you are not sure, read the appropriate sections before making your selection.

☒ Life sciences ☐ Behavioural & social sciences ☐ Ecological, evolutionary & environmental sciences

For a reference copy of the document with all sections, see nature.com/documents/nr-reporting-summary-flat.pdf

Life sciences study design

All studies must disclose on these points even when the disclosure is negative.

Sample size

Preclinical studies: Sample sizes for all in vivo and in vitro experiments were chosen based on preliminary data obtained by the experimenters, standard sample sizes used in the respective fields, or were based on the sample sizes used in similar published studies (e.g., Busquets-Garcia A et al. Pregnenolone blocks cannabinoid-induced acute psychotic-like states in mice. Mol Psychiatry. 2017;22(11):1594-1603).

Clinical studies:
- NCT03325595 (SAD) and NCT03443895 (MAD): These are descriptive studies and the sample sizes selected are not based on power analysis.

- NCT03717272 (phase 2a): For this study the sample size considerations apply only to the inferential part of the study, i.e., the comparison between AEF0117 and Placebo of the primary endpoint that would be performed after the first two doses and at the end of the study when all the doses have been tested using, in both cases, an MMRM analysis. The clinical hypothesis underlying the calculation of this sample size was that AEF0117 would decrease the subjective effects of cannabis as measured in particular by the "Good drug effect". The expected treatment effect and variability was extrapolated from other studies in subjects with CUD, as the current study was the first one to assess a drug with this mechanism of action in subjects with CUD. The sample size estimation was based on a t-test for paired samples under the assumption that the within subjects' correlation is 0.50 and the standard deviation within treatment-period is 35 mm. Using 12 subjects, a difference of 36 mm could be demonstrated with 90% power. Of note, a statistically significant effect (two-sided p-value ≤ 0.05) could be shown, should a mean difference of 21.7 mm be observed with a standard deviation of 35 mm.

Data exclusions

No human or animal samples for which measurements were performed were excluded from analyses. Individual data points may have been excluded because of sampling error or assay failure but no samples were excluded from analyses after completion of sample processing and measurements.

Replication

Mitochondrial respiration results were replicated in n=3 independent experiments. p-ERK measurements in STHdh Q7/Q7 cells were replicated in n=4 independent experiments. Replication was not attempted for the results of other preclinical or clinical studies.

Randomization

Preclinical studies: Age- and sex-matched animal subjects were randomly assigned to experimental or control groups, except for the study on the effects of repeated treatment with AEF0117 on food intake and body weight, where group allocation was aided by the evaluation of mice body composition in vivo using an Echo MRI 900 (EchoMedical Systems, Houston, Texas, USA). In vitro samples were randomly allocated to experimental groups.

- NCT03325595 (SAD) and NCT03443895 (MAD): The subjects were given a screening number after signing the informed consent in chronological order. Subjects were randomly assigned within a dose escalation cohort to active AEF0117 or placebo in a 6 active:2 placebo ratio, according to the randomization list generated by a Biotrial statistician at the time of dosing.

- NCT03717272 (phase2a): A computer-generated randomization schedule was prepared by a statistical programmer not directly involved in the conduct of the study and IMP was packaged in a double-blind manner. Subjects were randomly assigned within a dose-escalation cohort to one of two treatment sequences: Group 1, AEF0117 during Period A, and Placebo during Period B; or Group 2, AEF0117 during Period B and Placebo during Period A.

Blinding

In all behavioral studies in rodents the experimenters were blinded to the group allocation of the animals. The experimental designs did not allow blinding for group allocation for in vitro, toxicology or studies with monkeys.

All experiments with human subjects were double blinded:

- NCT03325595 (SAD) and NCT03443895 (MAD): The pharmacist and his/her attendant were the only personnel to have access to the randomization list in order to prepare the drug for administration.

- NCT03717272 (phase 2a): Research subjects were advised that they would receive both active and placebo study medication but were blinded as to whether they received AEF0117 or placebo during Periods A and B. Research staff that interacted with study subjects was also blinded as to whether a subject was receiving AEF0117 or placebo during Periods A and B. Principal Investigator was blinded to the treatment identity during the conduct of each cohort until receipt of an unblinded interim report after the database closure/finalization of that cohort.

Reporting for specific materials, systems and methods

We require information from authors about some types of materials, experimental systems and methods used in many studies. Here, indicate whether each material, system or method listed is relevant to your study. If you are not sure if a list item applies to your research, read the appropriate section before selecting a response.

Materials & experimental systems

n/a	Involved in the study
☐	☒ Antibodies
☐	☒ Eukaryotic cell lines
☒	☐ Palaeontology and archaeology
☐	☒ Animals and other organisms
☐	☒ Clinical data
☒	☐ Dual use research of concern

Methods

n/a	Involved in the study
☒	☐ ChIP-seq
☒	☐ Flow cytometry
☒	☐ MRI-based neuroimaging

Antibodies

Antibodies used	Phospho-Histone H2A.X (Ser139) (20E3) Rabbit mAb (Alexa Fluor® 488 Conjugate), Cell Signaling Technology, #9719.
Validation	Tested in-house by the provider for direct flow cytometry and immunofluorescent analysis in human cells. Representative publication: Tanabe M, et al., J Reprod Dev. 2015;61(1):35-41. doi:10.1262/jrd.2014-105

Eukaryotic cell lines

Policy information about cell lines and Sex and Gender in Research

Cell line source(s)	- HEK293 cells (ATCC Cat# CRL-1573, RRID:CVCL_0045; batch 59534772)<br>- CHO-hCB1 cells (# ES-110C, Perkin Elmer; USA)<br>- STHdhQ7/Q7 cells (Coriell Cat# CH00097, RRID:CVCL_M590)<br>- HeLa cells (ATCC)<br>- Primary cortical neurons from E19 rat embryos (Wistar)<br>- Primary rat hepatocytes from 10-12 weeks old Wistar male rats<br>- Human lymphocytes were taken from young (~18-35 years old) healthy non-smoker subjects
Authentication	None of the cell lines used were authenticated
Mycoplasma contamination	Cell lines were not tested for mycoplasma contamination
Commonly misidentified lines<br>(See ICLAC register)	No commonly misidentified cell lines where used in any of the studies

Animals and other research organisms

Policy information about studies involving animals; ARRIVE guidelines recommended for reporting animal research, and Sex and Gender in Research

Laboratory animals	- Squirrel monkey (Saimiri Sciureus), 800-1100 g; 14-17 years old; from in-house colony, originally from NIH Animal Center, Poolesville, MD 20837<br>- CD-1 Swiss mice (Mus musculus), 8-10 weeks old<br>- C57BL/6N mice (Mus musculus), 8-10 weeks old<br>- CB1-flox (CB1 f/f) mice (Mus musculus, strain C57BL/6N), 8 weeks old<br>- C57BL/6J mice (Mus musculus), 8-9 weeks old<br>- Sprague-Dawley rats (Rattus norvegicus), 6-9 weeks old<br>- Wistar rats (Rattus norvegicus), 6-11 weeks old<br>- Beagle dogs (Canis familiaris), 7-8 and 17-40 months old
Wild animals	No wild animals were used in these studies
Reporting on sex	The following experiments involved animals of both sexes and the results are reported separately, where appropriate: toxicology and safety pharmacology experiments in rats and dogs; measurement of plasma and brain concentrations of AEF0117 in mice, rats and dogs; 91-day repeated oral toxicity study in rats and dogs; and the effects of AEF0117 on glucocorticoid secretion in mice. Only male animals were used for all other experiments.
Field-collected samples	These studies did not involve samples collected from the field

Ethics oversight

- French Ministry of Agriculture and Fisheries
- Ethical Committee of the University of Bordeaux
- Ethical Committee for Animal Research (CEEA-PRBB); University Pompeu Fabra, Barcelona, Spain.
- Institutional Animal Care and Use Committee of the Intramural Research Program, NIDA, NIH, DHHS
- Oncodesign Internal Ethical Committee
- European Parliament guidelines

Note that full information on the approval of the study protocol must also be provided in the manuscript.

Clinical data

Policy information about clinical studies

All manuscripts should comply with the ICMJE guidelines for publication of clinical research and a completed CONSORT checklist must be included with all submissions.

Clinical trial registration

ClinicalTrials.gov Identifiers: NCT03717272, NCT03325595 (SAD) and NCT03443895 (MAD)

Study protocol

Full protocols were provided with submission. All relevant information is available at ClinicalTrials.gov.

Data collection

- NCT03325595 (SAD) location: Biotrial Inc., Newark, New Jersey, United States, 07103. Study Start Date: April 6, 2017; Primary Completion Date: February 26, 2018; Study Completion Date: February 26, 2018.
- NCT03443895 (MAD) location: Biotrial Inc., Newark, New Jersey, United States, 07103. Study Start Date: January 8, 2018; Primary Completion Date: May 1, 2018; Actual Study Completion Date: May 1, 2018.
- NCT03717272 (phase 2a) location: Substance Use Research Center New York, New York, United States, 10032. Study Start Date: October 23, 2018; Primary Completion Date: July 31, 2020; Actual Study Completion Date: January 1, 2021.

Outcomes

NCT03325595 (SAD) and NCT03443895 (MAD): Primary objectives were to evaluate the safety and tolerability of escalating single and multiple oral doses of AEF0117 in healthy male and female volunteers. Secondary objectives were to evaluate the PK of escalating single and multiple oral doses of AEF0117 in healthy male and female volunteers, and to evaluate the PD effects of escalating single and multiple oral doses of AEF0117 on plasma PREG, dehydroepiandrosterone (DHEA), allopregnanolone, testosterone and endocannabinoids [N-arachidonoylethanolamine (AEA) and 2-arachidonoylglycerol (2AG)] and serum estradiol, progesterone and cortisol in healthy male and female volunteers.
Safety and tolerability of AEF0117, including dose-limiting toxicities (DLTs), were assessed by monitoring incidence of treatment-emergent AEs and SAEs overall and by grade intensity and by evaluating changes from baseline in: vital signs, ECGs, clinical laboratory values from blood, urine samples, psychometric and C-SSRS tests.
Single dose AEF0117 pharmacokinetic parameters (Cmax, Cmin, tmax, t1/2, AUC0-t, AUC0-∞,%AUCextrap, CL/F, Vd/F) will be determined based on serial blood sample collections and plasma AEF0117 concentration.
Effects of AEF0117 on plasma pregnenolone, 17-OH-pregnenolone, DHEA, allopregnanolone, testosterone and endocannabinoids (AEA and 2AG) and serum estradiol, progesterone and cortisol concentrations were measured at pre-dose and 0.5, 1, 2, 4, 8, 12, 24 and 48 hours post-dose.

- NCT03717272 (phase 2a): The study was designed to evaluate the safety, tolerability, pharmacodynamics (PD) and pharmacokinetics (PK) of multiple escalating oral doses of AEF0117 in adult male and non-pregnant female, non-treatment seeking, subjects with cannabis use disorder (CUD). The primary objective of this study aimed to test the effects of two to four doses of AEF0117 compared to placebo on the subjective effects of cannabis related to abuse liability "Good drug effect". The secondary objectives were to test the effects of AEF0117 compared to placebo on cannabis self-administration, on cannabis-induced analgesia and cognitive performance in CUD subjects.
Ratings of subjective effects of cannabis were performed using multiple items from two different instruments: 1) a 44-item VAS; and 2) the Cannabis Rating Form (CRF). Both instruments used visual analog scales (from 0 to 100 mm) to measure subjective effects. The perceived 'good effect' of cannabis was measured by: 1) A subscale of the 44-item VAS (primary endpoint), containing the arithmetic mean of two items, "I feel a good cannabis effect" and "I feel high" and by 2) two individual items of the CRF (key secondary endpoints), "Felt Good Cannabis effect" and cannabis " Liking". The subscale of the 44-item VAS was initially named in the protocol "Good Cannabis effect" subscale. Before starting the statistical analysis, this subscale was renamed "Intoxication" subscale to acknowledge that it combined the subjective perception of "good cannabis effects" and cannabis "high". The renaming was also deemed appropriate to more clearly differentiate the 44-item VAS subscale from the two individual items of the CRF that exclusively targeted "good cannabis effects".
Cannabis self-administration was measured by counting the number of cannabis puff purchased by the participants using its study stipend.
Cognitive performance was measured by a test battery that included the Sustained Attention to Response Task (SART), Behavioral Pattern Separation (BPS-O), Digital Substitution Task (DSST) and Stroop Color task.
The Cold Pressor Test (CPT)was used to assess the analgesic effects of cannabis and was performed only on Day 1 only of each dosing period.
